# Multiple recent horizontal transfers of the *cox1 *intron in Solanaceae and extended co-conversion of flanking exons

**DOI:** 10.1186/1471-2148-11-277

**Published:** 2011-09-27

**Authors:** Maria V Sanchez-Puerta, Cinthia C Abbona, Shi Zhuo, Eric J Tepe, Lynn Bohs, Richard G Olmstead, Jeffrey D Palmer

**Affiliations:** 1 Instituto de Ciencias Básicas, IBAM-CONICET and Facultad de Ciencias Agrarias, Universidad Nacional de Cuyo, Alte. Brown 500, Mendoza (5500), Argentina; 2 IBAM-CONICET and Facultad de Ciencias Agrarias, Universidad Nacional de Cuyo, Alte. Brown 500, Mendoza (5500), Argentina; 3 Department of Biology, Indiana University, 1001 E Third St., Bloomington (47405), USA; 4 Department of Biology, University of Utah, 257 South 1400 East, Salt Lake City (84112), USA; 5 Department of Biological Sciences, University of Cincinnati, Cincinnati (45221), USA; 6 Department of Biology, University of Washington, Hitchcock Hall 423, Seattle (98195), USA

**Keywords:** horizontal gene transfer, *cox1 *intron, Solanaceae, mitochondrial DNA, homing endonuclease

## Abstract

**Background:**

The most frequent case of horizontal transfer in plants involves a group I intron in the mitochondrial gene *cox1*, which has been acquired via some 80 separate plant-to-plant transfer events among 833 diverse angiosperms examined. This homing intron encodes an endonuclease thought to promote the intron's promiscuous behavior. A promising experimental approach to study endonuclease activity and intron transmission involves somatic cell hybridization, which in plants leads to mitochondrial fusion and genome recombination. However, the *cox1 *intron has not yet been found in the ideal group for plant somatic genetics - the Solanaceae. We therefore undertook an extensive survey of this family to find members with the intron and to learn more about the evolutionary history of this exceptionally mobile genetic element.

**Results:**

Although 409 of the 426 species of Solanaceae examined lack the *cox1 *intron, it is uniformly present in three phylogenetically disjunct clades. Despite strong overall incongruence of *cox1 *intron phylogeny with angiosperm phylogeny, two of these clades possess nearly identical intron sequences and are monophyletic in intron phylogeny. These two clades, and possibly the third also, contain a co-conversion tract (CCT) downstream of the intron that is extended relative to all previously recognized CCTs in angiosperm *cox1*. Re-examination of all published *cox1 *genes uncovered additional cases of extended co-conversion and identified a rare case of putative intron loss, accompanied by full retention of the CCT.

**Conclusions:**

We infer that the *cox1 *intron was separately and recently acquired by at least three different lineages of Solanaceae. The striking identity of the intron and CCT from two of these lineages suggests that one of these three intron captures may have occurred by a within-family transfer event. This is consistent with previous evidence that horizontal transfer in plants is biased towards phylogenetically local events. The discovery of extended co-conversion suggests that other *cox1 *conversions may be longer than realized but obscured by the exceptional conservation of plant mitochondrial sequences. Our findings provide further support for the rampant-transfer model of *cox1 *intron evolution and recommend the Solanaceae as a model system for the experimental analysis of *cox1 *intron transfer in plants.

## Background

Horizontal gene transfer (HGT) is surprisingly common in plant mitochondrial genomes, especially compared to plant chloroplast and nuclear genomes [1-6]. A notable case of HGT in plant mitochondria involves a "homing" group I intron present in the mitochondrial *cox1 *gene of many disparately related lineages of angiosperms. All relevant studies [7-15] concur that this intron most likely entered angiosperms only once, from a fungal donor. With one exception [15], treated in the Discussion, these studies have, in aggregate, led to the conclusion that the intron subsequently spread rampantly within angiosperms via HGT, with some 80 separate angiosperm-to-angiosperm transfers postulated [8-12] to account for the intron's distribution among the 833 angiosperms analyzed thus far. Three lines of evidence underlie the "rampant transfer" model for the evolution of the *cox1 *intron in angiosperms: A) the intron has a highly sporadic distribution among angiosperms, B) its phylogeny is strongly incongruent with angiosperm phylogeny, and, C) with notably rare exception, it co-occurs with a short, highly divergent "co-conversion tract" located immediately downstream of the intron.

**Figure 1:**
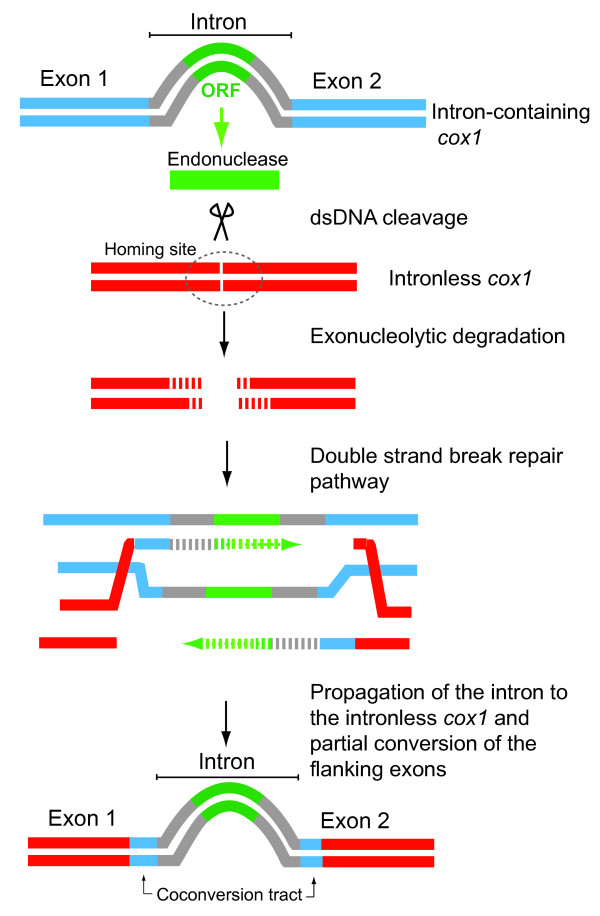
**Schematic mechanism of group I intron homing**. The *cox1 *intron invades an intron-lacking *cox1 *allele, with concomitant gene conversion of flanking exonic sequences.

Homing introns are regarded as highly mobile, invasive elements due to the properties of the site-specific DNA endonucleases that they encode, which facilitate intron propagation [16,17]. Homing endonucleases catalyze the integration of the intron, via the double-strand-break-repair pathway, into the target sequence (termed the "homing site") that is present in intron-lacking alleles of the intron's target gene (Figure 1). As a consequence of the degradation of the cleaved target sequence and subsequent repair process, part of the foreign exonic regions immediately flanking the invading intron often engages in a gene conversion activity that replaces part of the host gene's exonic sequence [16-20]. A region of converted exonic sequence is called a "co-conversion tract" (CCT).

Although comparative evidence indicates that the *cox1 *intron has a highly invasive history in plants, no experimental study has been reported on its transmission or mechanistic properties. This contrasts with the situation for certain other homing group I introns, including the cognate intron in yeast mitochondria, thanks to the well-developed genetic systems available in microbial models [16,18-20]. Human-engineered transformation of plant mitochondrial genomes is not yet feasible, despite many years of efforts and notable success in transforming chloroplasts [21]. This is paradoxical considering that natural transformation (via HGT) is relatively common in plant mitochondria [1-6], but unheard of in chloroplasts of land plants [22]. Classical genetics is also problematic, because mitochondria are almost always transmitted uniparentally (usually maternally) in sexual crosses in plants and because appropriately wide crosses are rarely successful. This leaves somatic cell genetics as the approach of choice for manipulating plant mitochondrial genomes. Cytoplasmic hybrid plants (cybrids) are created by fusing protoplasts from two different cultivars, species or genera and then generating whole plants from the fusion products. Plant cybrids can be made between relatively distantly related plants [23-27] and almost invariably contain recombinant mitochondrial genomes owing to the propensity of mitochondria to fuse with one another [28,29]. By analyzing cybrids that combine intron-containing and intron-lacking parents, one should be able to test the hypothesis that the angiosperm *cox1 *intron encodes a functional homing endonuclease, assess rates of intron colonization, and measure lengths of exonic CCTs.

The premier system for the efficient and large-scale production of cybrid plants is the Solanaceae, one of the largest (~2,500 species) and economically most important families of flowering plants (containing potato, tomato, chili pepper, eggplant, tobacco, petunia). Somatic genetics is best developed in tobacco (*Nicotiana tabacum*), the favored plant for chloroplast transformation and "biopharming" [21,30-32]. Many other species of Solanaceae also provide favorable material for somatic cell genetics, and cybrids can be successfully produced between relatively distantly related members of the family [23-27]. The mitochondrial genome of tobacco has been sequenced [33] and lacks the *cox1 *intron. Similarly, the six other diverse, previously examined representatives of the Solanaceae also lack this intron [9,10,12]. Therefore, to be able to exploit the family for somatic genetic studies of *cox1 *intron function, we surveyed over 400 diverse species of Solanaceae in order to find members with the intron.

The second goal of this study was to gain further insight into the evolutionary history of this exceptionally mobile genetic element. In particular, we wished to test two predictions that follow from the inferred evolutionary history of the *cox1 *intron. The first, which is predicated on the intron's frequent transfer within angiosperms [8-12], is that greatly increased sampling in a large family in which the intron has not been found based on current, scanty sampling will uncover multiple intron acquisitions within the family, with the intron-containing lineages embedded within clades that lack both the intron and its associated CCT. This prediction is obviously integral to the Solanaceae motivation of this study. Second, based on the apparent bias of *cox1 *intron transfer in plants toward phylogenetically local events [10,12], we predict that a significant fraction of the intron transfers discovered in the Solanaceae will turn out to be intrafamilial events.

## Results

### Intron presence-absence and phylogeny

**Figure 2:**
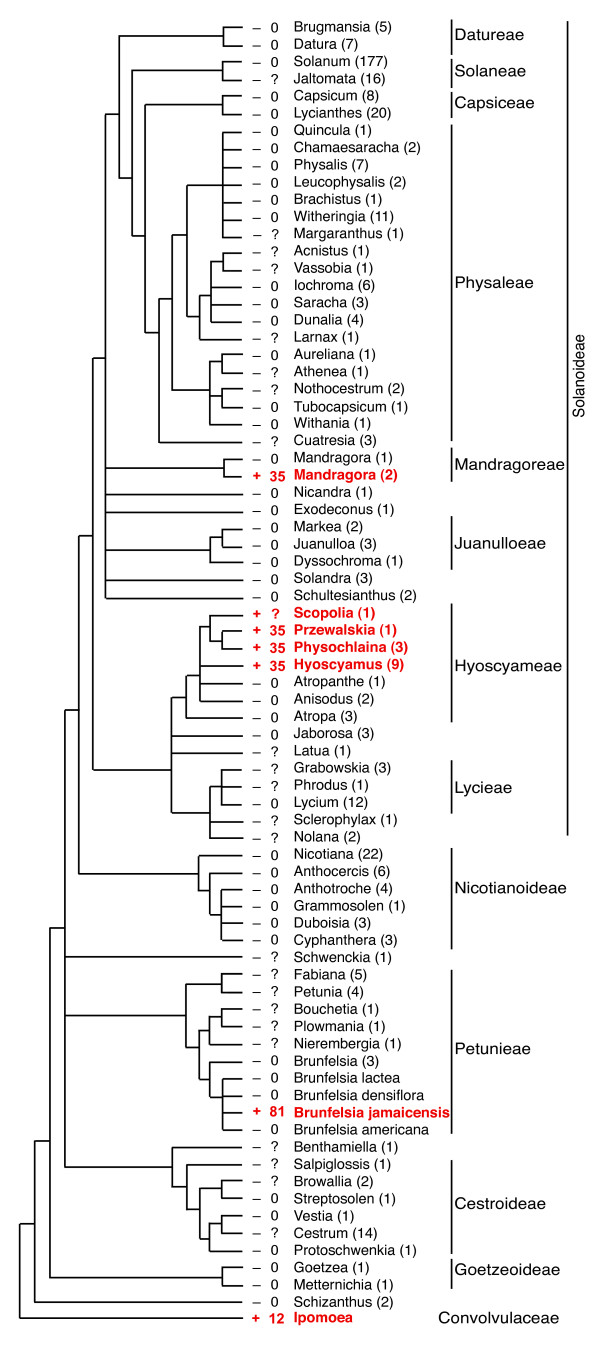
**Distribution of the *cox1 *intron in the Solanaceae**. Intron presence is indicated by red and "+" symbols, intron absence by black and "-" symbols. Numbers to the left of plant names give the minimum estimated size of the 3' CCT (question marks indicate that exons were not sequenced). Parenthetical numbers give the number of species sampled for each genus (see Additional File 1). The tree topology is based on refs [42; 48] and Additional File 4. Tribes are labeled as in Olmstead *et al *[42].

PCR was used to assess the presence/absence of an intron at the one site, near the middle of the *cox1 *gene, in which all previously described cases of introns in this gene in angiosperms have been found. This approach was facilitated by the conserved length (953-1,031 bp) of this intron in angiosperms [9,12], as well as by the generally highly conserved nature of plant mitochondrial sequences owing to very low rates of synonymous substitutions [34-36]. A total of 426 species (belonging to 70 genera) of the Solanaceae were examined (Figure 2; Additional File 1). The great majority were sampled as part of an initial screening, chosen to emphasize diversity across the family and based on DNA availability. A follow-up screening sampled more comprehensively within the three groups of Solanaceae that were found to contain the intron, as well as in taxa closely related to these groups.

Of the 426 species of Solanaceae examined, 409 (66 genera) gave a *cox1 *PCR product of the size (0.8 kb) expected for an intron-lacking gene, whereas 17 (6 genera) yielded a product of the size (1.8 kb) expected for an intron-containing gene (Figure 2). The 17 intron-containing species represent three phylogenetically disjunct lineages within the Solanaceae and include 14 species of Hyoscyameae (i.e., all examined members of *Hyoscyamus, Physochlaina, Przewalskia*, and *Scopolia*), 2 of 3 examined species of *Mandragora *(mandrake), and a single species of *Brunfelsia *(*B. jamaicensis*) out of 7 examined (Figure 2). Results for a number of the intron-containing species, including *B. jamaicensis*, were confirmed by sequencing multiple accessions from each of these species (Additional File 1).

Sequencing of the 0.8-kb product from 48 diverse species (43 genera) of Solanaceae (Figure 2) confirmed in all cases the absence of the intron. Sequencing of almost all 1.8-kb products confirmed that they contain an intron, located at the canonical angiosperm *cox1 *intron insertion site. All Solanaceae introns are 967 bp in length and contain a full-length and intact open reading frame of 840 bp encoding a putative homing group I endonuclease.

**Figure 3:**
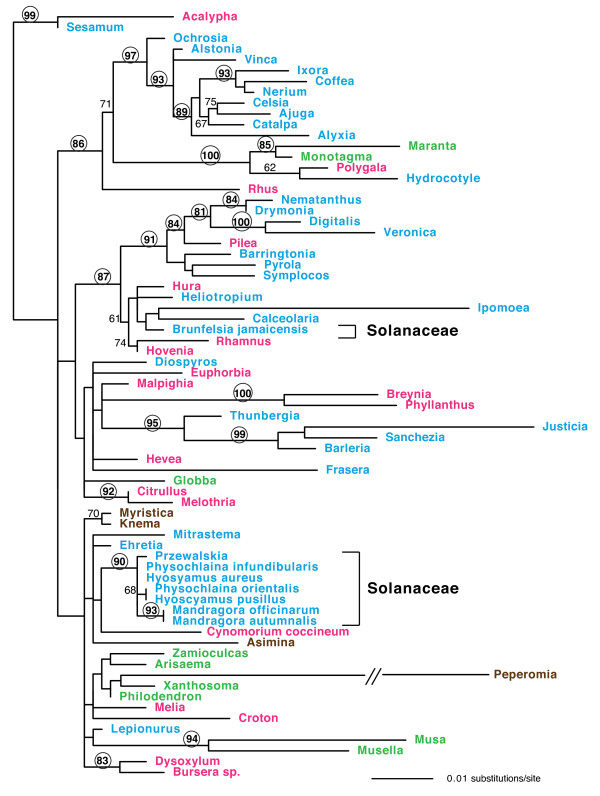
**Maximum likelihood phylogeny of angiosperm *cox1 *introns**. The data set includes 71 taxa and 947 nucleotides. Taxa in red are rosids; blue, superasterids; green, monocots; and brown, magnoliids. Numbers above branches are bootstrap support values > 60%, with values > 80% circled. The tree is rooted as described in Sanchez-Puerta *et al*. (2008).

**Figure 4:**
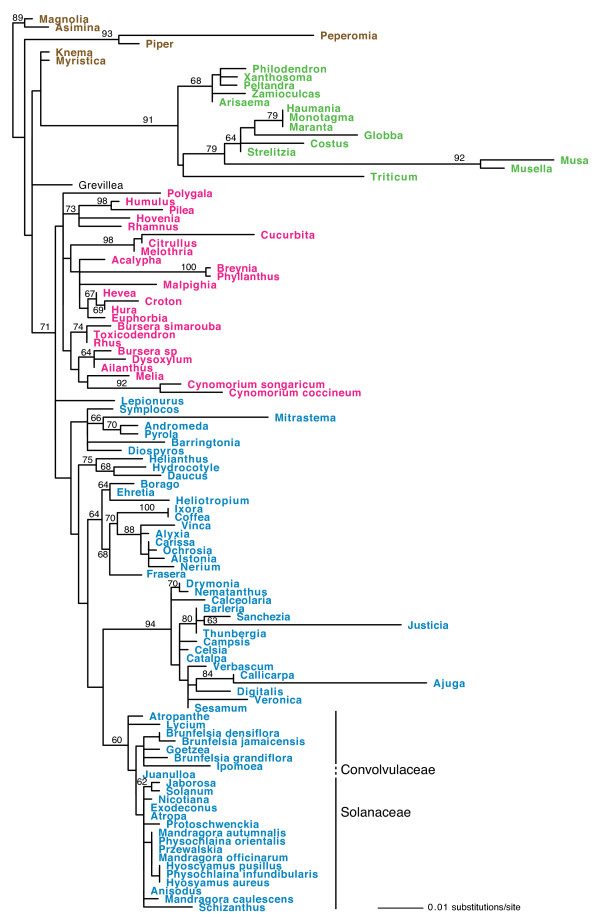
**Maximum likelihood phylogeny of angiosperm *cox1 *exons**. The data set includes 108 taxa and 1,263 nucleotides. Taxa in red are rosids; blue, superasterids; green, monocots; and brown, magnoliids. Numbers above branches are bootstrap support values > 60%.

The Solanaceae *cox1 *introns were subjected to phylogenetic analyses as part of a data set that included 63 previously reported *cox1 *introns from a wide range of angiosperms. As discussed in detail previously [9,12], the *cox1 *intron phylogeny is highly incongruent with angiosperm phylogeny (Figure 3). This incongruence is most vividly depicted by the extensive interspersion of colors on the intron tree (used to distinguish taxa belonging to four ancient, major, and well-distinguished groups of angiosperms), and contrasts markedly with the organismal-congruence of a phylogeny (Figure 4) based on *cox1 *exon sequences from 108 diverse angiosperms, including all those included in Figure 3. To highlight just one example of the incongruence between *cox1 *intron and organismal phylogeny, note the 100% bootstrap support for a clade containing introns from the asterid *Hydrocotyle*, the rosid *Polygala*, and the monocots *Maranta *and *Monotagma *(Figure 3).

The Solanaceae introns show evidence of both congruence and incongruence with angiosperm phylogeny. Two of the three clades of Solanaceae introns - the Hyoscyameae and *Mandragora *clades - form a strongly supported (90% bootstrap support) monophyletic group, whereas the *Brunfelsia jamaicensis *intron is only distantly related to these other Solanaceae introns (Figure 3).

### Co-conversion tracts

**Figure 5:**
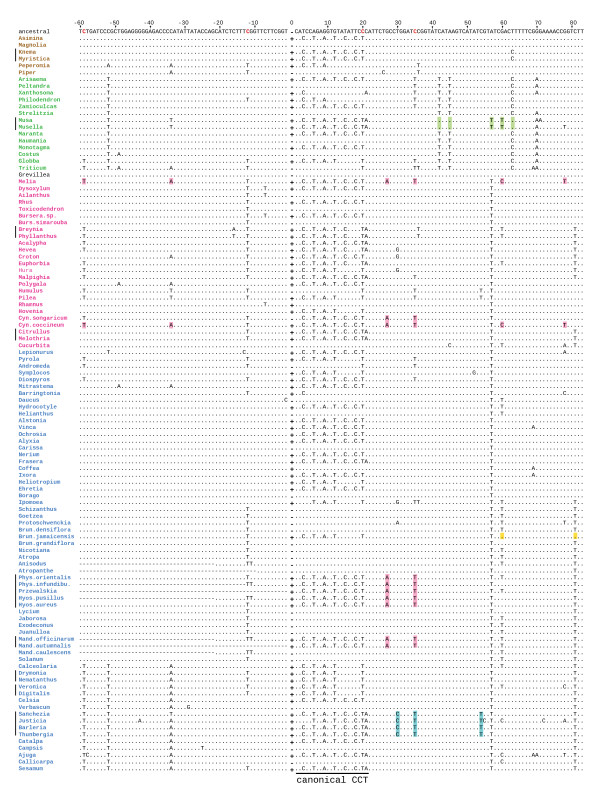
**Nucleotide alignment of *cox1 *exonic regions immediately flanking the intron insertion site**. Taxa were chosen to represent the broad diversity of *cox1 *intron types/lineages known among angiosperms, with space constraints allowing only a small number of intron-lacking *cox1 *genes to be included. Among the latter genes, the Solanaceae are over-represented. Taxa are in phylogenetic order: brown, magnoliids; green, monocots; red, rosids; blue, superasterids. Plus (+) and minus (-) symbols in the 0 column indicate *cox1 *intron presence or absence, respectively. RNA editing sites are in red in the ancestral sequence. Sites diagnostic of extended co-conversion are in pinkish-brown (Solanoideae, *Melia *and *Cynomorium*), blue (Acanthaceae), green (Musaceae), and yellow (*Brunfelsia jamaicensis*). Vertical bars at far left indicate groups of taxa inferred to have acquired their introns by the same transfer event, with subsequent vertical transmission of the intron within each marked clade, whereas all non-marked intron-containing taxa are inferred to have acquired their introns via separate transfers (Barkman et al. 2007; Sanchez-Puerta et al. 2008).

To date, no recognizable CCT has been described in the 5' exon of *cox1*, whereas a canonical CCT of minimally 3-21 bp is present in the 3' exonic region immediately downstream of the intron [9,10,12,15]. This 3' CCT is defined by between 1 and 7, highly conserved, third-position synonymous-site differences and an effectively silent difference at the C-to-U RNA editing site located at position +20 relative to the intron insertion site (Figure 5). None of the 55 sequenced intron-lacking *cox1 *genes from the Solanaceae contains any sign of a 3' CCT, whereas all 17 intron-containing genes do contain a 3' CCT motif (Figure 5). All 16 intron-containing *cox1 *genes from the Hyoscyameae and *Mandragora *clades possess all 7 nucleotide differences that are diagnostic of previously described CCTs of 20 bp in length (canonical CCT; Figure 5). Furthermore, these 16 genes share two additional differences in this region, at positions +27 and +35. This extended region of similarity probably reflects longer tracts of 3' co-conversion than any previously recognized for this intron in angiosperms. Note the perfect correspondence between the presence of A and T at positions +27 and +35, respectively, and the presence of the intron in these two clades of Solanaceae (Figure 5), i.e., all 55 sequenced intron-lacking *cox1 *genes from the Solanaceae contain the ancestral G and C at these two positions. Furthermore, the possibility of parallel substitutions at *both *positions in these two intron-containing clades is remote given the extremely high level of *cox1 *sequence conservation within the family. Apart from the 9 differences that we take to define a 3' CCT of minimum length 35 bp (Figure 5), the 744 bp of *cox1 *coding sequence determined for the two intron-containing species of *Mandragora *are identical to the intron-lacking gene from *M. caulescens *except for a single autapomorphy in the latter species (Additional File 2). Likewise, setting aside the putative 3' CCT of 35 bp and also the highly homoplasious sites -11 and +60 (Figure 5), the 723-1,362 bp of *cox1 *sequence determined for the intron-containing Hyoscyameae are identical to the ancestral sequence for the tribe (Additional File 2). Finally, the *cox1 *exons of all intron-containing *Mandragora *and Hyoscyameae are identical, again excepting the above-noted sites, to the ancestral *cox1 *sequence as reconstructed for the *entire *family Solanaceae (Additional File 2).

Discovery of 3' CCTs of unprecedented length (in the context of angiosperm *cox1 *genes) in these two lineages of intron-containing Solanaceae led us to re-examine all previously published angiosperm *cox1 *genes for potentially overlooked evidence of extended exonic co-conversion. In most cases, we saw no reason to change published estimates of the minimum length of the 3' CCT [9-12,15]. However, we did identify five additional lineages of angiosperms for which we now infer longer tracts of putative 3' co-conversion than recognized previously. Three of these lineages are each represented by a single examined species and have either an identical or further extended 3' CCT to that found in Hyoscyameae and *Mandragora*. An identical 3' CCT (of minimal length 35 bp) is found in *Cynomorium songaricum *(Cynomoriaceae, Rosales), while the other extant member of this genus of holoparasites, *C. coccineum*, shares an even longer 3' CCT (of minimal length 78 bp) with the unrelated *Melia toosendan *(Meliaceae, Sapindales) (Figure 5). In passing, we note that the latter two taxa might also share a 5' CCT extending minimally 34 or even 59 bp upstream of the intron; however, the evidence here is weak given that the diagnostic C-to-A and C-to-T sites that respectively define this potential 5' CCT are highly homoplastic across angiosperms (Figure 5; Additional File 2; and data not shown).

*Melia *and *C. coccineum *contain the *cox1 *intron, but *C. songaricum *does not. The *cox1 *introns of *Melia *and *C. coccineum *reside within the same large, essentially unresolved group of introns (bottom third of Figure 3). This group includes the introns from Hyoscyameae and *Mandragora*, which as noted, share part of the 3' CCT extension found in *Melia *and *C. coccineum*. The monophyly of introns from all four of these lineages (*Melia, C. coccineum, Mandragora*, and Hyoscyameae) is not rejected by the Approximately Unbiased test [37], and there is in fact one synapomorphy for these 4 sets of introns (Additional File 2). We therefore conclude, based essentially on CCT similarities, that these 4 sets of introns constitute a clade with respect to *cox1 *intron phylogeny. The absence of the intron from *C. songaricum *presumably reflects secondary intron loss given the striking presence in the gene of an extended 3' CCT marked by 9 diagnostic characters.

The fourth extended-CCT lineage includes both sampled members (*Musa *and *Musella*) of the Musaceae (Zingiberales). Their *cox1 *genes lack the expected monocot signatures at three clustered sites (positions +42, +45, and +63) and instead possess a core-eudicot signature T at +57, as well as T at a position (+60) that is G in all other examined monocots but T in a number of core eudicots (Figure 5). Apart from this short region, the Musaceae *cox1 *genes share all of the many monocot- or Zingiberales-specific markers that are found scattered across the rest of the gene, and, accordingly, the Musaceae genes cluster strongly with other monocot genes, and specifically with other Zingiberales genes, in *cox1 *phylogeny (Figure 4). The Musaceae *cox1 *coding sequence thus appears to be chimeric, consisting primarily of native sequence in which is embedded a small region of eudicot-derived DNA that is minimally defined by the above 5 diagnostic sites located between positions 42 and 63 of exon 2. Most likely, the Musaceae acquired the *cox1 *intron from a eudicot donor by an event involving extended 3' co-conversion that ended between positions 63 and 70 (Figure 5).

The fifth extended-CCT lineage includes all 4 sampled intron-containing members of the Acanthaceae (*Sanchezia, Justicia, Barleria*, and *Thunbergia*), which share derived changes at positions +30, +35, and +54 (Figure 5). These are the only *cox1 *exonic synapomorphies for the family other than the acquisition of the intron together with its associated canonical CCT of 21 bp. Two extreme models can account for the phylogenetic co-occurrence of these 4 sets of changes in *cox1*: A) they arose by 4 independent mutations in a common ancestor of these 4 Acanthaceae, with the *only *3 point mutations on this branch happening by chance to be clustered within a 25 bp tract (in a sequenced gene-length of 1,313 bp), and with this tract happening to be located just downstream of the phylogenetically concomitant insertion (and accompanying exonic co-conversion) of the *cox1 *intron, or B) all these changes arose by the same event in an Acanthaceae common ancestor, an event involving the insertion of a *cox1 *intron accompanied by 3' co-conversion that extended at least 54 bp in length. We strongly favor the latter model, which predicts that further sampling of angiosperms will uncover a candidate donor lineage of the Acanthaceae intron, with this lineage marked by the stepwise point-mutational accumulation of those 3 nucleotides that define the putative 3'-extended-CCT in Acanthaceae.

Finally, there is weak evidence that the newly reported *cox1 *gene of *Brunfelsia jamaicensis *may also possess an extended 3' CCT. The evidence here derives from essentially a single position, +81, at which this species has reverted from T to C relative to all 74 other examined species from the Solanaceae, including 6 other *Brunfelsia *species (the +60 site also marked in *B*. *jamaicensis *in Figure 5 carries little diagnostic weight owing to its extensive homoplasy within the family).

## Discussion

### Three intron acquisitions during Solanaceae evolution: further evidence for the rampant-transfer model of *cox1 *intron evolution and for phylogenetically local HGT

The *cox1 *intron is present in three distantly related lineages of Solanaceae, two of which belong to the large (~2,200 species) subfamily Solanoideae and one to the tribe Petunieae (Figure 2). *Brunfelsia jamaicensis*, the sole intron-containing member of the Petunieae among the species tested, possesses an intron that is radically different from those found in Solanoideae in overall sequence (Figure 4 and Additional File 2), in associated CCT sequence (Figure 5), and in phylogenetic position (Figure 3). Phylogenetic analysis of the intron resolves the three intron-containing Solanaceae lineages into two separate clades, suggesting multiple independent origins of the intron in Solanaceae (Figure 3). Furthermore, a single origin of all three clades of Solanaceae introns is strongly rejected (P = 0.00002) by the AU test. Ignoring the intron's disjunct distribution within Solanoideae (see below), such an origin would also require a bare minimum (note the two major relevant polychotomies in Solanaceae phylogeny; Figure 2) of five independent losses of the intron elsewhere in the family, each concomitant with loss of the entire suite of CCT-diagnostic characters. As explained below, such loss would require extraordinary, if not entirely implausible, circumstances. Given all this, it is clear that *B. jamaicensis *acquired its intron independently of the intron-containing members of the Solanoideae.

**Figure 6:**
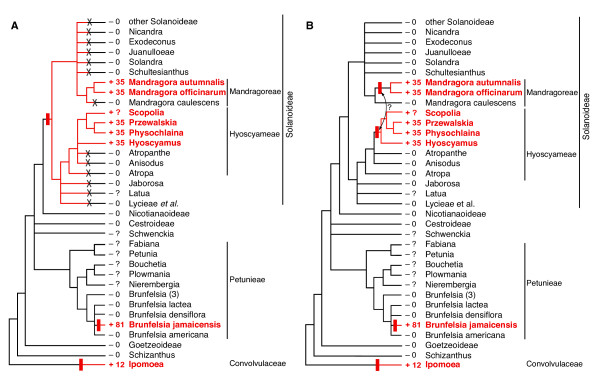
**Alternative extreme models of *cox1 *intron evolution in Solanoideae**. Presence of the intron is indicated by red branches, names in red boldface, and red plus (+) symbols. Black lines and minus (-) symbols show intron-lacking taxa. Numbers to the left of plant names give the minimum estimated size of the 3' CCT (question marks indicate that exons were not sequenced). Intron gain is marked by a filled red rectangle, while intron *and *CCT loss are marked by a black "X". A) Single intron gain in Solanoideae, followed by multiple losses of the intron and CCT. B) Two intron gains in Solanoideae, with no intron or CCT losses. The double-headed arrow indicates a possible within-Solanaceae horizontal transfer of the intron.

The situation within the Solanoideae is very different, as its two, relatively distantly related lineages of intron-containing taxa contain highly similar introns, possess identical and distinctive CCTs, and their introns form a strongly supported monophyletic group (Figures 3 and 5; Additional File 2). At the extreme, two models of intron gain and loss can account for these data: A) the intron was acquired once, at the base of the subfamily, followed by between 5 and 13 losses, the exact number of which depends on the resolution of three relevant polychotomies within the group (Figures 2 and 6A), or B) *Mandragora *and a clade within tribe Hyoscyameae acquired the intron independently, with no intron losses in the subfamily (Figure 6B).

We strongly favor the latter model. First, consider the probability of loss versus gain of the *cox1 *intron. Intron loss is in general a rare event in angiosperm mitochondrial genomes, including the Solanaceae [38] (Qiu, Y.L., N. Kubo & J.D. Palmer, unpublished), and so this would represent an exceptional amount of intron loss, especially at this phylogenetic level. Moreover, the *cox1 *intron should be less prone to loss than other introns because it alone among angiosperm mitochondrial introns contains a homing endonuclease-like ORF, whose predicted activity should cause intron-lacking *cox1 *alleles that arise by the occasional retroprocessing event to be re-colonized by the intron before they can go to fixation [note that because the intron ORF is nearly identical across all intron-containing Solanoideae, its sequence is essentially the same as that of the ORF upon arrival (i.e., homing), at which point the endonuclease must have been functional]. Finally, with respect to the probability of intron gain, the *cox1 *intron is so clearly a highly mobile intron that to postulate one additional horizontal transfer (two gains within Solanoideae rather than one), when more than 80 such events have already been documented [8-12], hardly stretches the bounds of imagination.

Second, moving beyond the intron *per se*, consider the exonic regions immediately flanking the *cox1 *intron. All 16 Solanoideae that possess the intron contain an identical, extended 3' CCT marked by 9 diagnostic characters, whereas all 30 sequenced subfamily members that lack the intron also lack all 9 characters, featuring instead the ancestral state at all 9 sites (Figure 5). Thus the intron-loss model must account not only for multiple intron losses, but also for the phylogenetically concomitant "reversal" at all 9 CCT sites in each and every case of intron loss. Reversal by point mutation is inconceivable, considering A) how many sites are involved, B) that all these changes would have occurred *en masse *in the same 5-13 lineages without even a single CCT point mutation occurring elsewhere in the subfamily, and C) that *cox1 *is otherwise virtually identical across the Solanoideae (Figure 4 and Additional File 2). Reversal by gene conversion, e.g., by an ancestral-like *cox1 *sequence present elsewhere in the mitochondrial genome, is a more reasonable possibility, as this would require but a single event in each intron-loss lineage rather than 9 parallel point mutations. Also, gene conversion seems to be relatively common in plant mitochondrial genomes (e.g. [39-41]).

To explain the strict co-occurrence of putative intron loss and gene conversion is, however, more challenging. The only plausible mechanistic link between these two events requires an additional mitochondrial copy of *cox1 *that A) lacked the intron, B) lacked the CCT, and C) was retained in many descendant lineages throughout at minimum the first 15 million years of early Solanoideae diversification [42] (see next section for discussion of an implausible but proposed mechanism). Under this scenario, the intron-lacking copy would have either converted the intron-containing copy - on 5-13 different occasions - by an event that led to simultaneous loss of both the intron and CCT or else functionally replaced the intron-containing copy, thus allowing it to be lost. The challenge here is that, as elaborated above, an intron-lacking copy of the gene is unlikely to have persisted as such - in the same genetic compartment, much less for so long, and in so many lineages - in the presence of a likely functional intron-encoded endonuclease. Only if the conversion donor/replacement copy of *cox1 *were protected from homing-mediated intron spread by being sheltered in another compartment or organism should it persist in an intron-less state. "Another compartment" basically means the nucleus (which, unlike the chloroplast, typically contains many mitochondrial sequences; [43]), but a nuclear location is problematic on two counts: A) the odds of multiple nuclear-to-mitochondrial transfers of an identical, one-in-a-million nuclear sequence, each followed by gene conversion or replacement, are slim, and B) a nuclear location of the converting sequence is incompatible with the mitochondrial-like conservation of *cox1 *in the Solanoideae (Figures 3 and 4 and Additional File 2) given that synonymous substitution rates are generally about 20 times higher in the nucleus than the mitochondrion in plants [36,44] and that a nuclear form of *cox1 *should evolve as a pseudogene. "Another organism" means horizontal gene transfer, but this is unlikely because horizontal transfer of an inert, intron- and CCT-lacking *cox1 *copy should *a priori *be less frequent than transfer of the intrinsically mobile *cox1 *intron itself, moreover each transfer would again have to be followed by gene conversion or replacement.

In summary, it is clear that *Brunfelsia jamaicensis *acquired its *cox1 *intron independently of *Mandragora *and Hyoscyameae, and it is likely that these latter two lineages acquired their introns via independent horizontal transfer events, in which case the intron has been acquired at least three times during Solanaceae evolution (Figure 6B). If so, one of the latter two transfers might have occurred from one lineage of the Solanoideae to the other because the two clades' introns are sisters in intron phylogeny and virtually identical in sequence (Figures 3 and 5, Additional File 2). An intrafamilial transfer in the Solanaceae would be consistent with evidence from other studies [9,12], which suggests that intrafamilial transfers of the *cox1 *intron in angiosperms may be relatively common compared to phylogenetically broader transfers. Illegitimate pollination or shared vectoring agents may be responsible for this pattern [6,12].

### Rejection of the ancestral-presence/rampant-loss model of *cox1 *intron evolution

In a 2008 paper that was largely a reevaluation of the results and interpretations of two earlier studies by our group [9,10], Cusimano et al. [15] reached opposite conclusions to these two studies, as well as the current study. They concluded that "the *cox1 *intron entered angiosperms once, has since largely or entirely been transmitted vertically, and has been lost numerous times, with CCT footprints providing unreliable signal of former intron presence." In an already-lengthy paper [12] that appeared shortly after Cusimano et al. [15], we had space to only briefly rebut its conclusions, which we contended then - and still contend - are based on a seriously flawed interpretation of the extensive incongruence between *cox1 *intron phylogeny and angiosperm phylogeny as well as an entirely unrealistic mechanism to account for putative "loss" of the CCT. We plan to publish a separate paper presenting a detailed rebuttal of the interpretations and conclusions of Cusimano et al. [15]. For now, we will let past studies (by our group ([9,10,12] and by others [8,11]) and, importantly, the results presented in the current study (in particular, note the strikingly incongruent intron and exon phylogenies shown in Figures 3 and 4, respectively) stand in rebuttal of Cusimano et al.'s untenable claim that phylogenetic analyses (including their own; see their Figure 4) of the *cox1 *intron "are largely congruent with known phylogenetic relationships" and that the only phylogenetic "finding suggestive of horizontal *cox1 *intron transfer" is actually poorly supported and instead best explained by vertical transmission.

We will, however, confront more explicitly the issue of CCT evolution, because it is so fundamental to interpretation of the gain/loss history of the intron in the Solanoideae. Cusimano et al.'s all-loss model of *cox1 *intron evolution postulates over 100 losses across angiosperms of the multi-character CCT, with each CCT loss accompanied by intron loss. To account for these many concomitant losses, Cusimano *et al*. [15] proposed "that the *cox1 *coconversion tract is usually lost during the intron excision process...most likely...by reverse transcription-mRNA-mediated coconversion." There is, however, no published evidence that any reverse transcriptases engage in co-conversion and, even if they did, the *cox1 *mRNAs that would mediate this putative co-conversion would still possess the CCT and therefore the CCT region would be unaffected. Furthermore, although plant mitochondrial intron loss is indeed an RNA-mediated process, known as "retroprocessing" [45,46], this would actually lead to a very different set of diagnostic changes in exonic regions immediately following the site of intron loss, namely, C-to-T substitution at intron-flanking sites of C-to-U mRNA editing. Importantly, however, this, well-grounded prediction is not met by the *cox1 *data, both across angiosperms and within the Solanaceae. For instance, the many lineages of intron-lacking Solanaceae (and almost all other intron-lacking angiosperms) contain C at the closest RNA edit site to the intron (20 bp downstream of it; Figure 5), exactly as expected if they never possessed the intron, and contrary to the T expected if these genes once had the intron, but lost it via retroprocessing. In contrast, the great majority of intron-containing taxa possess T at this site (Figure 5 and data not shown). Finally, the discovery in *Cynomorium songaricum *of a *cox1 *gene that lacks the intron but contains a full length (if not extended) CCT augments two previously reported cases of intron loss unaccompanied by CCT loss [12] and further argues against the proposal by Cusimano *et al*. [3] that retroprocessing somehow leads to both intron and CCT loss. In short, Cusimano *et al*.'s proposed model for CCT loss is both mechanistically implausible and fails to fit any of the observed *cox1 *data.

### Implications of extended co-conversion

Previous studies recognized a short (minimally 3-21 bp) 3' CCT motif, and no 5' CCT, in angiosperm *cox1 *genes that harbor the homing group I intron in question [9,10,12,15]. The current study provides the first evidence that 3' co-conversion in angiosperm *cox1 *genes sometimes extends considerably further than this, at least 35-81 bp downstream of the intron in four different intron clades, and raises the possibility that 5' co-conversion might also occur. In a sense, these results are not surprising, given experimental studies in such diverse systems as yeast mitochondria (including the cognate *cox1 *intron), *Chlamydomonas *chloroplasts, and phage T4, which have shown that CCTs are commonly hundreds and sometimes thousands of bp in length, and are often found on both sides of a newly arrived intron [16,18-20]. More surprising, therefore, is that CCTs *appear *to be so short in angiosperm *cox1 *genes. Appearances may be deceiving here: the combination of exceptionally low mutation rates in most plant mitochondrial genomes [34-36,44] and strong constraint on *cox1 *sequence evolution [47] results in such high conservation of *cox1 *sequences, even across angiosperms, that CCTs of dozens to hundreds of bp in length could easily go undetected, and probably often do.

That the great majority of intron-containing angiosperms show no evidence of 5' co-conversion and only 18-21 bp of 3' co-conversion may be largely a consequence of the crucial horizontal transfer event that first introduced this intron into angiosperms. Assuming the donor in this event was a fungus [13,14], then the great gulf of amino acid divergence between plant and fungal COX1 proteins may have selected for unusually short co-conversion, to avoid fixing an inharmoniously chimeric form of this key respiratory protein. If so, then once the intron commenced spreading rampantly from one angiosperm lineage to another, most of its co-conversions were probably longer than the short fungal co-conversion of most likely 18 or 21 bp on the 3' side, thus preserving that motif as the predominant 3' CCT among angiosperms. Under this model, the density of change within the fungal-derived 3' CCT (i.e., at all 6-7 synonymous sites), together with the polarity of co-conversion (extending from the intron insertion site outward into a flanking exon), yields an asymmetric expectation for one's ability to detect short vs. long co-conversion. Co-conversions shorter than this well-marked, 18-21-bp motif will be readily detected, hence the gradient of 3'-to-5' shortened CCTs already well recognized (Figure 5; [9,10,12]). In contrast, co-conversion beyond this motif will usually be difficult if not impossible to discern, with the various extended 3' CCTs recognized in this study representing those relatively rare cases in which the donor group happens to have accumulated enough substitutions in these regions to generate a reasonably obvious footprint.

### Solanaceae intron acquisitions: biogeography and donors

The center of diversity (and most likely the place of origin) of the Solanaceae is in the New World, with a minimum of 8 dispersal events to the Old World inferred from phylogenetic studies and overall distribution [48]. Among these events are independent dispersals of the ancestors of both *Mandragora *and Hyoscyameae, whose current distributions are restricted largely to Eurasia, with a few species found in northern Africa [42]. The intron-containing clade of *Mandragora *is restricted to the Mediterranean-Turanian region, while the intron-containing clade of Hyoscyameae has a broader distribution, with two subclades also restricted to the Mediterranean-Turanian region but other lineages found in various parts of Asia. Given this, and the very close relationship between the introns of these two clades (Figures 3 and 5), it is not unlikely that both transfer events occurred in the Mediterranean-Turanian region. The first transfer probably involved a non-Solanaceae donor, while the second may well have occurred between Hyoscyameae and *Mandragora *(see first Discussion section). If so, then there is no basis for favoring transfer in one direction vs. the other. This is because current estimates of divergence times for the two groups [42] fail to resolve the relative timing of the two horizontal transfers (Figure 6B). Overlapping geographic distributions and similarities in floral morphology between *Mandragora *and Hyoscyameae leave open the possibility that intron transfer between the two groups occurred via a shared mycorrhizal associate or pollinator, or by illegitimate pollination.

The non-Solanaceae donor of the Hyoscyameae/*Mandragora *intron type is unclear based on intron phylogeny (Figure 3). However, given the relatively long and well-supported branch leading to the Hyoscyameae/*Mandragora *intron clade, and that hundreds if not thousands of additional intron-containing clades are likely to be revealed upon sampling the > 99% of unexamined angiosperms, it is not unreasonable to expect that non-Solanaceae angiosperms with distinctly more closely related introns will be discovered. Although intron phylogeny is currently uninformative as to the Hyoscyameae/*Mandragora *intron donor, the 3' exonic CCT provides important potential clues. These Solanaceae introns share an identical extended CCT (Figure 5) with only *Melia toosendan *(Meliaceae) and also both extant species of *Cynomorium *among over 200 examined intron-containing angiosperms representing an estimated 80+ intron acquisitions. We therefore predict that any angiosperms found to contain a more closely related intron to the Hyoscyameae/*Mandragora *type will also have the same, extended CCT. The association with *Cynomorium *is intriguing, given the frequent transfer, in both directions, of mitochondrial genes between parasitic plants and their hosts [3,4,8,41,49,50]. Also, there is substantial range overlap between the intron-containing clades of Hyoscyameae and *Mandragora *and one or both species of *Cynomorium*.

*Brunfelsia *may have acquired the intron quite recently, as *B. jamaicensis *is the only one of 7 species examined in the genus found to possess it. However, in the absence of any solid estimates of phylogeny and divergence times for the genus and of comprehensive sampling of the 40-50 species in the genus, the timing and location of transfer and the phylogenetic distribution of the intron within the genus are uncertain. It will be interesting to determine the relationship of *B. jamaicensis *to the 5 other species of *Brunfelsia *endemic to the Caribbean island of Jamaica, none of them yet sampled, and whether any of them also possess the intron.

## Conclusions

Multiple lines of evidence lead us to conclude that the *cox1 *intron was acquired by horizontal transfer on at least 3 separate occasions during the evolution of the Solanaceae. One lineage of intron-containing Solanaceae may have acquired its intron from another lineage in the family, consistent with previous evidence that horizontal transfer in plants is biased towards phylogenetically local events. Discovery of these transfers was dependent on extensive sampling of the family. This underscores the importance of greatly expanded sampling of angiosperms in general in order to gain a deeper understanding of the intron's evolutionary history, including not only an accurate estimate of the number and timing of its many transfers but also to untangle to the extent possible mechanisms of transfer and donor-recipient relationships for specific transfer events.

Our findings strongly reinforce the idea that the *cox1 *intron, which encodes a homing endonuclease, is an exceptionally mobile genetic element in angiosperms. These results, together with the discovery of a rare case of likely loss of this intron accompanied by retention of the CCT, provide still further support for the long-standing, rampant-transfer model for the evolution of this intron in angiosperms [8-12] and render the rampant-loss model [15] even more implausible than already regarded.

The identification of exonic co-conversion tracks substantially longer than those previously recognized for this intron in angiosperms implies that other *cox1 *co-conversions may be longer than realized but obscured by the exceptional conservation of plant mitochondrial sequences. This is also consistent with the hypothesis that the intron's founding arrival in angiosperms, probably from a fungal donor, was aided by unusually short co-conversion, thereby minimizing the potentially deleterious effects of creating a chimeric, fungal/plant form of the key respiratory protein encoded by *cox1*. The discovery of the *cox1 *intron in 3 distinct lineages of the Solanaceae opens the door to experimental, somatic-cell genetic studies on the transmission and co-conversion properties of this intron in plants. Cybrids have been reported between tobacco, which lacks the intron, and two species in the intron-containing Hyoscyameae clade [23,51] and may well be feasible with other intron-containing Solanaceae. Somatic crosses should allow one to test whether the intron is preferentially transmitted relative to other mitochondrial loci, as expected if it does indeed encode an active homing endonuclease, and to measure the frequency and length of co-conversion.

## Methods

### Plant material and DNA extraction

Plant materials were collected by different researchers from around the world. Seeds of various species were obtained from the Nijmegen Botanical Garden. Plant DNAs were either extracted from fresh or dried leaves using a cetyl-trimethyl-ammonium-bromide DNA-extraction protocol [52] or obtained from other sources (e.g., DNA bank at the Royal Botanical Garden, Kew). Plant and DNA accession numbers are listed in Additional File 1. To rule out the possibility of DNA contamination or mistaken identity for several key intron-containing species, DNA samples from different sources were examined for these species (Additional File 1).

### Sequence Amplification

To survey the presence/absence of the group I intron in *cox1*, a PCR/gel sizing assay was performed using two primers - cox1-3 (5'-CATCTCTTTYTGTTCTTCGGT-3') and cox1-6 (5'-AGCTGGAAGTTCTCCAAAAGT-3') - that amplify most of exon 2 and a small portion of exon 1, yielding products of either 800 bp (if the intron is absent) or 1.8 kb (if the intron is present). For selected species, additional amplifications were done with primers cox1-1 (5'-AYGAMAAATCYGGTYGATGG-3') and cox1-4 (5'-ACCGRATCCAGGCAGAATGRG-3'), which amplify most of exon 1 and a small portion of exon 2, yielding products of either 750 bp or 1735 bp. Selected PCR products were sequenced using an ABI 3730 (Applied Biosystems). Sequencing primers included PCR primers and two additional primers, both located within the intron: cox1-10 (5'-TGACTACTATCAAAGTAGA-3') and cox1-8 (5'-GTAGAGTCTTATAAGGTAGT-3'). GenBank accession numbers of sequences determined in this study are listed in Additional File 1.

### Sequence and phylogenetic analyses

Sequences were aligned manually with MacClade 4.0 [53]. Editing sites were predicted using Prep-Mt [54].

Phylogenetic analyses were performed on data sets of 71 *cox1 *intron sequences and 108 *cox1 *exon sequences, all from angiosperms. GenBank accession numbers of *cox1 *sequences obtained from NCBI are listed in Additional File 3. Sites of RNA editing (33 in total, see Additional File 2) and the previously described 20-nt CCT region [12] were excluded from the *cox1 *exon character matrix. Maximum likelihood analyses of the intron and exon data sets were performed with Garli 0.951 [55] under the General Time Reversible model with parameters for invariable sites and gamma-distributed rate heterogeneity (GTR+I+Γ4; four rate categories). This substitution model was supported by hierarchical likelihood ratio tests performed using Modeltest v.3.5 [56]. Ten independent runs were conducted using either the automated stopping criterion or for up to 5,000,000 generations to ensure convergence to a similar topology and likelihood score. Five hundred bootstrap replicates were performed.

### Alternative topology test

The approximately unbiased (AU) test was used to test whether a particular intron-based topology is significantly better than a specified (constrained) alternative topology. The CONSEL package [37] was used to calculate the approximately unbiased (AU) P values for unconstrained and constrained trees. Constrained trees included: A) monophyly of the introns from Hyoscyameae, *Mandragora, Melia *and *Cynomorium*, and B) monophyly of the introns from *Brunfelsia jamaicensis*, Hyoscyameae, and *Mandragora*. The most likely tree under each constraint was determined by searching for the best tree compatible with that constraint using PAUP* [57]. The site likelihoods for this tree and for the best tree in the unconstrained analysis were exported from PAUP*, and the AU P values were calculated from these data.

## List of abbreviations

AU: approximately unbiased; CCT: co-conversion tract; HGT: horizontal gene transfer; ORF: open reading frame

## Competing interests

The authors declare that they have no competing interests.

## Authors' contributions

MVSP co-designed the study, generated some of the data, analyzed the data, designed the figures, and co-wrote the manuscript. CCA and SZ obtained most of the DNA sequences generated for the study. ET, LB and RGO provided almost all of the DNAs used in the study, performed most of the PCR survey, and commented on the manuscript. JDP co-designed the study and co-wrote the manuscript. All of the authors read and approved the final manuscript.

## Supplementary Material

Additional file 1 **List of taxa from the family Solanaceae examined in this study**. Taxonomic information, geographic origin or source (if known), collection number (voucher herbarium), and GenBank accession numbers of taxa from the family Solanaceae examined in this study. Click here for file

Additional file 2 **The *cox1 *gene alignment**. Nucleotide alignment of the *cox1 *gene (including its intron sequence) for all taxa included in the phylogenetic analysis shown in Figure 4. Sites of predicted RNA editing are in red in the reference sequence, while the putative endonuclease ORF is in green. Click here for file

Additional file 3 **Taxonomic information and GenBank accession numbers**. Taxonomic information and GenBank accession numbers of all taxa included in the analyses shown in Figures 3 and 4. Click here for file

Additional file 4 **Phylogenetic tree of *Brunfelsia *spp**. based on chloroplast data. Maximum likelihood phylogeny of 7 species of *Brunfelsia *based on analysis of chloroplast *ndhF *and *trnLF*. Numbers above branches are bootstrap support values > 50%. GenBank numbers for sequences generated here are shown in boldface. Primers used for sequence amplification are from Olmstead *et al *[46]. Click here for file
