# Supplementary material for: Multiple recent horizontal transfers of the cox1 intron in Solanaceae and extended co-conversion of flanking exons
Source: BMC Evol Biol. 2011 Sep 27;11:277. doi: 10.1186/1471-2148-11-277 (PMC3192709; doi:10.1186/1471-2148-11-277)
Supplement: Additional file 1 — List of taxa from the family Solanaceae examined in this study. Taxonomic information, geographic origin or source (if known), collection number (voucher herbarium), and GenBank accession numbers of taxa from the family Solanaceae examined in this study. [file 1471-2148-11-277-S1.PDF]

Additional File 1. List of taxa from the family Solanaceae examined in this study

Taxon name	Source (including seed accession number for cultivated taxa); DNA accession #, if known	Herbarium voucher (collector, #, herb. acronym)	GenBank accession number[a]	Intron?
Acnistus arborescens (L.) Schltdl.	Costa Rica	L. Bohs 2577 (UT)		no
Acnistus sp.	BIRM; RGO102	Lester S.1908/81 (BIRM)		no
Anisodus luridus Link (= Scopolia lurida (Link)Dunal)	BIRM; RGO194	Lester S.0215/71 (BIRM)		no
Anisodus tanguticus (Maxim.) Pascher	RGO96-034	No voucher		no
Anisodus tanguticus (Maxim.) Pascher	China; RGO2003-083b	No voucher	JF966349	no
Anthocercis angustifolia F. Muell.	Australia; RGO94-152	R.G. Olmstead 94-05 (WTU)		no
Anthocercis gracilis Benth.	Australia; RGO99-041	H. Stace s.n. (herb. unknown)	JF966301	no
Anthocercis ilicifolia Hook.	Australia; RGO99-035	H. Stace s.n. (UWA)		no
Anthocercis intricata F. Muell.	Australia; RGO99-032	H. Stace s.n. (KPBG)		no
Anthocercis littorea Labill.	Australia; RGO99-039	H. Stace s.n. (herb. unknown)		no
Anthocercis sylvicola T.D. Macfarl. & Ward.-Johnson	Australia; RGO99-031	T. Middleton s.n. (PERTH)		no
Anthotroche blackii F. Muell.	Australia; RGO99-040	H. Stace s.n. (KPBG)		no
Anthotroche myoporoides C.A. Gardner	Australia; RGO99-038	H. Stace s.n. (KPBG)		no
Anthotroche pannosa Endl.	Australia; RGO99-034	H. Stace s.n. (KPBG)	JF966353	no
Anthotroche walcottii F. Muell.	Australia; R.G. Olmstead 96-001	D.R. and B. Bellairs 2035 (PERTH)		no
Athenaea sp.	Cultivated, USA; LB91	No voucher		no
Atropa acuminata Royle ex Lindl.	RGO2004-058	Valencia J. B. #101-04; no voucher		no
Atropa belladonna L.	RGO 96-20	UW Medicinal Herb Gard.; no voucher	JF966302	no
Atropa belladonna L.	BIRM; RGO214	Lester S.0078/70 (BIRM)	JF966351	no
Atropa caucasica Kreyer	BIRM; RGO232	Lester S.0551/69 (BIRM)	JF966352	no
Atropanthe sinensis (Hemsl.) Pascher	China; RGO2003-087	Collector unknown; coll'd 1907-1909 (GH)	JF966350	no
Atropanthe sinensis (Hemsl.) Pascher	China; RGO2003-096	Collector unknown; coll'd 1935 (GH)		no
Atropanthe sinensis (Hemsl.) Pascher	NIJ #944750119	No voucher	JF966347	no
Aureliana fasciculata (Vell.) Sendtn.	Brazil; RGO389	K. Brown s.n. (UEC)	JF966303	no
Benthamiella skottsbergii A. Soriano	Argentina; RGO2000-208	Leuenberger & Arroyo 3711 (CORD)		no
Bouchetia erecta DC. ex Dunal	RGO92-253	D'Arcy 18213 (MO)	JF966304	no
Brachistus stramoniifolius (Kunth) Miers	Guatemala; MKW H43	L. Williams 45124 (DUKE)		no
Brachistus stramoniifolius (Kunth) Miers	RGO96-085	Mario Sousa-Pena 738a (CONN)	JF966305	no
Browallia americana L.	Costa Rica	L. Bohs 2374 (UT)		no
Browallia americana L.	BIRM; RGO210	Lester S.0138/68 (BIRM)		no
Browallia eludens Van Devender & P.D. Jenkins	Arizona; RGO94-070	P. Jenkins 90-106 (ARIZ, WTU)		no
Brugmansia aurea Lagerh.	NIJ #904750282; LB138	No voucher		no
Brugmansia aurea Lagerh.	BIRM; RGO384	Lester S.0412 (BIRM)		no
Brugmansia candida Pers.	NIJ #214750020; LB139	No voucher		no
Brugmansia candida Pers.	BIRM; RGO98	Lester S.1873 (BIRM)		no
Brugmansia sanguinea (Ruiz & Pav.) D. Don	Colombia; RGO349	R.G. Olmstead s.n. (WTU)	JF966306	no
Brugmansia suaveolens (Willd.) Bercht. & C.Presl	Costa Rica	L. Bohs 2431 (UT)		no
Brugmansia suaveolens (Willd.) Bercht. & C.Presl	Matthaei Botanical Garden, in cult.; RGO119	No voucher		no
Brugmansia versicolor Lagerh.	NIJ #904750059; LB140	No voucher		no
Brunfelsia americana L.	Matthaei Botanical Garden, in cult.; RGO296	No voucher	JF966307	no
Brunfelsia densifolia Krug & Urb.	BIRM; RGO380	Lester S.1864/77 (BIRM)	JF966354	no
Brunfelsia grandiflora D. Don	BIRM; RGO379	Lester S.1865/76 (BIRM)	JF966308	no
Brunfelsia jamaicensis (Benth.) Griseb.	Matthaei Botanical Garden, in cult.	No voucher		yes
Brunfelsia jamaicensis (Benth.) Griseb.	Logee's Greenhouses, Danielson, CT (USA) R1117-2. Origin: Fairchild Gardens in Miami, FL.	No voucher	JF966280	yes
Brunfelsia lactea Krug & Urb.	ESV1; RGO98-015	L. Struwe and C. Specht 1143 (NY)	JF966309	no
Brunfelsia pauciflora var. calycina (Benth. ex DC.) J.A.Schr	Matthaei Botanical Garden, in cult.; RGO297	No voucher	JF966310	no
Brunfelsia uniflora D.Don	Brazil; RGO390	K. Brown s.n.(UEC)	JF966311	no
Capsicum annuum L. var. aviculare (Dierb.) D'Arcy & Eshbaugh	Panama	L. Bohs 2447 (UT)		no
Capsicum annuum var. annuum L.	Thailand	L. Bohs 2446 (UT)		no
Capsicum baccatum L.	RGO357	H. Eshbaugh SA203 (MU)		no
Capsicum baccatum L. var. pendulum (Willd.) Eshbaugh	Cultivated, USA	L. Bohs 2564 (UT)		no
Capsicum baccatum L. var. pendulum (Willd.) Eshbaugh	RGO354	H. Eshbaugh E1584 (MU)		no
Capsicum chacoense Hunz.	RGO353	H. Eshbaugh E1586-A (MU)		no
Capsicum chacoense Hunz.	Paraguay	L. Bohs 3207 (UT)		no
Capsicum coccineum (Rusby) Hunz.	Bolivia	L. Bohs 2754 (UT)		no
Capsicum eximium Hunz.	BIRM S.038/83 (BIRM)	L. Bohs 2463 (UT)		no
Capsicum lanceolatum (Greenm.) C.V.Morton & Standl.	Cultivated, USA; LB94	No voucher		no
Capsicum pubescens Ruiz & Pav.	Cultivated, USA	L. Bohs 2565 (UT)		no
Capsicum pubescens Ruiz & Pav.	RGO355	H. Eshbaugh E1616 (MU)		no
Capsicum rhomboideum (Humb., Bonpl. & Kunth) Kuntze	RGO356	C. Heiser 7518 (IND)	JF966312	no
Capsicum sp.	Bolivia	L. Bohs 2731 (UT)		no
Cestrum amictum Schltdl.	RGO391	No voucher		no
Cestrum aurantiacum Lindl.	Nicaragua; RGO2004-037	S. Grose 146 (HULE)		no
Cestrum elegans Schltdl. or newellii G.Nicholson	Colombia, cult. Jard. Bot. Bogota; RGO343	R.G. Olmstead s.n. (WTU)		no
Cestrum fasciculatum Miers	BIRM; RGO103	Lester S.0926 (T.296) (BIRM)		no
Cestrum fragile Francey	Nicaragua; RGO2004-043	S. Grose 153 (HULE)		no
Cestrum hediundinum Dunal	BIRM; RGO373	Lester S.1845/76 (BIRM)		no
Cestrum lanatum M.Martens & Galeotti	rec'd from W. D'Arcy; RGO92-267	W. Haber s.n. (MO)		no
Cestrum macrophyllum Vent.	Puerto Rico; RGO2003-023	R.G. Olmstead 2003-10 (WTU)		no
Cestrum megalophyllum Dunal	Bolivia	L. Bohs & M. Nee 2756 (UT)		no
Cestrum nocturnum L.	Matthaei Botanical Garden, in cult.; RGO122	No voucher		no
Cestrum psittacinum Stapf	BIRM; RGO374	Lester S.1679/75; (BIRM)		no
Cestrum rigidum Rusby	Bolivia	L. Bohs 2802 (UT)		no
Cestrum schlechtendalii G.Don	Brazil; RGO392	K. Brown s.n. (UEC)		no
Cestrum strigilatum Ruiz & Pav.	Bolivia	L. Bohs 2811 (UT)		no
Cestrum sp.	Bolivia	L. Bohs 2836a (UT)		no
Chamaesaracha coronopus A.Gray	Texas, USA; RGO413	Turner 15854 (TEX)	JF966355	no
Chamaesaracha sordida A.Gray	RGO92-245	R.G. Olmstead 92-245 (WTU)		no
Cuatresia cuneata (Standl.) Bohs	Costa Rica	L. Bohs 2394 (UT)		no
Cuatresia exiguiflora (D'Arcy) Hunz.	Costa Rica	L. Bohs 2454 (UT)		no
Cuatresia riparia (Kunth) Hunz.	Costa Rica	L. Bohs 2514 (UT)		no
Cyphanthera anthocercidea (F.Muell.) Haegi	RGO2000-197	Haegi 1456 (AD)	JF966313	no
Cyphanthera myosotidea (F.Muell.) Haegi	Australia; RGO2000-200	Alcock 9117 (AD)		no
Cyphanthera odgersii (F.Muell.) Haegi	RGO2000-198	Chinnock 3100 (AD)		no
Datura discolor Bernh.	Mexico	L. Bohs 2466 (UT)		no
Datura inoxia Mill.	Costa Rica	L. Bohs 2578 (UT)		no
Datura inoxia Mill.	Cultivated, RGO99-049	R.G. Olmstead 98-57 (WTU)		no
Datura leichhardtii F.Muell. ex Benth.	RGO933	D'Arcy 17759 (MO)		no
Datura metel L.	Beal Bot. Gard. (MSU); RGO434	No voucher		no
Datura meteloides Dunal	Beal Bot. Gard. (MSU); RGO435	No voucher		no
Datura stramonium L.	Michigan, USA; RGO366	R.G. Olmstead s.n.; (WTU)	JF966314	no
Datura wrightii Hort. ex Regel	USA	L. Bohs 2435 (UT)		no
Duboisia hopwoodii F.Muell.	Australia; RGO2003-086	Lepschi et al. 4438 (CANB)		no
Duboisia leichhardtii F.Muell.	Australia; RGO2000-201	Haegi 2056 (AD)		no
Duboisia myoporoides R.Br.	Australia; RGO414	Symon 14832 (AD)	JF966315	no
Dunalia australis (Griseb.) Sleumer	BIRM; RGO412	Lester S.0379 (BIRM)	JF966316	no
Dunalia australis (Griseb.) Sleumer	BIRM; RGO100	Lester S.0379/65 (BIRM)		no
Dunalia breviflora (Sendtn.) Sleumer	BIRM; RGO332	Lester S.0257 (BIRM)		no
Dunalia fasciculata (Miers) Sleumer	BIRM; RGO333	Lester S.1078 (BIRM)		no
Dunalia tubulosa (Benth.) J.F.Macbr.	BIRM; RGO334	Lester S.1094 (BIRM)		no
Dyssochroma viridiflora Miers	Brazil; RGO393	K. Brown s.n. (UEC)	JF966317	no
Exodeconus miersii (Hook.f.) D'Arcy	BIRM; RGO195	Lester S.1223.73 (BIRM)	JF966318	no
Fabiana denudata Miers	Argentina; RGO2006-042	A. Alaria s.n. (MERL)		no
Fabiana imbricata Ruiz & Pav.	Argentina; RGO2006-046	A. Alaria s.n. (MERL)		no
Fabiana nana (Speg.) S.C.Arroyo	Argentina; RGO2006-048	A. Alaria s.n. (MERL)		no
Fabiana patagonica Speg.	Argentina; RGO2006-066	A. Alaria s.n. (MERL)		no
Fabiana peckii Niederl.	Argentina; RGO2006-041	A. Alaria s.n. (MERL)		no
Goetzea elegans Wydler	Cultivated, Hawaii, USA	R.G. Olmstead 92-220 (WTU)	JF966319	no
Grabowskia boerhaviifolia Schltdl.	BIRM; RGO377	Lester S.1801/76 (BIRM)		no
Grabowskia duplicata Arn.	BIRM; RGO378	Lester S.0258 (BIRM)	JF966320	no
Grabowskia schizocalyx Dammer	Bolivia	M. Nee 51864 (NY)		no

Grabowskia sp.	RGO273	W. D'Arcy 17709 (MO)		no
Grammosolen truncatus (Ising) Haegi	Australia; RGO00-202	Canty 2429 (AD)	JF966321	no
Hyoscyamus albus L.	North Coast"; RGO96-013	H. Abdel-Migid 4 (WTU)		yes
Hyoscyamus albus L.	"Pharmacy Fac"; RGO96-012	A. Abdel-Migid 1 (WTU)		yes
Hyoscyamus albus L.	BIRM; RGO203	Lester S.1218/81 (BIRM)		yes
Hyoscyamus aureus L.	NIJ #914750063	No voucher	JF966283	yes
Hyoscyamus boveanus Asch. & Schweinf.	Sinai; RGO96-016	H. Abdel-Migid 8 (WTU)	JF966294	yes
Hyoscyamus desertorum (Asch. ex Boiss.) Tackholm	"desertorum"; RGO96-019	H. Abdel-Migid 11 (WTU)	JF966293	yes
Hyoscyamus muticus L.	"Suez Road"; RGO96-014	H. Abdel-Migid 6 (WTU)	JF966292	yes
Hyoscyamus muticus L.	NIJ #974750072	No voucher	JF966287	yes
Hyoscyamus niger L.	BIRM; RGO193	Lester S.1058/71 (BIRM)	JF966290	yes
Hyoscyamus niger L.	NIJ #954750047	No voucher		yes
Hyoscyamus niger L.	NIJ #A04750027	No voucher	JF966282	yes
Hyoscyamus niger L.	China 1993; RGO96-030	Collector unknown (GH)		yes
Hyoscyamus orthocarpus Schönb.-Tem.	Iraq 1975; RGO96-031	Collector unknown (GH)		yes
Hyoscyamus pusillus L.	Sinai; RGO96-018	H. Abdel-Migid 10 (WTU)	JF966291	yes
Hyoscyamus turcomanicus Pojark.	NIJ #904750014	No voucher	JF966286	yes
Iochroma australe Griseb.	Cultivated, Jard. Bot. Valencia #400-00, Spain; RGO2004-060	No voucher		no
Iochroma cyaneum (Lindl.) M.L.Green	Matthaei Botanical Garden, in cult.; RGO118	No voucher		no
Iochroma fuchsioides Miers	Colombia, Jard. Bot. Bogota; RGO348	R.G. Olmstead S-29 (WTU)	JF966323	no
Iochroma grandiflora Benth.	Cultivated at Missouri Bot. Gard.; LB92	No voucher		no
Iochroma sp.	Cultivated, USA	L. Bohs 2526 (UT)		no
Iochroma umbellatum (Ruiz & Pav.) D'Arcy	Cultivated, UC Berkely Bot. Gard., USA; RGO674	Hutchison et al., 6240 (UC)		no
Iochroma umbrosa (Kunth) Miers	BIRM; RGO92	Lester S.1602/75 (BIRM)		no
Jaborosa integrifolia Lam.	BIRM; RGO383	Lester S.0290/83 (BIRM)	JF966324	no
Jaborosa sativa (Miers) Hunz. & Barboza	Bolivia	L. Bohs 2838 (UT)		no
Jaborosa sativa (Miers) Hunz. & Barboza	BIRM; RGO231	Lester S.0234 (BIRM)		no
Jaborosa sp.	Argentina	M. Nee & L. Bohs 50853 (NY)		no
Jaborosa squarrosa (Miers) Hunz. & Barboza	Bolivia	M. Nee et al. 51819 (NY)		no
Jaltomata bernardelloana S.Leiva & Mione	Peru; RGO2007-101	T. Mione 640 (CCSU)		no
Jaltomata bicolor (Ruiz & Pav.) Mione	Peru; RGO2007-110	T. Mione 728 (CCSU)		no
Jaltomata lanata S.Leiva & Mione	Peru; RGO2007-115	T. Mione 741 (CCSU)		no
Jaltomata nigricolor S. Leiva & Mione	Peru; RGO2007-107	T. Mione 718 (CCSU)		no
Jaltomata oppositifolia S.Leiva & Mione	Peru; RGO2007-102	T. Mione 674 (CCSU)		no
Jaltomata paneroi Mione & S.Leiva	Peru; RGO2007-105	T. Mione 705 (CCSU)		no
Jaltomata procumbens (Cav.) J.L.Gentry	Beal Bot. Gard. (MSU), USA; RGO00-165	R.G. Olmstead S-24 (WTU)		no
Jaltomata procumbens (Cav.) J.L.Gentry	Costa Rica	L. Bohs 2566 (UT)		no
Jaltomata procumbens (Cav.) J.L.Gentry	Costa Rica; RGO2007-103	T. Mione 692 (CCSU)		no
Jaltomata procumbens (Cav.) J.L.Gentry	BIRM; RGO215	Lester S.0595/72 (BIRM)		no
Jaltomata salpoensis S.Leiva & Mione	Peru; RGO2007-114	T. Mione 738 (CCSU)		no
Jaltomata sanchez-vegae S.Leiva & Mione	Peru; RGO2007-116	T. Mione 742 (CCSU)		no
Jaltomata sinuosa (Miers) Mione	Peru; RGO2007-112	T. Mione 733 (CCSU)		no
Jaltomata umbellata (Ruiz & Pav.) Mione & M.Nee	Peru; RGO2007-111	T. Mione 730 (CCSU)		no
Jaltomata ventricosa (Baker) Mione	Peru; RGO2007-106	T. Mione 712 (CCSU)		no
Jaltomata weberbaueri (Dammer) Mione	Peru; RGO2007-109	T. Mione 725 (CCSU)		no
Jaltomata yacheri S.Leiva & Mione	Peru; RGO2007-113	T. Mione 737 (CCSU)		no
Jaltomata sp. "hummingbird pollinated"	Peru; RGO2007-118	T. Mione 758 (CCSU)		no
Jaltomata sp. "rednectar"	Peru; RGO2007-108	T. Mione 719 (CCSU)		no
Juanulloa mexicana (Schltdl.) Miers	BIRM; RGO369	Lester S.0411/69 (BIRM)	JF966325	
Juanulloa verrucosa (Rusby) Hunz. & R.Subils	Bolivia	L. Bohs 2774 (UT)		no
Juanulloa wardiana (D'Arcy) S. Knapp	RGO2000-123	Herrera 1312 (US)		no
Larnax glabra (Standl.) Sawyer = Deprea glabra (Standl.) H	Ecuador; RGO96-068	N. Sawyer 718 (CONN)		no
Latua pubiflora Baill.	NIJ #944750124, #2; RGO2000-217	No voucher		no
Leucophysalis grandiflora (Hook.) Rydb.	Michigan, USA; RGO368	R.G. Olmstead 88-16 (WTU)	JF966326	no
Leucophysalis nana (A.Gray) Averett	Nevada, USA; RGO2005-015	Bartholomew 5994 (MO)		no
Lycianthes aff. synanthera Bitter	RGO276	W. D'Arcy 14612 (MO)		no
Lycianthes amatitlanensis Bitter	Costa Rica	L. Bohs 2552 (UT)		no
Lycianthes asarifolia (Kunth & Bouché) Bitter	Bolivia	L. Bohs & M. Nee 2737 (UT)		no
Lycianthes ciliolata Bitter	BIRM; RGO375	Lester S.0595/72 (BIRM)		no
Lycianthes geminiflora Bitter	Mexico	L. Bohs 2567 (UT)		no
Lycianthes glandulosa (Ruiz & Pav.) Bitter	BIRM; RGO376	Lester S.1616/75 (BIRM)	JF966327	no
Lycianthes heteroclita Bitter	Costa Rica	L. Bohs 2376 (UT)		no
Lycianthes lenta (Cav.) Bitter	Cuba, Prov. Villa Clara; RGO97-013	R.G. Olmstead 96-92 (WTU)		no
Lycianthes lycioides Hassl.	Cultivated, Jard. Bot. Bogota; RGO345	R.G. Olmstead S-87 (WTU)		no
Lycianthes magdalenae Bitter	Colombia, Cult.; RGO352	No voucher		no
Lycianthes multiflora Bitter	Costa Rica	L. Bohs 2569 (UT)		no
Lycianthes pilifera Bitter	Cultivated, USA	L. Bohs 2525 (UT)		no
Lycianthes pringlei Bitter	Mexico	L. Bohs 2554 (UT)		no
Lycianthes pseudolycioides Bitter	Bolivia	L. Bohs 2749 (UT)		no
Lycianthes rantonnei (Carrière) Bitter	BIRM; RGO364	Lester S.1616/75 (BIRM); R.G. Olmstead S-96 (WTU)		no
Lycianthes sp. aff. repens (Spreng.) Bitter	Cultivated at Missouri Bot. Gard, USA.; rec'd from W. D'Arcy; RGO828	No voucher		no
Lycianthes sanctaeclarae (Greenm.) D'Arcy	Costa Rica	L. Bohs 2395 (UT)		no
Lycianthes shanesii (F.Muell.) A.R.Bean	Cultivated, seeds from SE Asia (by W. D'Arcy)	L. Bohs 2721 (UT)		no
Lycianthes sp. aff. tricolor Bitter	Cultivated, USA	L. Bohs 3163 (UT)		no
Lycianthes stephanocalyx Bitter	Mexico	L. Bohs 2562 (UT)		no
Lycium andersonii A.Gray	BIRM; RGO371	Lester S.1480/76 (BIRM)		no
Lycium andersonii A.Gray	USA; LB212	No voucher		no
Lycium andersonii A.Gray	USA	L. Bohs 2703 (UT)		no
Lycium australe F.Muell.	RGO415	Symon 14834		no
Lycium barbarum L.	Michigan, USA; RGO410	R.G. Olmstead S-35 (WTU)	JF966328	no
Lycium brevipes Benth.	BIRM; RGO370	Lester S.1480/76 (BIRM)		no
Lycium cestroides Schltdl.	BIRM; RGO196	Lester S.1480/76 (BIRM)		no
Lycium cf. ciliatum Schltdl.	Argentina	M. Nee & L. Bohs 50855 (NY)		no
Lycium chinense Mill.	Cultivated, Waimea Bot. Gard., Hawaii, USA; RGO92-212	R.G. Olmstead 92-212 (WTU)		no
Lycium ciliatum Schltdl.	Argentina	M. Nee & L. Bohs 50856 (NY)		no
Lycium cinereum Thunb.	South Africa; RGO99-052	R.G. Olmstead 99-24 (WTU)		no
Lycium cooperi A.Gray	USA	L. Bohs 2702 (UT)		no
Lycium pallidum Miers	New Mexico, USA; RGO2003-102	R.G. Olmstead 95-14 (WTU)		no
Lycium sandwicense A.Gray	Cultivated, Waimea Bot. Gard., Hawaii, USA; RGO92-224	R.G. Olmstead 92-224 (WTU)		no
Lycium shawii Roem. & Schult.	BIRM; RGO553	Lester S.1480/76 (BIRM)		no
Mandragora autumnalis Bertol.	DNA bank at Kew Gardens: Kew23298	No voucher	JF966297	yes
Mandragora caulescens C.B.Clarke	China, Ho; RGO96-011	Bartholomew, & Gilbert 1172 (GH)		no
Mandragora caulescens C.B.Clarke	China, Ho; RGO2003-092	Bartholomew, & Gilbert 1172 (GH)	JF966348	no
Mandragora officinalis L.	DNA bank at Kew Gardens: Kew13338	No voucher	JF966298	yes
Mandragora officinalis L.	BIRM; RGO555	Lester S.1480/76 (BIRM)	JF966295	yes
Mandragora sp.	Greece; DNA bank at Kew Gardens: Kew23330	No voucher	JF966296	yes
Margaranthus solanaceus Schltdl.	BIRM; RGO560	Lester S.1480/76 (BIRM)	JF966329	no
Markea neurantha Hemsl.	Costa Rica	L. Bohs 2490 (UT)		no
Markea panamensis Standl.	BIRM; RGO94	Lester S.1462/73 (BIRM)		no
Markea panamensis Standl.	RGO01-97	S. Knapp and J. Mallet 9164 (BM)	JF966322	no
Metternichia principis Mikan	Brazil; RGO2011-4	Williams 576 (MO)	JF966330	no
Nicandra physalodes (L.) Gaertn.	Beal Bot. Gard. (MSU), USA; RGO437	R.G. Olmstead S-38 (WTU)	JF966331	no
Nicandra physalodes (L.) Gaertn.	BIRM; RGO213	Lester S.1116/71 (BIRM)		no
Nicotiana acaulis Speg.	Verne Sisson, Oxford Tobacco Research Station, TW1, UK; RGO95-067	A. Colwell s.n. (WTU)		no
Nicotiana acuminata Hook.	BIRM; RGO318	Lester S.0372 (BIRM)		no
Nicotiana attenuata Steud.	BIRM; RGO337	Lester S.1001 (BIRM)		no
Nicotiana excelsior J.M.Black	BIRM; RGO309	Lester S.0904 (BIRM)		no
Nicotiana exigua Wheeler	BIRM; RGO308	Lester S.0701 (BIRM)		no
Nicotiana forgetiana hort. ex Hemsl.	Verne Sisson, Oxford Tobacco Research Station, TW 50, UK; RGO95-080	A. Colwell s.n. (WTU)		no
Nicotiana glauca Graham	BIRM; RGO338	Lester S.0024 (BIRM)		no
Nicotiana glauca Graham	Bolivia	L. Bohs 2827 (UT)		no
Nicotiana glutinosa L.	BIRM; RGO320	Lester S.0024 (BIRM)		no
Nicotiana goodspeedii Wheeler	BIRM; RGO316	Lester S.0024 (BIRM)		no
Nicotiana gossei Domin	BIRM; RGO319	Lester S.1003 (BIRM)		no
Nicotiana nudicaulis S.Watson	BIRM; RGO311	Lester S.0451 (BIRM)		no
Nicotiana obtusifolia M.Martens & Galeotti	USA	L. Bohs 2704 (UT)		no

Nicotiana otophora Griseb.	Bolivia	L. Bohs 2772 (UT)		no
Nicotiana palmeri A.Gray	BIRM; RGO336	Lester S.1027 (BIRM)		no
Nicotiana paniculata L.	BIRM; RGO322	Lester S.0560 (BIRM)		no
Nicotiana plumbaginifolia Viv.	Tobacco Germplasm Collection, NCSU...TW 106, PI 555548; RGO2007-082	No voucher		no
Nicotiana repanda Willd. ex Lehm.	Verne Sisson, Oxford Tobacco Research Station, TW 110; RGO95-069	A. Colwell s.n. (WTU)		no
Nicotiana sylvestris Speg.	Tobacco Germplasm Collection, NCSU...TW 136, PI555569; RGO2007-083	No voucher		no
Nicotiana tabacum L.	UW Greenhouse Pot# 1+NAA-PMMS; RGO98-032	No voucher		no
Nicotiana tomentosiformis Goodsp.	Tobacco Germplasm Collection, NCSU...TW 142, PI 555572; RGO2007-081	No voucher		no
Nicotiana velutina Wheeler	BIRM; RGO411	Lester S.1024 (BIRM)		no
Nicotiana wigandioides K.Koch & Fintelm.	Bolivia	L. Bohs 2804 (UT)		no
Nierembergia repens Ruiz & Pav.	Cultivated, Missouri Bot. Gard. #896680, USA; RGO92-270	No voucher		no
Nolana napiformis Phil.	Cultivated; RGO676	R.G. Olmstead S-59 (WTU)		no
Nolana spathulata Ruiz & Pav.	RGO233	Dillon & Dillon 3767 (F)		no
Nothocestrum latifolium A.Gray	RGO95-058	Herbst, Lamoureaux, Bishop 725 (COLO)		no
Nothocestrum longifolium A.Gray	RGO2002-146	L.W. Cuddihy and G. Drake 743 (UT)		no
Petunia atkinsiana (Sweet) D.Don ex W.H.Baxter	BIRM; RGO551	Lester S.0640/70 (BIRM)		no
Petunia axillaris (Lam.) Britton, Sterns & Poggenb.	BIRM; RGO206	Lester S.0367/68 (BIRM); R.G. Olmstead S	JF966332	no
Petunia inflata R.E.Fr.	BIRM; RGO550	Lester S.0172/67 (BIRM)		no
Petunia nyctaginiflora Juss.	BIRM; RGO552	Lester S.0172/67 (BIRM)		no
Phrodus bridgesii Miers	RGO2000-072	J.P. Simon 484 (UC/JEPS)		no
Physalis alkekengi L.	RGO277	W. D'Arcy 17707 (MO)		no
Physalis crassifolia Benth.	USA	L. Bohs 2722 (UT)		no
Physalis franchetii Mast.	BIRM; RGO381	Lester S.-XYZ (BIRM)		no
Physalis heterophylla Nees	Michigan, USA; RGO367	R.G. Olmstead 88-5 (WTU)	JF966333	no
Physalis longifolia Nutt.	Nebraska, USA; RGO93-091	R.G. Olmstead s.n. (WTU)		no
Physalis longifolia Nutt.	USA	L. Bohs 2434 (UT)		no
Physalis peruviana L.	Colombia, cultivated, Jard. Bot. Bogota; RGO347	R.G. Olmstead S-69 (WTU)		no
Physalis philadelphica Lam.	USA (cultivated)	L. Bohs 2433 (UT)		no
Physochlaina infundibularis Kuang	China; RGO2003-088	Collector unknown; coll'd 1993 (GH)	JF966285	yes
Physochlaina orientalis G.Don	BIRM; RGO216	Lester S.0125/71 (BIRM)	JF966289	yes
Physochlaina orientalis G.Don	NIJ #934750128	No voucher		yes
Physochlaina orientalis G.Don	NIJ #944750045	No voucher	JF966281	yes
Physochlaina physaloides G.Don	NIJ #924750021	No voucher	JF966288	yes
Plowmania nyctaginoides (Standl.) Hunz. & Subils	Mexico; RGO2000-134	Breedlove & Bartholomew 55920 (MO)		no
Protoschwenkia mandonii Soler.	Bolivia; RGO2000-122	M. Nee & J. Solomon 32037 (US)		no
Protoschwenkia mandonii Soler.	Bolivia; RGO01-139	M. Nee et al. 51827 (NY)	JF966334	no
Przewalskia tangutica Maxim.	China; Ho; RGO2003-090	Bartholomew, and Gilbert 505 (GH)	JF966284	yes
Przewalskia tangutica Maxim.	China; RGO96-035	collector not recorded (GH)		yes
Quincula lobata Raf.	Colorado, USA; RGO93-074	R.G. Olmstead 93-74 (WTU)	JF966335	no
Salpiglossis sinuata Ruiz & Pav.	BIRM; RGO205	Lester S.0181/69 (BIRM); R.G. Olmstead S-71 (WTU)		no
Saracha punctata Ruiz & Pav.	Cultivated, UC Berkley Bot. Gard., USA; RGO675	Plowman 4651 (UC)	JF966336	no
Saracha viscosa Schrad.	BIRM; RGO212	Lester S.0559/69 (BIRM)		no
Schizanthus grahamii Gill.	Argentina; RGO2004-199	R.G. Olmstead 2004-199 (WTU)	JF966337	no
Schizanthus pinnatus Ruiz & Pav.	BIRM; RGO202	Lester S.0224/66 (BIRM); R.G. Olmstead S	JF966338	no
Schultesianthus leucanthus (Donn. Sm.) Hunziker	Mexico; RGO2000-156	Wendt 6784 (TEX)	JF966339	no
Schultesianthus megalandrus (Dunal) Hunz.	Ecuador; RGO2000-125	Acevedo et al. 1715 (US)		no
Schwenckia americana Kunth	Cuba; RGO2000-166	R.G. Olmstead 96-109 (WTU)		no
Sclerophylax sp.	Argentina	M. Nee & L. Bohs 50857 (NY)		no
Sclerophylax sp.	Paraguay	L. Bohs 3209 (UT)		no
Scopolia japonica Maxim.	China; RGO2003-89	Akiyama s.n. (MO)		yes
Solandra brachycalyx Kuntze	Costa Rica; RGO92-271	T. Plowman & A. Gentry 2957 (MO)		no
Solandra grandiflora Sw.	Cultivated, Matthaei Bot. Gard., USA; RGO115	No voucher	JF966340	no
Solandra guttata D.Don	BIRM; RGO95	Lester S.0261 (T261) (BIRM)		no
Solanum accrescens Standl. & C.V.Morton	Costa Rica	L. Bohs 2556 (UT)		no
Solanum acerifolium Dunal	Costa Rica	L. Bohs 2714 (UT)		no
Solanum aculeatissimum Jacq.	NIJ #924750122	Cipollini 60 (UT)		no
Solanum acuminatum Ruiz & Pav.	Bolivia	M. Nee 51762 (NY)		no
Solanum adhaerens Willd. ex Roem. & Schult.	Costa Rica	L. Bohs 2473 (UT)		no
Solanum adhaerens Willd. ex Roem. & Schult.	Costa Rica	L. Bohs 2704 (UT)		no
Solanum adscendens Sendtn.	Bolivia	L. Bohs 2738 (UT)		no
Solanum aethiopicum L.	Cultivated, France	L. Bohs 3241 (UT)		no
Solanum aethiopicum L.	Madagascar	L. Bohs 2531 (UT)		no
Solanum albidum Dunal	Bolivia	M. Nee 51831 (NY)		no
Solanum aligerum Schltdl.	Bolivia	M. Nee 51822 (NY)		no
Solanum americanum Mill.	Costa Rica	L. Bohs 2400 (UT)		no
Solanum amotapense Svenson	BIRM	Lester S.0034a (BIRM); L. Bohs 2479 (UT)		no
Solanum amygdalifolium Steud.	Argentina	M. Nee & L. Bohs 50840 (NY)		no
Solanum anceps Ruiz & Pav.	Bolivia	L. Bohs 2790a (UT)		no
Solanum aphyodendron S.Knapp	Bolivia	M. Nee 51852 (NY)		no
Solanum arboreum Dunal	Costa Rica	L. Bohs 3501 (UT)		no
Solanum argentinum Bitter & Lillo	Argentina	L. Bohs 2539 (UT)		no
Solanum argentinum Bitter & Lillo	Bolivia	L. Bohs 2732 (UT)		no
Solanum aridum Morong	Bolivia	L. Bohs 2733 (UT)		no
Solanum armentalis J.L.Gentry & D'Arcy	Costa Rica	L. Bohs 2593 (UT)		no
Solanum arundo Mattei	NIJ #944750128	L. Bohs 2726 (UT)		no
Solanum atricoeruleum Bitter	Bolivia	L. Bohs 2794 (UT)		no
Solanum aturense Dunal	Costa Rica	Soto 1219 (UT)		no
Solanum aturense Dunal	Costa Rica	L. Bohs 2555 (UT)		no
Solanum aviculare G.Forst.	Australia	L. Bohs 3554 (UT)		no
Solanum bahamense L.	Cultivated, USA	L. Bohs 2575 (UT)		no
Solanum betaceum Cav.	Argentina	L. Bohs 2946 (UT)		no
Solanum betaceum Cav.	Bolivia; RGO 442	L. Bohs 2468 (UT)	JF966341	no
Solanum betaceum Cav.	Cultivated, Matthaei Bot. Gard., USA; RGO124	No voucher		no
Solanum bulbocastanum Dunal	Mexico	Tarn 153 (PTIS)		no
Solanum cacosmum Bohs	Brazil	Cid 2894 (GH)		no
Solanum cajanumense Kunth	Cultivated; RGO443	No voucher		no
Solanum calileguae Cabrera	Bolivia	L. Bohs 2834 (UT)		no
Solanum campechiense L.	Costa Rica	L. Bohs 2536 (UT)		no
Solanum candidum Lindl.	Costa Rica	L. Bohs 2898 (UT)		no
Solanum capsicastrum Schauer	Australia	L. Bohs 2535 (UT)		no
Solanum capsicoides All.	Peru	L. Bohs 2451 (UT)		no
Solanum caricaefolium Rusby	Bolivia	L. Bohs 2741 (UT)		no
Solanum carolinense L.	USA	Cipollini s.n. (UT)		no
Solanum celsum Standl. & C.V.Morton	Costa Rica	L. Bohs 2592 (UT)		no
Solanum cervantesii Lag.	BIRM	Lester S.0729/72 (BIRM); L. Bohs 2455 (UT)		no
Solanum cf. amotapense Svenson	BIRM	Lester S.0042 (BIRM); L. Bohs 2570 (UT)		no
Solanum cf. pastillum S.Knapp	Costa Rica	L. Bohs 2513 (UT)		no
Solanum cf. storkii C.V.Morton & Standl.	Costa Rica	L. Bohs 2617 (UT)		no
Solanum chalmersii S.Knapp	Bolivia	M. Nee 51777 (NY)		no
Solanum chrysotrichum Schltdl.	Costa Rica	L. Bohs 2713 (UT)		no
Solanum cinereum R.Br.	NIJ #904750120	L. Bohs 2852 (UT)		no
Solanum circinatum Bohs	Colombia; RGO447	L. Bohs 2301 (GH)		no
Solanum circinatum L. Bohs subsp. circinatum	Colombia	L. Bohs 2442 (UT)		no
Solanum clandestinum Bohs	Bolivia	M. Nee 51781 (NY)		no
Solanum complectens M.Nee & G.J.Anderson	Bolivia	L. Bohs 2452 (UT)		no
Solanum confusum C.V.Morton	Bolivia	L. Bohs 2776 (UT)		no
Solanum confusum C.V.Morton	Bolivia	L. Bohs 2780 (UT)		no
Solanum confusum C.V.Morton	Bolivia	L. Bohs 2790 (UT)		no
Solanum confusum C.V.Morton	Bolivia	L. Bohs 2836 (UT)		no
Solanum confusum C.V.Morton	Argentina	L. Bohs 2853 (UT)		no
Solanum conglobatum Dunal	Bolivia	L. Bohs 2740 (UT)		no
Solanum conicum Ruiz & Pav.	Peru	Tepe and Stern 2270 (UT)		no
Solanum cookii Symon	NIJ #904750182	L. Bohs 2851 (UT)		no
Solanum cordovense Sessé & Moc.	Costa Rica	L. Bohs 2501 (UT)		no
Solanum corumbense S.Moore	Bolivia	L. Bohs 2739 (UT)		no

Solanum corymbiflorum (Sendtn.) Bohs	Brazil; RGO444	L. Bohs 2343 (GH)		no
Solanum crassifolium Ortega	RGO365	No voucher		no
Solanum crinitipes Dunal	NIJ #34750184	L. Bohs 3264 (UT)		no
Solanum daphnophyllum Bitter	Bolivia	L. Bohs 2816 (UT)		no
Solanum daphnophyllum Bitter	Bolivia	L. Bohs 2791 (UT)		no
Solanum deflexum Greenm.	Costa Rica	L. Bohs 2715 (UT)		no
Solanum diphyllum L.	NIJ #94475017	Cipollini 29 (UT)		no
Solanum diploconos (Mart.) Bohs	Brazil; RGO445	L. Bohs 2335 (GH)		no
Solanum diploconos (Mart.) Bohs	BIRM; RGO99	Lester S.0729/72 (BIRM)		no
Solanum diversifolium Dunal	Cultivated; RGO446	L. Bohs 2341 (GH)		no
Solanum endopogon (Bitter) Bohs	French Guiana	L. Bohs 2716 (UT)		no
Solanum ensifolium Dunal	Cultivated, seeds from R. G. Olmstead	L. Bohs 2461 (UT)		no
Solanum evolvulifolium Greenm.	Costa Rica	L. Bohs 2500 (UT)		no
Solanum exiguum Bohs	Bolivia	L. Bohs 2758 (UT)		no
Solanum fiebrigii Bitter	Bolivia	L. Bohs 2784 (UT)		no
Solanum filiforme Ruiz & Pav.	Peru	Ochoa 15307 (CONN)		no
Solanum fraxinifolium Dunal	Costa Rica	L. Bohs 2558 (UT)		no
Solanum fusiforme L.B.Sm. & Downs	Argentina	Moscone and Davina 217 (CORD)		no
Solanum glaucophyllum Desf.	Argentina	L. Bohs 2530 (UT)		no
Solanum glaucophyllum Desf.	Bolivia	L. Bohs 2767 (UT)		no
Solanum glaucophyllum Desf.	Paraguay	L. Bohs 3201 (UT)		no
Solanum glutinosum Dunal	NIJ #914750129	Cipollini 43 (UT)		no
Solanum gympiense Symon	Australia	L. Bohs 3539 (UT)		no
Solanum hazenii Britton	Costa Rica	R. Moran and S. Stern 7764 (UT)		no
Solanum hazenii Britton	Costa Rica	L. Bohs 2652 (UT)		no
Solanum heiseri G.J.Anderson	Ecuador	C. Heiser 6800 (CONN)		no
Solanum herculeum Bohs	Spain	S. Knapp 1812 (BM)		no
Solanum hibernum Bohs	Bolivia	L. Bohs 2443 (UT)		no
Solanum hirtum Vahl	Costa Rica	L. Bohs 2563 (UT)		no
Solanum hoehnei C.V.Morton,	Brazil	Folli 1668 (MO)		no
Solanum huayavillense Del Vitto & Peten.	Bolivia	L. Bohs 2786 (UT)		no
Solanum incurvum Ruiz & Pav.	Peru	E.J. Tepe and S. Stern 2294 (UT)		no
Solanum interius Rydb	USA	L. Bohs 2464 (UT)		no
Solanum jamaicense Mill.	Costa Rica	L. Bohs 2482, 2573 (UT)		no
Solanum johannae Bitter	Brazil	Severo et al. s.n. (NY)		no
Solanum kwebense N.E.Br. ex C.H.Wright	NIJ #944750162	L. Bohs 2849 (UT)		no
Solanum laciniatum Aiton	New Zealand	L. Bohs 2528 (UT)		no
Solanum lanceolatum Cav.	Costa Rica	L. Bohs 2728 (UT)		no
Solanum lepidotum Dunal	Costa Rica	L. Bohs 2621 (UT)		no
Solanum linearifolium Geras. ex Symon	BIRM	Lester S.0785/70 (BIRM); L. Bohs 2456 (UT)		no
Solanum linnaeanum Hepper & P.-M.L. Jaeger	Australia	L. Bohs 2557 (UT)		no
Solanum lucani F.Muell.	NIJ #954750052	L. Bohs 2727 (UT)		no
Solanum lyratum Thunb.	BIRM	Lester S.1151/73 (BIRM); L. Bohs 2460 (UT)		no
Solanum mahoriense D'Arcy & Rakot.	Madagascar	L. Bohs 2576 (UT)		no
Solanum malletii S.Knapp	Peru	S. Stern et al. 147 (UT)		no
Solanum mapiriense Bitter	Bolivia	M. Nee and Solomon 30305 (UT)		no
Solanum maternum Bohs	Bolivia	L. Bohs 2547 (UT)		no
Solanum maternum Bohs	Bolivia	L. Bohs 2694 (UT)		no
Solanum maternum Bohs	Bolivia	L. Bohs 2775a (UT)		no
Solanum maternum Bohs	Bolivia	L. Bohs 2775b (UT)		no
Solanum mauritianum Scop.	Cultivated, USA	Cipollini 44 (UT)		no
Solanum melissarum Bohs	Brazil	L. Bohs 2476 (UT)		no
Solanum montanum L.	NIJ #904750205	L. Bohs 2870 (UT)		no
Solanum multifidum Lam.	NIJ #904750205	L. Bohs 2870 (UT)		no
Solanum muricatum Aiton	Colombia	R.G.Olmstead S-93 (WTU)		no
Solanum myoxotrichum Baker	Madagascar	L. Bohs 2692 (UT)		no
Solanum myriacanthum Dunal	NIJ #814750043	Cipollini 83 (UT)		no
Solanum nigrescens M.Martens & Galeotti	Costa Rica	L. Bohs 2559 (UT)		no
Solanum nigrescens M.Martens & Galeotti	NIJ #944750077	L. Bohs 2861 (UT)		no
Solanum nigrum L.	Australia	L. Bohs 2534 (UT)		no
Solanum nigrum L.	Sicily	L. Bohs 2698 (UT)		no
Solanum nitidum Ruiz & Pav.	Bolivia	M. Nee 31944 (F)		no
Solanum nitidum Ruiz & Pav.	Costa Rica	L. Bohs 2505 (UT)		no
Solanum nutans Ruiz & Pav.	Bolivia	L. Bohs 2831 (UT)		no
Solanum nutans Ruiz & Pav.	Bolivia	L. Bohs 2778 (UT)		no
Solanum palinacanthum Dunal	Bolivia	L. Bohs 2872 (UT)		no
Solanum palitans C.V.Morton	Bolivia	L. Bohs 2796 (UT)		no
Solanum palitans C.V.Morton	BIRM	Lester S.0837/70 (BIRM); L. Bohs 2449 (UT)		no
Solanum phaseoloides Polak.	Costa Rica	L. Bohs 2485 (UT)		no
Solanum phaseoloides Polak.	Costa Rica	L. Bohs 2485 (UT)		no
Solanum physalifolium Rusby var. nitidibaccatum (Bitter) Edmond	USA	L. Bohs 2467 (UT)		no
Solanum pinnatum Cav.	Argentina	L. Bohs 2954 (UT)		no
Solanum poinsettiifolium Rusby	Bolivia	D. McClelland & S. Stern 414 (NY)		no
Solanum prinophyllum Dunal	NIJ #904750171	L. Bohs 2725 (UT)		no
Solanum pseudocapsicum L.	Bolivia	L. Bohs 2812 (UT)		no
Solanum pseudocapsicum L.	Argentina	L. Bohs 2524 (UT)		no
Solanum pungetium R.Br.	NIJ #844750002	L. Bohs 2724 (UT)		no
Solanum quitoense Lam.	Ecuador	C. Heiser s.n. (UT)		no
Solanum riparium Pers.	Bolivia	L. Bohs 2734 (UT)		no
Solanum roseum Bohs	Bolivia; RGO441	L. Bohs 2338 (GH)		no
Solanum rostratum Dunal	USA; LB83	No voucher		no
Solanum rovirosanum Donn.Sm.	Costa Rica	L. Bohs 2520 (UT)		no
Solanum rudepannum Dunal	Costa Rica	L. Bohs 2579 (UT)		no
Solanum rudepannum Dunal	Costa Rica	L. Bohs 2712 (UT)		no
Solanum rugosum Dunal	Costa Rica	L. Bohs 3011 (UT)		no
Solanum scabrum Mill.	NIJ #884750172	L. Bohs 2729 (UT)		no
Solanum schimperianum Hochst. ex A.Rich.	NIJ #904750127	L. Bohs 2856 (UT)		no
Solanum sciadostylis (Sendtn.) Bohs	Brazil	L. Bohs 2453 (UT)		no
Solanum sect. Anarhichomenum	Ecuador	L. Bohs 3411 (UT)		no
Solanum sect. Geminata	Costa Rica	L. Bohs 2506 (UT)		no
Solanum sect. Geminata	Costa Rica	L. Bohs 2496 (UT)		no
Solanum sect. Geminata	Costa Rica	L. Bohs 2572 (UT)		no
Solanum sect. Petota	Costa Rica	L. Bohs 2561 (UT)		no
Solanum sect. solanum	Bolivia	L. Bohs 2794 (UT)		no
Solanum sect. solanum	Bolivia	L. Bohs 2798 (UT)		no
Solanum sessile Ruiz & Pav.	Bolivia	L. Bohs 2751 (UT)		no
Solanum sibundoyense (Bohs) Bohs	Colombia; RGO448	L. Bohs & Juajibioy 2222 (GH)		no
Solanum sisymbriifolium Lam.	Argentina	L. Bohs 2533 (UT)		no
Solanum sotobosquense Bohs	Costa Rica	Soto 1226 (UT)		no
Solanum sp.	Bolivia	L. Bohs 2835 (UT)		no
Solanum sp.	Bolivia	L. Bohs 2832 (UT)		no
Solanum sp.	Bolivia	L. Bohs 2785 (UT)		no
Solanum sp.	Costa Rica	L. Bohs 2518 (UT)		no
Solanum sp.	Bolivia	L. Bohs 2546 (UT)		no
Solanum sp.	Bolivia	L. Bohs 2548 (UT)		no
Solanum sp.	Bolivia	L. Bohs 2537 (UT)		no
Solanum sp.	Colombia	L. Bohs 2717 (UT)		no
Solanum sp.	NIJ #914750032	L. Bohs 2858 (UT)		no
Solanum sp.	NIJ #944750178; LB365	No voucher		no
Solanum sp. "spiny #1"	Cultivated	L. Bohs 2477 (UT)		no
Solanum sp. "spiny #2"	Cultivated	L. Bohs 2478 (UT)		no
Solanum sp. "Thai eggplant"	Cultivated	L. Bohs 2699 (UT)		no
Solanum sp. aff. violaceum (rec'd as Lycianthes/Solanum v	BIRM; RGO387	Lester S.2142/81 (BIRM)		no
Solanum stenophyllidium Bitter	NIJ #904750079	L. Bohs 2855 (UT)		no
Solanum stenopterum A.R.Bean,	Australia	L. Bohs 3529 (UT)		no
Solanum stuckertii Bitter	Argentina	L. Bohs 2522 (UT)		no
Solanum stuckertii Bitter	Argentina	L. Bohs 2523 (UT)		no
Solanum stuckertii Bitter	Argentina	L. Bohs 2857 (UT)		no

Solanum suaveolens Kunth & C.D.Bouché	Peru	G. Anderson 40-9 (CONN)		no
Solanum symoni H.Eichler	Cultivated, (BIRM seed collection); LB 103	No voucher		no
Solanum tabanoense Correll	Colombia	G. Anderson 768 (CONN)		no
Solanum taeniotrichum Correll	Costa Rica	G. Anderson 1423 (CONN)		no
Solanum tenuispinum Rusby	Bolivia	L. Bohs 2475 (UT)		no
Solanum terminale Forssk.	Cultivated	L. Bohs 2719 (UT)		no
Solanum toliaraea D'Arcy & Rakot.	Madagascar	L. Bohs 2574 (UT)		no
Solanum triflorum Nutt.	USA	L. Bohs 2891 (UT)		no
Solanum tripartitum Dunal	BIRM	Lester S.0708/71 (BIRM); L. Bohs 2465 (UT)		no
Solanum triquetrum Cav.	Cultivated, USA	L. Bohs 2540 (UT)		no
Solanum trisectum Dunal	France	L. Bohs 2718 (UT)		no
Solanum trizygum Bitter	Costa Rica	L. Bohs 2412 (UT)		no
Solanum tucumanense Griseb.	Cultivated, seeds from Australia	L. Bohs 2538 (UT)		no
Solanum uleanum Bitter	Cultivated	L. Bohs 2720 (UT)		no
Solanum umbellatum Mill.	Costa Rica	L. Bohs 2560 (UT)		no
Solanum uncinellum Lindl.	Bolivia	L. Bohs 2766 (UT)		no
Solanum unilobum (Rusby) Bohs	Bolivia	L. Bohs 2549 (UT)		no
Solanum unilobum (Rusby) Bohs	from Lynn Bohs, 1988; RGO449	No voucher		no
Solanum vaccinioides Schltr.	New Caledonia	L. Bohs 3608 (UT)		no
Solanum viarum Dunal	Israel	L. Bohs 2571 (UT)		no
Solanum villosum Mill.	Iran	L. Bohs 2553 (UT)		no
Solanum wallacei (A.Gray) Parish	Cultivated, Rancho Santa Ana Botanic Garden, USA	L. Bohs 2438 (UT)		no
Solanum wendlandii Hook.f.	BIRM; LB76	Lester S.0488 (BIRM)		no
Solanum wrightii Behth.	Costa Rica	L. Bohs 2445 (UT)		no
Solanum xanti A.Gray	USA	L. Bohs 2469 (UT)		no
Streptosolen jamesonii Miers	Colombia, cult. Jard. Bot. Bogota; RGO342	R.G. Olmstead S-106 (WTU)	JF966342	no
Tubocapsicum anomalum Makino	Cultivated, Missouri Bot. Gard, USA; RGO275	Chen 231 (MO)	JF966343	no
Vassobia breviflora (Sendtn.) Hunz.	Bolivia	L. Bohs 2474 (UT)		no
Vestia foetida Hoffmanns.	BIRM; RGO209	Lester S.0105 (BIRM)	JF966344	no
Withania coagulans Dunal	BIRM; RGO211	Lester S.0105 (BIRM); R.G. Olmstead S-10	JF966345	no
Witheringia asterotricha (Standl.) Hunz.	Costa Rica	L. Bohs 2377 (UT)		no
Witheringia asterotricha (Standl.) Hunz.	Costa Rica	L. Bohs 3007 (UT)		no
Witheringia fuscoviolacea (Cufod.) Hunz.	Costa Rica	L. Bohs 2568 (UT)		no
Witheringia macrantha (Standl. & C.V.Morton) Hunz.	Costa Rica	L. Bohs 2512 (UT)		no
Witheringia maculata (C.V.Morton & Standl.) Hunz.	Costa Rica	L. Bohs 2487 (UT)		no
Witheringia meiantha (Donn. Sm.) Hunz	Costa Rica	L. Bohs 3015 (UT)		no
Witheringia meiantha (Donn.Sm.) Hunz.	Costa Rica	L. Bohs 2387 (UT)		no
Witheringia mexicana (B.L.Rob.) Hunz.	BIRM; RGO386	Lester S.1199 (BIRM)	JF966346	no
Witheringia mortonii Hunz.	Costa Rica	L. Bohs 2594 (UT)		no
Witheringia mortonii Hunz.	Costa Rica	L. Bohs 2594 (UT)		no
Witheringia riparia Kunth	Costa Rica	L. Bohs 2514 (UT)		no
Witheringia solanacea L'Hér.	Costa Rica	L. Bohs 2416 (UT)		no
Witheringia solanacea L'Hér.	Costa Rica	L. Bohs 2543 (UT)		no
Witheringia sp. nov.	Costa Rica	L. Bohs 2685 (UT)		no

a- GenBank accession numbers are given for those taxa for which cox1 sequences are available.
Abbreviations:
BIRM samples have the seed accession number for the Solanaceae collection at the University of Birmingham, UK.
ESV= Eugenio Santiago-Valentin
Kew= Royal Botanical Garden, Kew
LB= Lynn Bohs
MKW=M. K. Whitson
MVSP= M.V. Sanchez-Puerta
NIJ accession numbers refer to the Solanaceae collection at Radboud University, Nijmegen, The Netherlands.
RGO= Richard G. Olmstead DNA collection
