# Supplementary material for: Multiple recent horizontal transfers of the cox1 intron in Solanaceae and extended co-conversion of flanking exons
Source: BMC Evol Biol. 2011 Sep 27;11:277. doi: 10.1186/1471-2148-11-277 (PMC3192709; doi:10.1186/1471-2148-11-277)
Supplement: Additional file 2 — The cox1 gene alignment. Nucleotide alignment of the cox1 gene (including its intron sequence) for all taxa included in the phylogenetic analysis shown in Figure 4. Sites of predicted RNA editing are in red in the reference sequence, while the putative endonuclease ORF is in green. [file 1471-2148-11-277-S2.PDF]

```
                   1          11         21         31         41         51         61         71         81         91
                   |          |          |          |          |          |          |          |          |          |
Rhamnus            ---------- ---------- ---------- ---------- ---------- ---------- ---------- ---------- ---------- ---------T
Asimina            .......... .......... .......... ....CAGTAC TGATTCGTAT GGAATTAGCA CGACCCGGCG ATCAAATTCT TGGTGGGAAT CATCAACTT.
Magnolia           GGTGCCATTG CTGGAGTGAT GGGCACATGC TTCTCAGTAC TGATTCGTAT GGAATTAGCA CGACCCGGCG ATCAAATTCT TGGTGGGAAT CATCAACTT.
Knema              .......... .......... .......... .......... .......... .......... .......... .......... .......... ..........
Myristica          .......... .......... .......... .......... .......... .......... .......... .......... .......... ..........
Peperomia          .......... .......... .......... .......... .......... .......... .......... .......... .......... .........-
Piper              .......... .......... .......... ....CAGTAC TGATTCGTAT GGAATTAGCA CGACCCGGCG ATCAAATTCT TGGTGGGAAT CATCAACTT.
Arisaema           .......... .......... .......... .......... .......... .......... .......... ..CAAATTCT TGGTGGGAAT CATCAACTT.
Zamioculcas        .......... .......... .......... .......... .......... .......... .......... ..CAAATTCC TGGTGGGAAT CATCAACTT.
Xanthosoma         .......... .......... .......... .......... .......... .......... .......... .......... .......... ..........
Philodendron       .......... .......... .......... .......... .......... .......... .......... .......... .......... ..........
Peltandra          .......... .......... .......... .......... .......... .......... .......... ATCAAATTCT TGGTGGGAAT CATCAACTT.
Strelitzia         GGTGCCATTG CTGGAGTGAT GGGCACATGC TTCTCCGTAC TGATTCGTAT GGAATTAGCC CGACCCGGCG ATCAAATTCT TGGTGGGAAT CATCAACTT.
Musa               .......... .......... .......... .......... .......... .......... .......... .......... .......... ......CTT.
Musella            .......... .......... .......... .......... .......... .......... .......... .......... .......... .........-
Maranta            .......... .......... .......... .......... .......... .......... .......... .......... .......... ..........
Monotagma          .......... .......... .......... .......... .......... .......... .......... .......... .......... .........-
Haumania           .......... .......... .......... .......... .......... .......... .......... .......... .......... .........-
Globba             .......... .......... .......... .......... .......... .......... .......... .......... .......... .........-
Costus             GGTGCCATTG CTGGAGTGAT GGGCACATGC TTCTCAGTAC TGATTCGTAT GGAATTAGCC CGACCCGGCG ATCAAATTCT TGGTGGGAAT CATCAACTT.
Triticum           GGTGCCATTG CAGGAGTGAT GGGCACATGC TTCTCCGTAC TGATTCGTAT GGAATTAGCC CGACCCGGCG ATCAAATTCT TGGTGGGAAT CATCAACTT.
Grevillea          .......... .......... .......... ....CAGTAC TGATTCGTAT GGAATTAGCA CGACCCGGCG ATCAAATTCT TGGTGGGAAT CATCAACTG.
Melia              .......... .......... .......... .......... .......... .......... .......... .......... .......... ..........
Dysoxylum          GGTGCCATTG CTGGAGTGAT GGGCACATGC TTCTCAGTAC TGATTCGTAT GGAATTAGCA CGCCCCGGCG ATCAAATTCT TGGTGGGAAT CATCAACTT.
Ailanthus          .......... .......... .......... ....CAGTAC TGATTCGTAT GGAATTAGCA CGCCCCGGCG ATCAAATTCT TGGTGGGAAT CATCAACTT.
Toxicodendron        .......... .......... .......... .......... .......... .......... .......... .......... .......... .........-
Rhus               .......... .......... .......... ....CAGTAC TGATTCGTAT GGAATTAGCA CGACCCGGCG ATCAAATTCT TGGTGGGAAT CATCAACTT.
Bur.sp             .......... .......... .......... .......... .......... .......... .......... .......... .......... ..........
Bur.simarouba      .......... .......... .......... ....CAGTAC TGATTCGTAT GGAATTAGCA CGACCCGGCG ATCAAATTCT TGGTGGGAAT CATCAACTT.
Breynia            .......... .......... .......... .......... .......... .......... .......... .......... .......... ......CTT.
Phyllanthus        GGTGCCATTG CTGGAGTGAT GGGCACATGC TTCTCAGTAC TGATTCGTAT GGAATTAGCA CTCCCCGGCG ATCAAATTCT TGGTGGGAAT CATCAACTT.
Hevea              .......... .......... .......... .......... .......... .......... .......... .......... .......... ..........
Acalypha           .......... .......... .......... .......... .......... .......... .......... .......... .......... ......CTT.
Croton             .......... .......... .......... .......... .......... .......... .......... .......... .......... ......CTT.
Euphorbia          .......... .......... .......... .......... .......... .......... .......... .......... .......... ..........
Hura               .......... .......... .......... .......... .......... .......... .......... .......... .......... ......CTT.
Malpighia          .......... .......... .......... .......... .......... .......... .......... .......... .......... ..........
Polygala           .......... .......... .......... ....CAGTGC TAATTCGTAT GGAATTAGCA CGACCCGGCG ATCAAATTCT TGGTGGGAAT CATCAACTT.
Humulus            .......... .......... .......... ....CAGTAC TGATTCGTAT GGAATTAGCA CGACCCGGCG ATCAAATTCT TGGTGGGAAT CATCAACTT.
Pilea              .......... .......... .......... .......... .......... .......... .......... .......... .......... ......CTT.
Hovenia            .......... .......... .......... .......... .......... .......... .......... .......... .......... ......CTT.
Cyn.songaricum     .......... .......... .......... ....CAGTAC TGATTCGTAT GGAATTAGCA CGACCCGGCG ATCAAATTCT TGGTGGGAAT CATCAACTT.
Cyn.coccineum      .......... .......... .......... ....CCGTAC TGATTCGTAT GGAATTAGCA CGACCCGGCG ATCAAATTCT TGGTGGGAAT CATCAACTT.
Citrullus          GGTGCCATTG CTGGAGTGAT GGGCACATGC TTCTCAGTAC TGATTCGTAT GGAATTAGCA CGACCCGGCG ATCAAATTCT TGGTGGGAAT CATCAACTT.
Melothria          GGTGCCATTG CTGGAGTGAT GGGCACATGC TTCTCAGTAC TGATTCGTAT GGAATTAGCA CGACCCGGCG ATCAAATTCT TGGTGGGAAT CATCAACTT.
Cucurbita          GGTGCCATTG CAGGAGTGAT GGGCACATGC TTCTCAGTAC TGATTCGTAT GGAATTAGCA CGACCCGGCG ATCAAATTCT TGGTGGGAAT CATCAACTT.
Lepionurus         .......... .......... .......... .......... .......... .......... .......... .......... .......... ..........
Andromeda          .......... .......... .......... .......... .......... .......... .......... .......... .......... .........-
Pyrola             .......... .......... .......... .......... .......... .......... .......... .......... .......... ......CTT.
Symplocus          .......... .......... .......... .......... .......... .......... .......... .......... .......... ..........
Diospyros          .......... .......... .......... .......... .......... .......... .......... .......... .......... ..........
Mitrastema         .......... .......... .......... ....CAGTAC TGATTCGTAT GGAATTAGCA CGACCCGGCG ATCAAATTCT TGGTGGAAAT CATCAACTT.
Barringtonia       .......... .......... .......... .......... .......... .......... .......... .......... .......... ......CTT.
Daucus             GGTGCCATTG CTGGAGTGAT GGGCACATGC TTCTCAGTAC TGATTCGTAT GGAATTAGCA CGACCCGGCG ATCAAATTCT TGGCGGGAAT CATCAACTT.
Hydrocotyle        .......... .......... .......... .......... .......... .......... .......... .......... .......... ..........
Helianthus         .......... .......... .......... ....CAGTAC TGATTCGTAT GGAATTAGCA CGACCCGGCG ATCAAATTCT TGGTGGGAAT CATCAACTT.
Alstonia           GGTGCCATTG CTGGAGTGAT GGGCACATGC TTCTCAGTAC TGATTCGTAT GGAATTAGCG CGACCCGGCG ATCAAATTCT TGGTGGGAAT CATCAACTT.
Vinca              .......... .......... .......... .......... .......... .......... .......... .......... .......... ..........
Nerium             .......... .......... .......... .......... .......... .......... .......... .......... .......... ..........
Alyxia             GGTGCCATTG CTGGAGTGAT GGGCACATGC TTCTCAGTAC TGATTCGTAT GGAATTAGCA CGACCCGGCG ATCAAATTCT TGGTGGGAAT CATCAACTT.
Ochrosia           GGTGCCATTG CTGGAGTGAT GGGCACATGC TTCTCAGTAC TGATTCGTAT GGAATTAGCA CGACCCGGCG ATCAAATTCT TGGTGGGAAT CATCAACTT.
Carissa            GGTGCCATTG CTGGAGTGAT GGGCACATGC TTCTCAGTAC TGATTCGTAT GGAATTAGCA CGACCCGGCG ATCAAATTCT TGGTGGGAAT CATCAACTT.
Frasera            .......... .......... .......... ....CAGTAC TGATTCGTAT GGAATTAGCA CGACCCGGCG ATCAAATTCT TGGTGGGAAT CATCAACTT.
Coffea             .......... .......... .......... .......... .......... .......... .......... .......... .......... ......CTT.
Ixora              .......... .......... .......... .......... .......... .......... .......... .......... .......... ......CTT.
Heliotropium       .......... .......... .......... .......... .......... .......... .......... .......... .......... ..........
Ehretia            .......... .......... .......... .......... .......... .......... .......... .......... .......... ......CTT.
Borago             .......... .......... .......... ....CAGTAC TGATTCGTAT GGAATTAGCA CGACCCGGCG ATCAAATTCT TGGTGGGAAT CATCAACTT.
Ipomoea            .......... .......... .......... .......... .......... .......GCA CGACCCGGCG ATCAAATTCT TGGTGGGAAT CATCAACTT.
Schizanthus        .......... .......... ...CACATGC TTCTCAGTAC TGATTCGTAT GGAATTAGCA CGACCCGGCG ATCAAATTCT TGGTGGGAAT CATCAACTT.
Goetzea            .......TTG CTGGAGTGAT GGGCACATGC TTCTCAGTAC TGATTCGTAT GGAATTAGCA CGACCCGGCG ATCAAATTCT TGGTGGGAAT CATCAACTT.
Protoschwenckia    .......TTG CTGGAGTGAT GGGCACATGC TTCTCAGTAC TGATTCGTAT GGAATTAGCA CGACCCGGCG ATCAAATTCT TGGTGGGAAT CATCAACTT.
Bru.densiflora     ..TGCCATTG CTGGAGTGAT GGGCACATGC TTCTCAGTAC TGATTCGTAT GGAATTAGCA CGACCCGGCG ATCAAATTCT TGGTGGGAAT CATCAACTT.
Bru.jamaicensis    GGTGCCATTG CTGGAGTGAT GGGCACATGC TTCTCAGTAC TGATTCGTAT GGAATTAGCA CGACCCGGCG ATCAAATTCT TGGTGGGAAT CATCAACTT.
Bru.grandiflora    GGTGCCATTG CTGGAGTGAT GGGCACATGC TTCTCAGTAC TGATTCGTAT GGAATTAGCA CGACCCGGCG ATCAAATTCT TGGTGGGAAT CATCAACTT.
Nicotiana          GGTGCCATTG CTGGAGTGAT GGGCACATGC TTCTCAGTAC TGATTCGTAT GGAATTAGCA CGACCCGGCG ATCAAATTCT TGGTGGGAAT CATCAACTT.
Atropa             GGTGCCATTG CTGGAGTGAT GGGCACATGC TTCTCAGTAC TGATTCGTAT GGAATTAGCA CGACCCGGCG ATCAAATTCT TGGTGGGAAT CATCAACTT.
Anisodus           .......... .......... .......... .......... .......... .......... .......... .......... .......... .........-
Atropanthe         .......... .......... .......... .......... .......... .......... .......... .......... .......... .........-
Phys.orientalis    .......... ........AT GGGCACATGC TTCTCAGTAC TGATTCGTAT GGAATTAGCA CGACCCGGCG ATCAAATTCT TGGTGGGAAT CATCAACTT.
Phys.infundib.     .......... .......... .......... .......... .......... .......... .......... .......... .......... .........-
Przewalskia        .......... .......... .......... .......... .......... .......... .......... .......... .......... .........-
Hyos.pusillus      .......... .......... .......... .......... .......... .......... .......... .......... .......... .........-
Hyos.aureus        .......... ........AT GGGCACATGC TTCTCAGTAC TGATTCGTAT GGAATTAGCA CGACCCGGCG ATCAAATTCT TGGTGGGAAT CATCAACTT.
Lycium             .....CATTG CTGGAGTGAT GGGCACATGC TTCTCAGTAC TGATTCGTAT GGAATTAGCA CGACCCGGCG ATCAAATTCT TGGTGGGAAT CATCAACTT.
Jaborosa           .........G CTGGAGTGAT GGGCACATGC TTCTCAGTAC TGATTCGTAT GGAATTAGCA CGACCCGGCG ATCAAATTCT TGGTGGGAAT CATCAACTT.
Exodeconus         ........TG CTGGAGTGAT GGGCACATGC TTCTCAGTAC TGATTCGTAT GGAATTAGCA CGACCCGGCG ATCAAATTCT TGGTGGGAAT CATCAACTT.
Juanulloa          .......TTG CTGGAGTGAT GGGCACATGC TTCTCAGTAC TGATTCGTAT GGAATTAGCA CGACCCGGCG ATCAAATTCT TGGTGGGAAT CATCAACTT.
Man.officinarum    .......... .......... .......... .......... .......... .......... .......... .......... .......... .........-
Man.autumnalis     .......... .......... .......... .......... .......... .......... .......... .......... .......... .........-
Man.caulescens     .......... .......... .......... .......... .......... .......... .......... .......... .......... .........-
Solanum            GGTGCCATTG CTGGAGTGAT GGGCACATGC TTCTCAGTAC TGATTCGTAT GGAATTAGCA CGACCCGGCG ATCAAATTCT TGGTGGGAAT CATCAACTT.
Calceolaria        .......... .......... .......... .......... .......... .......... .......... .......... .......... ......CTT.
Drymonia           .......... .......... .......... .......... .......... .......... .......... .......... .......... ......CTT.
Nematanthus        .......... .......... .......... .......... .......... .......... .......... .......... .......... ......CTT.
Veronica           .......... .......... .......... .......... .......... .......... .......... .......... .......... ..........
Digitalis          .......... .......... .......... .......... .......... .......... .......... .......... .......... ..........
Celsia             .......... .......... .......... .......... .......... .......... .......... .......... .......... ......CTT.
Verbascum          .......... .......... .......... .......... .......... .......... .......... .......... .......... .........-
Justicia           .......... .......... .......... .......... .......... .......... .......... .......... .......... ......CTT.
Barleria           .......... .......... .......... .......... .......... .......... .......... .......... .......... ......CTT.
Thunbergia         .......... .......... .......... .......... .......... .......... .......... .......... .......... ......CTT.
Sanchezia          .......... .......... .......... .......... .......... .......... .......... .......... .......... ..........
Catalpa            .......... .......... .......... .......... .......... .......... .......... .......... .......... ..........
Campsis            GGTGCCATTG CTGGAGTGAT GGGCACATGC TTTTCAGTAC TGATTCGTAT GGAATTAGCA CGACCCGGCG ATCAAATTCT TGGTGGGAAT CATCAACTT.
Ajuga              .......... .......... .......... .......... .......... .......... .......... .......... .......... ......CTT.
Callicarpa         .......... .......... .......... .......... .......... .......... .......... .......... .......... .........-
Sesamum            .......... .......... .......... .......... .......... .......... .......... .......... .......... ......CTT.
```

```
                  101        111        121        131        141        151        161        171        181        191
                  |          |          |          |          |          |          |          |          |          |
Rhamnus           ATAATGTTTT AATAACGGCT CACGCTTTTT TAATGATATT TTTTATGGTT ATGCCGGCGA TGATAGGTGG ATCTGGTAAT TGGTCTGTTC CGATTCTGAT
Asimina           .......... .......... ......CC.. .......C.. .......... .......... .......... .......... .......... .......T..
Magnolia          .......... .......... ......CC.. .......C.. .......... .......... .......... .......... .......... ..........
Knema             ..T....... .......... ......CC.. .......C.. .......... .......... .......... .......... .......... ..........
Myristica         .......... .......... ......CC.. .......C.. .......... .......... .......... .......... .......... ..........
Peperomia         ---------- ---------- ---------- ---------- ---------- ---------- ---------- ---------- ---------- ----------
Piper             .......... .......... .......... .......C.. .......... .......... .......... .......... .......... ..........
Arisaema          .......... .......... .......... .......C.. .......... .......... .......... .......... .......... ..........
Zamioculcas       .......... .......... .......... .......C.. .......... .......... .......... .......... .......... ..........
Xanthosoma        .......... .......... .......... .......C.. .......... .......... .......... .......... .......... ..........
Philodendron      .......... .......... .......... .......C.. .......... .......... .......... .......... .......... ..........
Peltandra         .......... .......... .......... .......C.. .......... .......... .......... .......... .......... ..........
Strelitzia        .......A.. .......... .......C.. .......C.. .......... .......... .......... .......... .......... ..........
Musa              .......G.. .......... .......C.. .......C.. .......... .......... .......... ......A... ....T...A. .......T..
Musella           ---------- ---------- ---------- ---------- ---------- ---------- ---------- ---------- ---------- ----------
Maranta           .......A.. ...T...... .......... .......C.. .......... .......... .......... .......... .......... ..........
Monotagma         ---------- ---------- ---------- ---------- ---------- ---------- ---------- ---------- ---------- ----------
Haumania          ---------- ---------- ---------- ---------- ---------- ---------- ---------- ---------- ---------- ----------
Globba            ---------- ---------- ---------- ---------- ---------- ---------- ---------- ---------- ---------- ----------
Costus            .......C.. .......... .......C.. .......C.. .......... .......... .......... .......... .......... ..........
Triticum          .......... .......... ..T....... .......C.. .......... .......... .......... ..T....... ....T..... ..........
Grevillea         .......... .......... .......C.. .......C.. .......... .......... .......... .......... .......... ..........
Melia             .......... .......... .......... .......C.. .......... .......... .......... .......... .......... ..........
Dysoxylum         .......... .......... .......... .......C.. .......... .......... .......... .......... .......... ..........
Ailanthus         .......... .......... .......... .......C.. .......... .......... .......... .......... .......... ..........
Toxicodendron       ---------- ---------- ---------- ---------- ---------- ---------- ---------- ---------- ---------- ----------
Rhus              .......... .......... .......... .......C.. .......... .......... .......... .......... .......... ..........
Bur.sp            .......... .......... .......... .......C.. .......... .......... .......... .......... .......... ..........
Bur.simarouba     .......... .......... ..T....... .......C.. .......... .......... .......... .......... .......... ..........
Breynia           .......... ......A... .......... .......C.. .......... .......... .......... .......... .......... ..........
Phyllanthus       .......... ......A... .......... .......C.. .......... .......... .......... .......... .......... ..........
Hevea             .......... .......... .......... .......C.. .......... .......... .......... .......... .......... ..........
Acalypha          .......... .......... .......... .......C.. .......... .......... .......... .......... .......... ..........
Croton            .......... .......... .......... .......C.. .......... .......... .......... .......... .......... ..........
Euphorbia         .......... .......... .......... .......C.. .......... .......... .......... .......... .......... ..........
Hura              .......... .......... .......... .......C.. .......... .......... .......... .......... .......... ..........
Malpighia         .......... .......... .......... .......C.. .......... .......... .......... ..T....... ....T..... .A........
Polygala          .......... .......... .......... .......C.. .......... .......... .......... .......... .......... ..........
Humulus           .......... .......... .......... .......C.. .......... .......... .......... ..T....... ....T..... ..........
Pilea             .......... ......A... .......... .......C.. .......... .......... .......... ..T....... ....T..... ..........
Hovenia           .......... .......... .......... .......T.. .......... .......... .......... .......... .......... .......T..
Cyn.songaricum    .......... .......... .......... .......C.. .......... .......... .......... ..T....... ....T..... ..........
Cyn.coccineum     .......... .......... .......... .......C.. .......... .......... .......... ..T....... ....T..... ..........
Citrullus         .......... .......... .......... .......C.. .......... .......... .......... .......... .......... ..........
Melothria         .......... .......... .......... .......C.. .......... .......... .......... .......... .......... ..........
Cucurbita         .......... .......... .......... .......C.. .......... .......... .......... .......... .......... ..........
Lepionurus        .......... .......... .......... .......C.. .......... .......... .......... .......... .......... ..........
Andromeda         ---------- ---------- ---------- ---------- ---------- ---------- ---------- ---------- ---------- ----------
Pyrola            .......... .......... .......... .......C.. .......... .......... .......... .......... .......... ..........
Symplocus         .......... .......... .......... .......C.. .......... .......... .......... .......... .......... ..........
Diospyros         .......... .......... .......... .......C.. .......... .......... .......... .......... .......... ..........
Mitrastema        .......... .......... .......... .......C.. .......... .......... .......... .......... .......... ..........
Barringtonia      .......... .......... .......... .......C.. .......... .......... .......... .......... .......... ..........
Daucus            .......... .......... .....C.... .......C.. .......... .......... .......... .......... .......... ..........
Hydrocotyle       .......... .......... .......... .......C.. .......... .......... .......... .......... .......... ..........
Helianthus        .......... .......... .......... .......C.. .......... .......... .......... .......... .......... ..........
Alstonia          .......... .......... .......... .......C.. .......... .......... .......... .......... .......... ..........
Vinca             .......... .......... .......... .......C.. .......... .......... .......... .......... .......... ..........
Nerium            .......... .......... .......... .......C.. .......... .......... .......... .......... .......... ..........
Alyxia            .......... .......... .......... .......C.. .......... .......... .......... .......... .......... ..........
Ochrosia          .......... .......... .....C.... .......C.. .......... .......... .......... .......... .......... ..........
Carissa           .......... .......... .......... .......C.. .......... .......... .......... .......... .......... ..........
Frasera           .......... .......... .......... .......C.. .......... .......... .......... .......... .......... ..........
Coffea            .......... .......... .......... .......C.. .......... .......... .......... .......... ....T..... ..........
Ixora             .......... .......... .......... .......C.. .......... .......... .......... .......... ....T..... ..........
Heliotropium      .......... .......... .......... .......C.. .......... .......... ..G....... ..T....... .......... T.........
Ehretia           .......... .......... .......... .......C.. .......... .......... .......... .......... .......... ..........
Borago            .......... .......... .......... .......C.. .......... .......... .......... .......... .......... ..........
Ipomoea           .......... .......... .......... .......... .......... .......... .......... .......... .......... ..........
Schizanthus       .......G.. .......... .......... .......C.. .......... .......... .......... .......... .......... ..........
Goetzea           .......... .......... .......... .......C.. .......... .......... .......... .......... .......... ..........
Protoschwenckia   .......... .......... .......... .......C.. .......... .......... .......... .......... .......... ..........
Bru.densiflora    .......... .......... .......... .......C.. .......... .......... .......... .......... .......... ..........
Bru.jamaicensis   .......... .......... .......... .......C.. .......... .......... .......... .......... .......... ..........
Bru.grandiflora   .......... .......... .......... .......C.. .......... .......... .......... .......... .......... ..........
Nicotiana         .......... .......... .......... .......C.. .......... .......... .......... .......... .......... ..........
Atropa            .......... .......... .......... .......C.. .......... .......... .......... .......... .......... ..........
Anisodus          ---------- ---------- ---------- ---------- ---------- ---------- ---------- ---------- ---------- ----------
Atropanthe        ---------- ---------- ---------- ---------- ---------- ---------- ---------- ---------- ---------- ----------
Phys.orientalis   .......... .......... .......... .......C.. .......... .......... .......... .......... .......... ..........
Phys.infundib.    ---------- ---------- ---------- ---------- ---------- ---------- ---------- ---------- ---------- ----------
Przewalskia       ---------- ---------- ---------- ---------- ---------- ---------- ---------- ---------- ---------- ----------
Hyos.pusillus     ---------- ---------- ---------- ---------- ---------- ---------- ---------- ---------- ---------- ----------
Hyos.aureus       .......... .......... .......... .......C.. .......... .......... .......... .......... .......... ..........
Lycium            .......... .......... .......... .......C.. .......... .......... .......... .......... .......... ..........
Jaborosa          .......... .......... .......... .......C.. .......... .......... .......... .......... .......... ..........
Exodeconus        .......... .......... .......... .......C.. .......... .......... .......... .......... .......... ..........
Juanulloa         .......... .......... .......... .......C.. .......... .......... .......... .......... .......... ..........
Man.officinarum   ---------- ---------- ---------- ---------- ---------- ---------- ---------- ---------- ---------- ----------
Man.autumnalis    ---------- ---------- ---------- ---------- ---------- ---------- ---------- ---------- ---------- ----------
Man.caulescens    ---------- ---------- ---------- ---------- ---------- ---------- ---------- ---------- ---------- ----------
Solanum           .......... .......... .......... .......C.. .......... .......... .......... ..T....... .......... ..........
Calceolaria       .......... .......... .....C.... .......C.. .......... .......... .......... .......... .......... ..........
Drymonia          .......... .......... .....C.... .......C.. .......... .......... .......... .......... .......... ..........
Nematanthus       .......... .......... .....C.... .......C.. .......... .......... .......... .......... .......... ..........
Veronica          .......... .......... .....C.... .......C.. .......... .......... .......... .......... .......... ..........
Digitalis         .......... .......... .....C.... .......C.. .......... .......... .......... .......... .......... ..........
Celsia            .......... .......... .....C.... .......C.. .......... .......... .......... .......... .......... ..........
Verbascum         ---------- ---------- ---------- ---------- ---------- ---------- ---------- ---------- ---------- ----------
Justicia          .......... .......... .......... .......C.. .......... ........A. .......... .......... ......A... .C.....C..
Barleria          .......... .......... .....C.... .......C.. .......... .......... .......... .......... .......... ..........
Thunbergia        .......... .......... .....C.... .......C.. .......... .......... .......... .......... .......... ..........
Sanchezia         .......... .......... .....C.... .......C.. .......... ........A. .......... .......... .......... ..........
Catalpa           .......... .......... .....C.... .......C.. .......... .......... .......... .......... .......... ..........
Campsis           .......... .......... .....C.... .......C.. .......... .......... .......... .......... .......... ..........
Ajuga             .......... .......... .....C.... .......C.. .......... .....A.... .......... .......... .......... ..........
Callicarpa        ---------- ---------- ---------- ---------- ---------- ---------- ---------- ---------- ---------- ----------
Sesamum           .......... .......... .....C.... .......C.. .......... .......... .......... .......... .......... ..........
```

```
                 201        211        221        231        241        251        261        271        281        291
                 |          |          |          |          |          |          |          |          |          |
Rhamnus          AGGTGCACCT GACATGGCAT TTCCACGATT AAATAATATT TCATTCTGGT TGTTGCCACC AAGTCTCTTG CTCCTATTAA GCTCAGCCTT AGTAGAAGTG
Asimina          .......... .......... .........C .......... .......... .......... .......... .......... ..C....... ..........
Magnolia         .......... .......... .........C .......... .......... C......... .......... .......... ..C....... ..........
Knema            .......... .......... .........C .......... .......... .......... .......... .......... ..C....... ..........
Myristica        .......... .......... .........C .......... .......... .......... .......... .......... ..C....... ..........
Peperomia        ---------- ---------- ---------- ---------- ---------- ---------- ---------- ---------- ---------- ----------
Piper            .......... .......... .........C .......... .......... .......... ....A..... .......... ..C....... .........A
Arisaema         .......... .......... .......... .........A .......... .......... .......... .......... ..C....... ..........
Zamioculcas      .......... .......... .......... .........A .......... .......... .......... .......... ..C....... ..........
Xanthosoma       .......... .......... .......... .........A .......... .......... .......... .......... ..C....... ..........
Philodendron     .......... .......... .......... .........A .......... .......... .......... .......... ..C....... ..........
Peltandra        .......... .......... .......... .........A .......... .......... .......... .......... ..C....... ..........
Strelitzia       .......... .......... .......... .........C .......... .......... .......... .......... ..C.....C. ..........
Musa             .......... .......... .......... .........A .......... .......... .......... .......... ........C. ..........
Musella          ---------- ---------- ---------- ---------- ---------- ---------- ---------- ---------- ---------- ----------
Maranta          .......... .......... .......... .........C .......... .......... .......... .......... ..C.....C. ..........
Monotagma        ---------- ---------- ---------- ---------- ---------- ---------- ---------- ---------- ---------- ----------
Haumania         ---------- ---------- ---------- ---------- ---------- ---------- ---------- ---------- ---------- ----------
Globba           ---------- ---------- ---------- ---------- ---------- ---------- ---------- ---------- ---------- ----------
Costus           .......... .......... .......... .........C .......... .......... .......... .......... ..C.....C. ..........
Triticum         .......... .......... .......... .........A .......... .......... .......... .......... .......... ..........
Grevillea        .......... .......... .........C .......... .......... .......... .......... .......... ..C....... ..........
Melia            .......... .......... .......... .......... .......... .......... .......... .......... .......... ..........
Dysoxylum        .......... .......... .......... .......... .......... .......... .......... .......... .......... ..........
Ailanthus        .......... .......... .......... .......... .......... .......... .......... .......... .......... ..........
Toxicodendron      ---------- ---------- ---------- ---------- ---------- ---------- ---------- ---------- ---------- ----------
Rhus             .......... .......... .......... .......... .......... .......... .......... .......... .......... ..........
Bur.sp           .......... .......... .......... .......... .......... .......... .......... .......... .......... ..........
Bur.simarouba    .......... .......... .......... .......... .......... .......... .......... .......... .......... ..........
Breynia          .........C .......... .......... .......... .......... .......... .........A .......... .......... ..........
Phyllanthus      .........C .......... .......... .......... .......... .......... .........A .......... .......... ..........
Hevea            .......... .......... .......... .......... .......... .......... .........A .......... .......... ...G......
Acalypha         .......... .......... .......... .......... .......... .......... .........A .......... .......... ..........
Croton           .......... .......... .......... .......... .......... .......... ....A....A .......... .......... ...G......
Euphorbia        .......... .......... .......... .......... .......... .......... .........A .......... .......... ...G......
Hura             .......... .......... .......... .......... .......... .......... .........A .......... .......... ...G......
Malpighia        .......... .......... .......... .......... .....T.... .......... .........A .......... .......... .........T
Polygala         ......G... .......... .......... .......... .......... .......... .......... .......... .......... ..........
Humulus          .......... .......... .......... .......... .......... .......... .......... .......... .......... ..........
Pilea            .......... .......... .......... .......... .......... .......... .......... .......... .......... ..........
Hovenia          .......... .......... .......... .......... .......... .......... .......... .......... .......... ..........
Cyn.songaricum   .......... .......... .......... .......... .......... .......... .......... .......... .......... .........A
Cyn.coccineum    .......... .......... .......... .......... .......... .......... .......... .......... ....C..... .........A
Citrullus        .......... .......... .......... .......... .......... .......... .......... .......... .......... ..........
Melothria        .......... .......... .......... .......... .......... .......... .......... .......... .......... ..........
Cucurbita        .......... .......... ....C..... .......... .......... .......... .......... .......... .......... ..........
Lepionurus       .......... .......... ....G..... .......... .......... .......... .......... .......... ..C....... ..........
Andromeda        ---------- ---------- ---------- ---------- ---------- ---------- ---------- ---------- ---------- ----------
Pyrola           ......G... .......... .......... .......... .......... .......... .......... .......... .......... ..........
Symplocus        ......G... .......... .......... .......... .......... .......... .......... .......... .......... ..........
Diospyros        ......G... .......... .......... .......... .......... .......... ...C...... .......... .......... ..........
Mitrastema       .......... .......... .......... .......C.G .......... .......G.. ......T... .......... .......... ..........
Barringtonia     ......G... .......... .......... .......... .......... .......... .......... .......... ..C....... ..........
Daucus           ......G... .......... .......... .......... .......... .......... .......... .......... ..C....... ..........
Hydrocotyle      ......G... .......... .......... .......... .......... .......... .......... .......... ..C....... ..........
Helianthus       ......G... .......... .......... .......... .......... .......... .......... .......... ..C....... ..........
Alstonia         ...G..G... .......... .......... .......... .......... .......... .......... .......... .......... ..........
Vinca            ...G..G... .......... .......... .......... .......... .......... .......... .......... .......... ..........
Nerium           ...G..G... .......... .......... .......... .......... .......... .......... .......... .......... ..........
Alyxia           ...G..G... .......... .......... .......... .......... .......... .......... .......... .......... ..........
Ochrosia         ...G..G... .......... .......... .......... .......... .......... .......... .......... .......... ..........
Carissa          ...G..G... .......... .......... .......... .......... .......... .......... .......... .......... ..........
Frasera          ......G... .......... .......C.. .......... .......... .......... .......... .......... ..C....... ..........
Coffea           ......G... .......... .......... .......... .......... .......... .......... ...T...... .......... ..........
Ixora            ......G... .......... .......... .......... .......... .......... .......... ...T...... .......... ..........
Heliotropium     ......G... .......... .......... .......... .......... ...G...... .......... .......... ..C....... ....A.....
Ehretia          ......G... .......... .......... .......... .......... .......... .......... .......... ..C....... ..........
Borago           ...A..G... .......... .......... .......... .......... .......... .......... .......... ..C....... ..........
Ipomoea          ......G... .......... .......... .......... .......... .......T.. .......... .......... ..C....... ..........
Schizanthus      ......G... .......... .......... .......... .......... .......T.. .......... .......... ..C....... ..........
Goetzea          ......G... .......... .C........ .......... .......... .......T.. .......... .......... ..C....... ..........
Protoschwenckia  ......G... .......... .......... .......... .......... .......T.. .......... .......... ..C....... ..........
Bru.densiflora   ......G... .......... .......... .......... .......... .......T.. .......... .......... ..C....... ..........
Bru.jamaicensis  ......G... .......... .......... .......... .......... .......T.. .......... .......... ..C....... ..........
Bru.grandiflora  ......G... .......... .......... .......... .......... .......T.. .......... .......... ..C....... ..........
Nicotiana        ......G... .......... .......... .......... .......... .......T.. .......... .......... .......... ..........
Atropa           ......G... .......... .......... .......... .......... .......T.. .......... .......... .......... ..........
Anisodus         ---------- ---------- ---------- ---------- ---------- ---------- ---------- ---------- ---------- ----------
Atropanthe       ---------- ---------- ---------- ---------- ---------- ---------- ---------- ---------- ---------- ----------
Phys.orientalis  ......G... .......... .......... .......... .......... .......T.. .......... .......... .......... ..........
Phys.infundib.   ---------- ---------- ---------- ---------- ---------- ---------- ---------- ---------- ---------- ----------
Przewalskia      ---------- ---------- ---------- ---------- ---------- ---------- ---------- ---------- ---------- ----------
Hyos.pusillus    ---------- ---------- ---------- ---------- ---------- ---------- ---------- ---------- ---------- ----------
Hyos.aureus      ......G... .......... .......... .......... .......... .......T.. .......... .......... .......... ..........
Lycium           ......G... .......... .......... .......... .......... .......T.. .......... .......... .......... ..........
Jaborosa         ......G... .......... ....G..... .......... .......... .......T.. .......... .......... .......... ..........
Exodeconus       ......G... .......... .......... .......... .......... .......T.. .......... .......... .......... ..........
Juanulloa        ......G... .......... .......... .......... .......... .......T.. .......... .......... ..C....... ..........
Man.officinarum  ---------- ---------- ---------- ---------- ---------- ---------- ---------- ---------- ---------- ----------
Man.autumnalis   ---------- ---------- ---------- ---------- ---------- ---------- ---------- ---------- ---------- ----------
Man.caulescens   ---------- ---------- ---------- ---------- ---------- ---------- ---------- ---------- ---------- ----------
Solanum          ......G... .......... .......... .......... .......... .......T.. .......... .......... .......... ..........
Calceolaria      ......G... .......... .......... .......... .......... .......... .......... .......... .......... ..........
Drymonia         ......G... .......... .......... .......... .......... .......... .......... .......... .......... ..........
Nematanthus      ......G... .......... .......... .......... .......... .......... .......... .......... .......... ..........
Veronica         ......G... .......... .......... .......... .......... .......... .......... .......... .......... ..........
Digitalis        ......G... .......... .......... .......... .......... .......... .......... .......... .......... ..........
Celsia           ......G... .......... .......... .......... .......... .......... .......... .......... .......... ..........
Verbascum        ---------- ---------- ---------- ---------- ---------- ---------- ---------- ---------- ---------- ----------
Justicia         ......G... .......... .......... .......... .......... .C........ .......... .......... .......... ..........
Barleria         ......G... .......... .......... .......... .......... .......... .......... .......... .......... ..........
Thunbergia       ......G... .......... .......... .......... .......... .......... .......... .......... .......... ..........
Sanchezia        ......G... .......... .......... .......... .......... .......... .......... .......... .......... ..........
Catalpa          ......G... .......... .......... .......... .......... .......... .......... .......... .......... ..........
Campsis          ......G... .......... .......... .......... .......... .......... .......... .......... .......... ..........
Ajuga            ......G... .......... .......... .......... .......... .......... G......... .......... .......... ..........
Callicarpa       ---------- ---------- ---------- ---------- ---------- ---------- ---------- ---------- ---------- ----------
Sesamum          ......G... .......... .......... .......... .......... .......... .......... .......... .......... ..........
```

```
                   301        311        321        331        341        351        361        371        381        391
                   |          |          |          |          |          |          |          |          |          |
Rhamnus            GGTAGCGGCA CTGGGTGGAC GGTCTATCCG CCCTTAAGTG GTATTACCAG CCATTCTGGA GGAGCAGTTG ATTTAGCAAT TTCTAGTCTT CATCTATCTG
Asimina            .......... .......... .......... .......... .......... .......... .......... ...C...... ........C. ........A.
Magnolia           .......... .......... .......... .......... .......... .......... .......... ...C...... ........C. ........A.
Knema              .......... .......... .......... .......... .......... .......... .......... ...C...... .......... ..........
Myristica          .......... .......... .......... .......... .......... .......... .......... ...C...... .......... ..........
Peperomia          ---------- ---------- ---------- ---------- ---------- ---------- ---------- ---------- ---------- ----------
Piper              .......... .......... .......... .......... .......... .......... .......... ...C...... ........C. ........A.
Arisaema           .......... .......... .......... .......... .......... .......... .......... ...C...... .......... ........A.
Zamioculcas        .......... .......... .......... .......... .......... .......... .......... ...C...... .......... ........A.
Xanthosoma         .......... .......... .......... .......... .......... .......... .......... ...C...... .......... ........A.
Philodendron       ........T. .......... .......... .......... .......... .......... .......... ...C...... .......... ........A.
Peltandra          .......... .......... .......... .......... .......... .......... .......... ...C...... .......... ........A.
Strelitzia         ....C..... .......... .......... .......... .......... .......... .......... ...C...... ........C. ........A.
Musa               ....C..... .......... .......... .......... .......... .......... .......... ...C...... .......... .....C..A.
Musella            ---------- ---------- ---------- ---------- ---------- ---------- ---------- --.C...... .......... .....C..A.
Maranta            ....C..... .......... .......... .......... .......... .......... .......... ...C...... ........C. ........A.
Monotagma          ---------- ---------- ---------- ---------- ---------- ---------- ---------- --.C...... ........C. ........A.
Haumania           ---------- ---------- ---------- ---------- ---------- ---------- ---------- --.C...... ........C. ........A.
Globba             ---------- ---------- ---------- ---------- ---------- ---------- ---------- ---------- ---------- ----------
Costus             ....C..... .......... .......... .......... .......... .......... .......... ...C...... ........C. ........A.
Triticum           ..C....... .......... A......... .......... .......... .......... .......... .......... ..T....... ........A.
Grevillea          .......... .......... .......... .......... .......... .......... .......... ...C...... ........C. ..........
Melia              .......... .......... .......... .......... .......... .......... .......... ...C...... .......... ..........
Dysoxylum          .......... .......... .......... ...AAC.... .......... .......... .......... ...C...... .......... ..........
Ailanthus          .......... .......... .......... .......... .......... .......... .......... ...C...... .......... ..........
Toxicodendron        ---------- ---------- ---------- ---------- ---------- ---------- ---------- ---------- ---------- ----------
Rhus               .......... .......... .......... .......... .......... .......... .......... ...C...... .......... ..........
Bur.sp             .......... .......... .......... .......... .......... .......... .......... ...C...... .......... ..........
Bur.simarouba      .......... .......... .......... .......... .......... .......... .......... ...C...... .......... ..........
Breynia            .......... .......... .......... .......... .......... ...C...... .....T.... .......... .......... ..........
Phyllanthus        .......... .......... .......... .......... .......... ...C...... .....T.... .......... .......... ..........
Hevea              .......... .......... .......... .......... .......... .......... .......... ...C...... .......... ..........
Acalypha           .......... .......... .......... .......... .......... .......... .......... ...C...... .......... ..........
Croton             .......... .......... .......... .......... .......... .......... .......... ...C...... .......... ..........
Euphorbia          .......... .......... .........C .......... .......... .......... .......... ...C...... .......... ..........
Hura               .......... .......... .......... .......... .......... .......... .......... ...C...... .......... ..........
Malpighia          .......... .......... .......... .......... .......... .......... .......... .......... ..T....... ..........
Polygala           .......... .......... .......... .......... .......... .......... .......... .......... .......... ..........
Humulus            .......... .......... .......... .......... .......... .......... .......... .......... ..T....... ..........
Pilea              .......... .......... .......... .......... .......... .......... .......... .......... ..T....... ..........
Hovenia            .......... .......... .......... .......... .......... .......... .....T.... .......... .......... ..........
Cyn.songaricum     .......... .......... .......... ..T....... .......... .......... .......... .......T.. ..T....... ..........
Cyn.coccineum      .......... .......... .......... ..T....... .......... .......... .......... .......C.. ..T....... ..........
Citrullus          .......... .......... .......... .......... .......... .......... .......... ...C...... .......... ..........
Melothria          .......... .......... .......... .......... .......... .......... .......... ...C...... .......... ..........
Cucurbita          .......... .......... .......... .......... .......... .......... .......... ...C...... .......... ..........
Lepionurus         .......... .......... .......... .......... .......... .......... .......... ...C...... .......... ..........
Andromeda          ---------- ---------- ---------- ---------- ---------- ---------- ---------- ---------- ..T....... ----------
Pyrola             .......... .......... .......... .......... .......... .......... .......... ....T..... ..T....... ..........
Symplocus          .......... .......... .......... .......... .......... .......... .......... .......... .......... ..........
Diospyros          .......... .......... .......... .......... .......... .......... .......... .......... ..T....... ........C.
Mitrastema         .......... .......... .......... ..T....... .......... .......... .......... ...C...... C......... ..........
Barringtonia       .......... .......... .......... .......... .......... .......... .......... ...C...... .......... ..........
Daucus             .......... .......... .......... .......... .......... .......... .......... ...C...... ........C. ........C.
Hydrocotyle        .......... .......... .......... .......... .......... .......... .......... ...C...... ........C. ..........
Helianthus         .....T.... .......... .......... .......... .......... .......... .......... ...C...... ........C. ..........
Alstonia           ........A. .......... .......... .......... .......... .......... .......... ...C...... .......... ..........
Vinca              ........A. .......... .......... .......... .......... .......... .......... ...C...... .......... ..........
Nerium             ........A. .......... ......C... .......... .......... .......... .......... ...C...... .......... ..........
Alyxia             ........A. .C........ .......... .......... .......... .......... .......... ...C...... .......... ..........
Ochrosia           ........A. .......... .......... .......... .......... .......... .......... ...C...... .......... ..........
Carissa            ........A. .......... .......... .......... .......... .......... .......... ...C...... .......... ..........
Frasera            ........A. .......... .......... .......... .......... .......... .......... .......... .......... ..........
Coffea             ........A. .......... .......... .......... .......... .......... .......... ...C...... .......... ..........
Ixora              ........A. .......... .......... .......... .......... .......... .......... ...C...... .......... ..........
Heliotropium       .....A..A. .......... .......... .......... ....W..... .......... .......... ...C...... .......... ..........
Ehretia            ........A. .......... .......... .......... .......... .......... .......... ...C...... .......... ..........
Borago             ........A. .......... .......... .......... .......... .......... .......... ...C...... .......... ..........
Ipomoea            ........A. .......... .......... .......... .......... .........T .......... ...C...... .......... ..........
Schizanthus        .......... .......... .......... .......... .......... .......... .......... .......... .......... ..........
Goetzea            .......... .......... .......... .......... .......... .......... .......... .......... .......... ..........
Protoschwenckia    .......... .......... .......... .......... .......... .......... .......... .......... .......... ..........
Bru.densiflora     .......... .......... .......... .......... .......... .......... .......... .......... .......... ..........
Bru.jamaicensis    .......... .......... .......... .......... .......... .......... .......... .......... .......... ..........
Bru.grandiflora    ........T. .......... .......... .......... .......... .......... .......... .......... .......... ..........
Nicotiana          .......... .......... .......... .......... .......... .......... .......... .......... .......... ..........
Atropa             .......... .......... .......... .......... .......... .......... .......... .......... .......... ..........
Anisodus           ---------- ---------- ---------- ---------- ---------- ---------- ---------- ---------- ---------- ----------
Atropanthe         ---------- ---------- ---------- ---------- ---------- ---------- ---------- ---------- ---------- ----------
Phys.orientalis    ---------- ---------- ---------- ---------- ---------- ---------- ---------- ---------- ---------- ----------
Phys.infundib.     ---------- ---------- ---------- ---------- ---------- ---------- ---------- ---------- ---------- ----------
Przewalskia        ---------- ---------- ---------- ---------- ---------- ---------- ---------- ---------- ---------- ----------
Hyos.pusillus      ---------- ---------- ---------- ---------- ---------- ---------- ---------- ---------- ---------- ----------
Hyos.aureus        .......... .......... .......... .......... .......... .......... .......... .......... .......... ..........
Lycium             .......... .......... .......... .......... .......... .......... .......... .......... .......... ..........
Jaborosa           .......... .......... .......... .......... .......... .......... .......... .......... .......... ..........
Exodeconus         .......... .......... .......... .......... .......... .......... .......... .......... .......... ..........
Juanulloa          .......... .......... .......... .......... .......... .......... .......... .......... .......... ..........
Man.officinarum    ---------- ---------- ---------- ---------- ---------- ---------- ---------- ---------- ---------- ----------
Man.autumnalis     ---------- ---------- ---------- ---------- ---------- ---------- ---------- ---------- ---------- ----------
Man.caulescens     ---------- ---------- ---------- ---------- ---------- ---------- ---------- ---------- ---------- ----------
Solanum            .......... .......... .......... .......... .......... .......... .......... .......... .......... ..........
Calceolaria        ........A. .......... .......... .......... .......... .......... .......... .......... .......... ..........
Drymonia           ........A. .......... .......... .......... .......... .......... .......... .......... .......... ..........
Nematanthus        ........A. .......... .......... .......... .......... .......... .......... .......... .......... ..........
Veronica           ........A. .......... .......... .......... .......... ......A... .......... .......... .......... ..........
Digitalis          ........A. .......... .......... .......... .......... .......... .......... .......... .......... ..........
Celsia             ........A. .......... .......... .......... .......... .......... .......... .......... .......... ..........
Verbascum          ---------- ---------- ---------- ---------- ---------- ---------- ---------- ---------- ---------- ----------
Justicia           ........A. .......... .......... .......... C......... .......... .......... .......... ..T....... ...T......
Barleria           ........A. .......... .......... .......... .......... .......... .......... .......... .......... ..........
Thunbergia         ........A. .......... .......... .......... .......... .......... .......... .......... .......... ..........
Sanchezia          ........A. .......... .......... .......... .......... .......... .......... .......... .......... ..........
Catalpa            ........A. .......... .......... .......... .......... .......... .......... .......... .......... ..........
Campsis            ........A. .......... .......... .......... .......... .......... .......... .......... .......... ..........
Ajuga              ........A. .......... .......... .......... .......... .......... .......... .......C.. .......... ..........
Callicarpa         ---------- ---------- ---------- ---------- ---------- ---------- ---------- ---------- ------.... ..-.......
Sesamum            ........A. .......... .......... .......... .......... .......... .......... .......... .......... ..........
```

```
                  401        411        421        431        441        451        461        471        481        491
                  |          |          |          |          |          |          |          |          |          |
Rhamnus           GTGTTTCATC CATTTTAGGT TCTATCAATT TTATAACAAC TATCTCCAAC ATGCGTGGAC CTGGAATGAC TATGCATAGA TCACCCCTAT TTGTGTGGTC
Asimina           .......... .......... .......... .......... .......... .......... .......... .......... .T........ ..........
Magnolia          .......... .......... .......... .......... .......... .......... .......... .......... .......... ..........
Knema             .......... .......... .......... .......... .......... .......... .......... .......... .......... ..........
Myristica         .......... .......... .......... .......... .......... .......... .......... .......... .......... ..........
Peperomia         ---------- ---------- ---------- ---------- ---------- ---------- ---------- ---------- ---------- ----------
Piper             .......... T......... .......... .......C.. .......... .......... .......... .......... .......... ..........
Arisaema          .......... A......... .......... .......... .......... .......... .C........ .......... ........T. ..........
Zamioculcas       .......... A......... .......... .......... .......... .......... .C........ .......... ........T. ..........
Xanthosoma        .......... A......... .......... .......... .......... .......... .C........ .......... ........T. ..........
Philodendron      .......... A......... .......... .......... .......... .......... .C........ .......... ........T. ..........
Peltandra         .......... A......... .......... .......... .......... .......... .C........ .......... ........T. ..........
Strelitzia        .......... A......... .......... .......... .......... .......... .......... .......... ........T. ..........
Musa              .......... A........A .......... .......C.. .....TT... .......... .......... .......... ........T. ..........
Musella           .......... A........A .......... .......... .....TT... .......... .......... .......... ........T. ..........
Maranta           .......... .......... .......... .......C.. .......... .......... .......... .......... ........T. ..........
Monotagma         .......... .......... .......... .......C.. .......... .......... .......... .......... ........T. ..........
Haumania          .......... .......... .......... .......C.. .......... .......... .......... .......... ........T. ..........
Globba            ---------- ----...... .......... .A........ .....T.... .......... .......A.. .......... .T.G....T. ..........
Costus            .......... A......... .......... .......... .......... .......... .......T.. .......... ........T. ..........
Triticum          ..A....... A......... .......... .......... .....T.... .......... .......... .......... .T...A..T. ..........
Grevillea         .......... .......... .......... .......... .....T.... .......... .......... .......... .......... ..........
Melia             .......... .......... .......... .......... A......... .......... .......... .......... .........C ..........
Dysoxylum         .......... .......... .......... .......... A......... .......... .......... .......... .......... ..........
Ailanthus         .......... .......... .......... .......... A......... .......... .......... .......... .......... ..........
Toxicodendron       -----..... .......... .......... .......... .......... .......... .......... .......... .....T.... ..........
Rhus              .......... .......... .......... .......... .......... .......... .......... .......... .....T.... ..........
Bur.sp            .......... .......... .......... .......... A......... .......... .......... .......... .......... ..........
Bur.simarouba     .......... .......... .......... .......... .......... .......... .......... .......... .....T.... ..........
Breynia           .......... .......... .......... .......... .......... .......... .......... .......... .......... ..........
Phyllanthus       .......... .......... .......... .......... .......... .......... .......... .......... .......... ..........
Hevea             .......... .......... .......... .......... .......... .......... .......... .......... .......... ..........
Acalypha          .......... .......... .......... .......... .......... .......... .......... .......... .......... ..........
Croton            .......... .......... .......... .......... ...T...... .......... .......... ......C... .......... ..........
Euphorbia         .......... .......... .......... .......... .......... .......... .......... .......... .......... ..........
Hura              .......... .......... .......... .......... .......... .......... .......... .......... .......... ..........
Malpighia         .......... .......... .......... .......... .....T.... .......... .......... .......... .T........ ..........
Polygala          .......... .......... .......... .......... .........T .......... .......... .......... .......... ..........
Humulus           .......... .......... .......... .......... .....T.... .......... .......... .......... .T........ ..........
Pilea             .......... .......... .......... .......... .....T.... .......... .A........ .......... .T........ ..........
Hovenia           .C........ .......... .......... .......... .......... .......... .......T.. .......... .......... ..........
Cyn.songaricum    .......... .......... .....A.... .......... A....T.... .......... .......... .......... .T........ ..........
Cyn.coccineum     .......... .......... .......... .......... A....T.... .......... .......... .......... .T........ ..........
Citrullus         ..A....... .......... .......... .......G.. ...T...... .......... .......... .......... ........C. ..........
Melothria         ..A....... .......... .......... .......G.. ...T...... .......... .......... .......... ........C. ..........
Cucurbita         ..A....... .......... .......... .......G.. ...T...... .......... .......... .......... ........C. ....C.....
Lepionurus        .......... .......... .......... .......... .......... .......... .......... .......... .......... ..........
Andromeda         .......... .......... .......... .......... .....T.... .......... .......... .......... .T....T... ..........
Pyrola            .......... .......... .......... .......... .....T.... .......... .......... .......... .T........ ..........
Symplocus         .......... .......... .......... .......... .....T.... .......... .......... .......... ........C. ..........
Diospyros         .......... .......... G......... .......... .....T.... .......... .......... .......... .T........ ..........
Mitrastema        .......... .......... .......... ......T... .......... .......... .......... .......... .T........ ..........
Barringtonia      .......... T......... .......... .......... .......... .......... .......... .......... .......... ..........
Daucus            .......... .......... ........C. .......... .......... .......... .......... .......... .......... ..........
Hydrocotyle       .......... .......... .......... .......... .......... .......... .......... .......... .......... ..........
Helianthus        ..A....... .......... .......... .......... .......... .......... .......... .......... .......... ..........
Alstonia          ..A....... T......... .......... .......... C......... .......... .......... .......... .......... ..........
Vinca             ..A....... T......... .......... .......... C......... .......... .......... .......... .......... ..........
Nerium            ..A....... .......... .......... .......... C......... .......... .......... .......... .......... ..........
Alyxia            ..A....... .......... .......... .......... C......... .......... .......... .......... .......... ..........
Ochrosia          ..A....... .......... .......... .......... C....T.... .......... .......... .......... .......... ..........
Carissa           ..A....... .......... .......... .......... C......... .......... .......... .......... .......... ..........
Frasera           .......... .......... .......... .......... C......... .......... .......... .......... .......... ..........
Coffea            .......... .......... .......... .......... C....T.... .......... .......... .......... .......... ..........
Ixora             .......... .......... .......... .......... C....T.... .......... .......... .......... .......... ..........
Heliotropium      .......... .......... .......... .......... C......... .......... .......... .......... ......T... ..........
Ehretia           .......... .......... .......... .......... C......... .......... .......... .......... ......T... ..........
Borago            .......... .......... .......... .......... C......... .......... .......... .......... ......T... ..........
Ipomoea           .......... .......... .....A.... .......... .....T.... .......... ....T..... .......... .....T.... ..........
Schizanthus       .......... .......... .......... .G........ .....T.... .......... .......... .......... .....T.... ..........
Goetzea           .......... .......... .....T.... .......... .....T.... .......... .......... .......... .....T.... ..........
Protoschwenckia   .......... .......... .....T.... .......... .....T.... .......... .......... .......... .....T.... ..........
Bru.densiflora    .......... .......... .....T.... .......... .....T.... .......... .......... .......... .....T.... ..........
Bru.jamaicensis   .......... .......... .....T.... .......... .....T.... .......... .......... .......... .....T.... ..........
Bru.grandiflora   .......... .......... .....T.... .......... .....T.... .......... .......... .......... .....T.... ..........
Nicotiana         .......... .......... .....T.... .......... .....T.... .......... .......... .......... .....T.... ..........
Atropa            .......... .......... .....T.... .......... .....T.... .......... .......... .......... .....T.... ..........
Anisodus          ---------- ---------- ---------- ---------- ---------- ---------- ---------- ---------- ---------- ----------
Atropanthe        ---------- ---------- ---------- ---------- ---------- ---------- ---------- ---------- ---------- ----------
Phys.orientalis   .......... .......... .....T.... .......... .....T.... .......... .......... .......... .....T.... ..........
Phys.infundib.    ---------- ---------- ---------- ---------- ---------- ---------- ---------- ---------- ---------- ----------
Przewalskia       ---------- ---------- ---------- ---------- ---------- ---------- ---------- ---------- ---------- ----------
Hyos.pusillus     ---------- ---------- ---------- ---------- ---------- ---------- ---------- ---------- ---------- ----------
Hyos.aureus       .......... .......... .....T.... .......... .....T.... .......... .......... .......... .....T.... ..........
Lycium            .......... .......... .....T.... .......... .....T.... .......... .......... .......... .....T.... ..........
Jaborosa          .......... .......... .....T.... .......... .....T.... .......... .......... .......... .....T.... ..........
Exodeconus        .......... .......... .....T.... .......... .....T.... .......... .......... .......... .....T.... ..........
Juanulloa         .......... .......... .....T.... .......... .....T.... .......... .......... .......... .....T.... ..........
Man.officinarum   ---------- ---------- ---------- ---------- ---------- ---------- ---------- ---------- ---------- ----------
Man.autumnalis    ---------- ---------- ---------- ---------- ---------- ---------- ---------- ---------- ---------- ----------
Man.caulescens    ---------- ---------- ---------- ---------- ---------- ---------- ---------- ---------- ---------- ----------
Solanum           .......... .......... .....T.... .......... .....T.... .......... .......... .......... .....T.... ..........
Calceolaria       .......... .......... .......... .......... .......... .......... .......... .......... .......... ....T.....
Drymonia          .......... .......... .......... .......... .......... .......... .......... .......... .......... ....T.....
Nematanthus       .......... .......... .......... .......... .......... .......... .......... .......... .......... ....T.....
Veronica          ..A....... .......... .......... .......... .......... .....C.... .......... .......... .......... ....T.....
Digitalis         .......... .......... .......... .......... .......... .......... ........G. .......... .......... ....T.....
Celsia            .......... .......... .......... .......... .......... .......... .......... .......... .......... ....T.....
Verbascum         --------.. .......... .......... .......... .......... .......... .......... .......... .......... ....T.....
Justicia          ..A....... .......... .......... .......... .......... .......... .......... .......... .......... ..C.T.....
Barleria          .......... .......... .......... .......... .......... .......... .......... .......... .......... ....T.....
Thunbergia        .......... .......... .......... .......... .......... .......... .......... .......... .......... ....T.....
Sanchezia         .......... .......... .......... .......... .......... .......... .......... .......... .......... ....T.....
Catalpa           .......... .......... .......... .......... .......... .......... .......... .......... .......... ....T.....
Campsis           .......... .......... .......... .......... ...T...... .......... .......... .......... .......... ....T.....
Ajuga             .......... .......... ..G....... .......... .......... .......... .......... .......... ........C. ....T.....
Callicarpa        .......... .......... .......... .......... .......... .......... .......... .......... .......... ....T.....
Sesamum           .......... .......... .......... .......... .......... .......... .......... .......... .......... ....T.....
```

```
                 501        511        521        531        541        551        561        571        581        591
                 |          |          |          |          |          |          |          |          |          |
Rhamnus          CGTTCTAGTG ACAGCATTCC CACTTTTATT ATCACTTCCA GTACTGGCAG GGGCAATTAC CATGTTATTA ACCGATCGAA ACTTTAATAC AACCTTTTCT
Asimina          .....C.... .......... .......... .........G .......... .......... .......... .......... .......... ..........
Magnolia         .....C.... .......... .......... .........G .......... .......... .......... .......... .......... ..........
Knema            .....C.... .......... .......... .........G .......... .......... .......... .......... .......... ..........
Myristica        .....C.... .......... .......... .........G .......... .......... .......... .......... .......... ..........
Peperomia        ---------- ---------- ---------- ---------- ---------- --..T..... .......... .......... .......... ..........
Piper            .......... .......... T......... .........T .......... ....T..... .......... .......... .......... ..........
Arisaema         .......... .......... .......... .........G .......... .......... A......... .......... .......... ..........
Zamioculcas      .......... .......... .......... .........G .......... .......... A......... .......... .......... ..........
Xanthosoma       .......... .......... .......... .........G .......... .......... A......... .......... .......... ..........
Philodendron     .......... .......... .......... .........G .......... .......... A......... .......... .......... ..........
Peltandra        .......... .......... .........C .........G .......... .......... A......... .......... .......... ..........
Strelitzia       .......... .......... T......... .........G .......... .......... T......... .......... .......... ..........
Musa             .........T .....C.... T......... .........G .......... .......A.. A......... .......... .......... ..........
Musella          .......... .....C.... T......... .........G .......... .......A.. A......... .......... .......... ..........
Maranta          .......... .......... T......... .........G .......... .......... T......... .......... .......... ..........
Monotagma        .......... .......... T......... .........G .......... .......... T......... .......... .......... ..........
Haumania         .......... .......... T......... .........G .......... .......... T......... .......... .......... ..........
Globba           .......... .......... T....G.... .........G ........T. .......... T......... .......... .......... ........T.
Costus           .......... ..T....... T......... .........G .......... ....T..... T......... .......... .......... ..........
Triticum         .......... .......... T......... .........G ........G. .......... A......... .......... .......... ........T.
Grevillea        .....C.... .......... .......... .........G .......... .......... .......... .......... .......... ..........
Melia            .......... .......... .......... .........G .......... .......... .......... .......... .......... ........T.
Dysoxylum        .......... .......... T......... .........G .......... .......... .......... .......... .......... ..........
Ailanthus        .......... .......... .......... .........G .......... .......... .......... .......... .......... ..........
Toxicodendron      .......... .......... .......... .........G .......... .......... .......... .......... .......... ..........
Rhus             .......... .......... .......... .........G .......... .......... .......... .......... .......... ..........
Bur.sp           .......... .......... .......... .........G .......... .......... .......... .......... .......... ..........
Bur.simarouba    .......... .......... .......... .........G .......... .......... .......... .......... .......... ..........
Breynia          .......... .......... .......... .........G .......... .......... .......... .......... .......... ........T.
Phyllanthus      .......... .......... .......... .........G .......... .......... .......... .......... .......... ........T.
Hevea            .......... .......... .......... .........G .......... .......... .......... .......... .......... ........T.
Acalypha         .......... .......... .......... .........G .......... .......... .......... .......... .......... ........T.
Croton           .......... .......... .......... .........G .......... .......... .......... .......... .......... ..........
Euphorbia        .......... .......... .......... .........G .......... .......... .......... .......... .......... ........T.
Hura             .......... .......... .......... .........G .......... .......... .......... .......... .......... ........T.
Malpighia        .......... .......... .......... .........G .....T.... .......... .......... .......... .......... ........T.
Polygala         .....C.... .......... .......... .........G .......... .......... .......... .......... .......... ..........
Humulus          .......... .....T.... T......... .........G .......... .......... .......... .......... .......... ........T.
Pilea            .......... .....T.... T......... .........G .......... .......... .......... .......... .......... ........T.
Hovenia          .......... .......... .......... .........G .......... .......... .......... .......... .......... ..........
Cyn.songaricum   .......... .......... T......... .........G .......... .......... .......... .......... .......... ........T.
Cyn.coccineum    .......... .......... T......... .........G .......... .......... .......... .......... .......... ........T.
Citrullus        .......... .......... .......... .........G .......... ....T..... .......... .......... .......... ..........
Melothria        .......... .......... .......... .........G .......... .......... .......... .......... .......... ..........
Cucurbita        .......... .......... .......... .........G .......... ....T..... .......... .......... .......... ..........
Lepionurus       .......... .......... .......... .........G .......... .A........ .......... .......... .......... ..........
Andromeda        .......... .......... T......... .........G .......... .......... .......... .......... .......... ........T.
Pyrola           .......... .......... T......... .........G .......... .......... .......... .......... .......... ........T.
Symplocus        .......... .......... .......... .........G .......... .......... .......... .......... .......... ..........
Diospyros        .......... .......... T......... .........G .......... .......... .......... .......... .......... ........T.
Mitrastema       T........A .......... T......... .........G .......... .......... .......... .......... .......... ..........
Barringtonia     .......... .......... .......... .........G .......... .......... .......... .......... .......... ..........
Daucus           .......... .......... .......... .........G .......... .......... .......... .......... .......... ..........
Hydrocotyle      .....C.... .......... .......... .........G .......... .......... .......... .......... .......... ..........
Helianthus       .......... .......... .......... .........G .......... .......... .......... .......... .......... ..........
Alstonia         .......... .......... .......... .........G .......... .......... .......... .......... .......... ..........
Vinca            .......... .......... .......... .........G .......... .......... .......... .......... .......... ..........
Nerium           .......... .......... .......... .........G .......... .......... .......... .......... .......... ..........
Alyxia           .......... .......... .......... .........G .......... .......... .......... .......... .......... ..........
Ochrosia         .......... .......... .......... .........G .......... .......... .......... .......... .......... ..........
Carissa          .......... .......... .......... .........G .......... .......... .......... .......... .......... ..........
Frasera          .......... .......... .......... .........G .......... .......... .......... .......... .......... ..........
Coffea           A...T..... .........T .......... .........G .......... .......... .......... .......... .......... ..........
Ixora            A...T..... .........T .......... .........G .......... .......... .......... .......... .......... ..........
Heliotropium     .......... .......... .......... .........G .......... .......... .......... .......... .......... ..........
Ehretia          .......... .......... .......... .........G .......... .......... .......... .......... .......... ..........
Borago           .......... .......... .......... .........G ........G. .......... .......... .......... .......... ..........
Ipomoea          .......... .......... .......... .........G .......... .......... .......... .......... .......... ..........
Schizanthus      .......... .......... .......... .........G .......... .......... .......... .......... .......... ..........
Goetzea          .......... .......... .......... .........G .......... .......... .......... .......... .......... ..........
Protoschwenckia  .......... .......... .......... .........G .......... .......... .......... .......... .......... ..........
Bru.densiflora   .......... .......... .......... .........G .......... .......... .......... .......... .......... ..........
Bru.jamaicensis  .......... .......... .......... .........G .......... .......... .......... .......... .......... ..........
Bru.grandiflora  .......... .......... .......... .........G .......... ....T..... .......... .......... .......... ..........
Nicotiana        .......... .......... .......... .........G .......... .......... .......... .......... .......... ..........
Atropa           .......... .......... .......... .........G .......... .......... .......... .......... .......... ..........
Anisodus         ---------- ---------- ---------- ---------- ---------- ---------- ---------- ---------- ---------- ----------
Atropanthe       ---------- ---------- ---------- ---------- ---------- ---------- ---------- ---------- ---------- ----------
Phys.orientalis  .......... .......... .......... .........G .......... .......... .......... .......... .......... ..........
Phys.infundib.   ---------- ---------- ---------- ---------- ---------- ---------- ---------- ---------- ---------- ----------
Przewalskia      ---------- ---------- ---------- ---------- ---------- ---------- ---------- ---------- ---------- ----------
Hyos.pusillus    ---------- ---------- ---------- ---------- ---------- ---------- ---------- ---------- ---------- ----------
Hyos.aureus      .......... .......... .......... .........G .......... .......... .......... .......... .......... ..........
Lycium           .......... .......... .......... .........G .......... .......... .......... .......... .......... ..........
Jaborosa         .......... .......... .......... .........G .......... .......... .......... .......... .......... ..........
Exodeconus       .......... .......... .......... .........G .......... .......... .......... .......... .......... ..........
Juanulloa        .......... .......... .......... .........G .......... .......... .......... .......... .......... ..........
Man.officinarum  ---------- ---------- ---------- ---------- ---------- ---------- ---------- ---------- ---------- ----------
Man.autumnalis   ---------- ---------- ---------- ---------- ---------- ---------- ---------- ---------- ---------- ----------
Man.caulescens   ---------- ---------- ---------- ---------- ---------- ---------- ---------- ---------- ---------- ----------
Solanum          .......... .......... .......... .........G .......... .......... .......... .......... .......... ..........
Calceolaria      .......... .......... .......... .........G .......... .......... .......... ..T....... .......... ........T.
Drymonia         .......... .......... .......... .........G .......... .......... .......... ..T....... .......... ........T.
Nematanthus      .......... .......... .......... .........G .......... .......... .......... ..T....... .......... ........T.
Veronica         .......... .......... .......... .........G .......... .......... .......... ..T....... .......... ........T.
Digitalis        .......... .......... .......... .........G .......... .......... .......... ..T....... .......... ........T.
Celsia           .......... .......... .......... .........G .......... .......... .......... ..T....... .......... ........T.
Verbascum        .......... .......... .......... .........G .......... .......... .......... ..T....... .......... ........T.
Justicia         .......... .......... T......... .........G .......... .......... .......... ..T....... .......... ........T.
Barleria         .......... .......... .......... .........G .......... .......... .......... ..T....... .......... ........T.
Thunbergia       .......... .......... .......... .........G .......... .......... .......... ..T....... .......... ........T.
Sanchezia        .......... .......... .......... .........G .......... .......... .......... ..T....... .......... ........T.
Catalpa          .......... .......... .......... .........G .......... .......... .......... ..T....... .......... ........T.
Campsis          .......... .......... .......... .........G .......... .......... .......... ..T....... .......... ........T.
Ajuga            .......... .......... .......... .........G .......... .......... .......... ..T....... .......... ........TC
Callicarpa       .......... .......... T......... .........G .......... .......... .......... ..T....... .......... ........T.
Sesamum          .......... .......... .......... .........G .......... .......... .......... ..T....... .......... ........T.
```

```
                  601        611        621        631        641        651        661        671        681        691
                  |          |          |          |          |          |          |          |          |          |
Rhamnus           GATCCCGCTG GAGGGGGAGA CCCCATATTA TACCAGCATC TCTTTCGGTT TTTCGGT--- ---TTT---- --AAATGGCC CTTT-TCAGA TGAAAATCTG
Asimina           .......... .......... .......... .......... .......... C......... .......... .......... .......... .TC.......
Magnolia          .......... .......... .......... .......... .......... C......... ...---.... ..-------- ----.----- ----------
Knema             .......... .......... .......... .......... .......... C......... .......... .......... .......... .TC.......
Myristica         .......... .......... .......... .......... .......... C......... .......... .......... .......... .TC.......
Peperomia         .....A.... .......... ...A...... .......... .....T.... C......... .....G.... .......... .......T.. .TC.......
Piper             .......... .......... ...A...... .......... .....T.... C......... ...---.... ..-------- ----.----- ----------
Arisaema          .....T.... .......... .......... .......... .......... C......... .......... .......... .......... .TC.......
Zamioculcas       .....T.... .......... .......... .......... .......... C......... .......... .......... .......... .TC.......
Xanthosoma        .....T.... .......... .......... .......... .......... C......... .......... .......... .......... .TC.......
Philodendron      .....T.... .......... .......... .......... .....T.... C......... .......... .......... .......... .TC.......
Peltandra         .....T.... .......... .......... .......... .......... C......... ...---.... ..-------- ----.----- ----------
Strelitzia        .....T.... .......... .......... .......... .......... C......... ...---.... ..-------- ----.----- ----------
Musa              .....T.... .......... ...T...... .......... .......... C......... .......... ..C..A.... .......T.. .TCC....G.
Musella           .....T.... .......... ...T...... .......... .......... C......... .......... ..C..A.... .......T.. .TCC......
Maranta           .....T.... .......... .......... .......... .......... C......... .......... .......... .......... .TC.......
Monotagma         .....T.... .......... .......... .......... .......... C......... .......... .......... .......... .TC.......
Haumania          .....T.... .......... .......... .......... .......... C......... ...---.... ..-------- ----.----- ----------
Globba            .....T.... .......... .......... .......... .....T.... C......TTT A......... .......... .......... ..........
Costus            .....T..A. .......... .......... .......... .......... C......... ...---.... ..-------- ----.----- ----------
Triticum          .....T..A. .......... ...A...... .......... .....T.... C......... ...---.... ..-------- ----.----- ----------
Grevillea         .......... .......... .......... .......... .......... C......... ...---.... ..-------- ----.----- ----------
Melia             .......... .......... ...A...... .......... .....T.... C......... .......... .......... .......... .TC.......
Dysoxylum         .......... .......... .......... .......... .....T.... .......... .......... .......... .......... .TC.......
Ailanthus         .......... .......... .......... .......... .....T.... .......... ...---.... ..-------- ----.----- ----------
Toxicodendron        .......... .......... .......... .......... .....T.... C......... ...---.... ..-------- ----.----- ----------
Rhus              .......... .......... .......... .......... .....T.... C......... .......... .......... .......... .TC.......
Bur.sp            .......... .......... .......... .......... .....T.... .......... .......... .......... .......... .TC.......
Bur.simarouba     .......... .......... .......... .......... .....T.... C......... ...---.... ..-------- ----.----- ----------
Breynia           .......... .......... .......... .......... .A...T.... C......... .......... .......... .......... .TTTC.....
Phyllanthus       .......... .......... .......... .......... .T...T.... C......... .....G.... ..TT...... .......... ..........
Hevea             .......... .......... .......... .......... .....T.... C......... .......... .......... .......... ..........
Acalypha          .......... .......... .......... .......... .....T.... C......... .......... ..C....... .......... .TTTC.....
Croton            .......... .......... ...A...... .......... .....T.... C......TTT TAAA...... .......... ....T..... .TC.......
Euphorbia         .......... .......... .......... .......... .....T.... C......... .......... .......... .......... ..........
Hura              .......... .......... .......... .......... .....T.... C......... .......... .......... .......... AA........
Malpighia         .......... .......... .......... .......... .....T.... C......... .......... .......... .......... ..........
Polygala          ........A. .......... ...A...... .......... .......... C......... .......... .......... .......... .TC.......
Humulus           .......... .......... ...T...... .......... .....T.... C......... ...---.... ..-------- ----.----- ----------
Pilea             .......... .......... ...T...... .......... .....T.... C......... .......... .......... .......... ..........
Hovenia           .......... .......... .......... .......... .......... C......... .......... .......... .......... ..........
Cyn.songaricum    .......... .......... .......... .......... .....T.... C......... ...---.... ..-------- ----.----- ----------
Cyn.coccineum     .......... .......... ...A...... .......... .....T.... C......... .......... .......... .......... .TTG......
Citrullus         .......... .......... .......... .......... .....T.... C......... .......... .......... .......... ..........
Melothria         .......... .......... .......... .......... .....T.... C......... .......... .......... .......... .TC.......
Cucurbita         .......... .......... .......... .......... .......... C......... ...---.... ..-------- ----.----- ----------
Lepionurus        .....T.... .......... .......... .......... ....C..... C......... .......... .....A.... .......... .TC.......
Andromeda         .......... .......... .......... .......... .....T.... C......... ...---.... ..-------- ----.----- ----------
Pyrola            .......... .......... .......... .......... .....T.... C......... .......... .......... .......... .TC.......
Symplocus         .......... .......... .......... .......... .......... C......... .......... .......... .......... .TC.......
Diospyros         .......... .......... .......... .......... .....T.... C......... .......... .......... .......... ..........
Mitrastema        ........A. .......... ...A...... .......... .......... C......... .......... .......... .......G.. .TCC......
Barringtonia      .......... .......... .......... .......... .....T.... C......... .......... .......... .......... .TC.......
Daucus            .......... .......... .......... .......... .......... C.....C... ...---.... ..-------- ----.----- ----------
Hydrocotyle       .......... .......... .......... .......... .......... C......... .......... .......... .......... .TC.......
Helianthus        .......... .......... .......... .......... .......... C......... ...---.... ..-------- ----.----- ----------
Alstonia          .......... .......... .......... .......... .......... C......... .......... .......... .......... .TC.......
Vinca             .......... .......... .......... .......... .......... C......... .......... .......... .......... .TC.......
Nerium            .......... .......... .......... .......... .......... C......... .......... .......... .......... .TC.......
Alyxia            .......... .......... .......... .......... .......... C......... .......... .......... ..A....... .TC.......
Ochrosia          .......... .......... .......... .......... .......... C......... .......... .......... .......... .TC.......
Carissa           .......... .......... .......... .......... .......... C......... ...---.... ..-------- ----.----- ----------
Frasera           .......... .......... .......... .......... .......... C......... ......AAAT GT........ .......... .T........
Coffea            .......... .......... .......... .......... .......... C......... .......... .......... .......... .TC.......
Ixora             .......... .......... .......... .......... .......... C......... .......... .......... .......... .TC.......
Heliotropium      .......... .......... .......... .......... .......... C......... .......... .......... .......... ..........
Ehretia           .......... .......... .......... .......... .......... C......... .......... .......... .......... .TC.......
Borago            .......... .......... .......... .......... .......... C......... ...---.... ..-------- ----.----- ----------
Ipomoea           .......... .......... .......... .......... .......... C......... .......... ..C....... .......... .TC.......
Schizanthus       .......... .......... .......... .......... .....T.... C......... ...---.... ..-------- ----.----- ----------
Goetzea           .......... .......... .......... .......... .....T.... C......... ...---.... ..-------- ----.----- ----------
Protoschwenckia   .......... .......... .......... .......... .....T.... C......... ...---.... ..-------- ----.----- ----------
Bru.densiflora    .......... .......... .......... .......... .....T.... C......... ...---.... ..-------- ----.----- ----------
Bru.jamaicensis   .......... .......... .......... .......... .....T.... C......... .......... .......... .......... ..........
Bru.grandiflora   .......... .......... .......... .......... .....T.... C......... ...---.... ..-------- ----.----- ----------
Nicotiana         .......... .......... .......... .......... .....T.... C......... ...---.... ..-------- ----.----- ----------
Atropa            .......... .......... .......... .......... .....T.... C......... ...---.... ..-------- ----.----- ----------
Anisodus          ---------- ---------- ---------- ------.... .....TT... C......... ...---.... ..-------- ----.----- ----------
Atropanthe        ---------- ---------- ---------- ---------- --------.. C......... ...---.... ..-------- ----.----- ----------
Phys.orientalis   .......... .......... .......... .......... .....T.... C......... .......... .......... .......... .TC.......
Phys.infundib.    ---------- ---------- ---------- ---------- -----TT... C......... .......... .......... .......... .TC.......
Przewalskia       ---------- ---------- ---------- ---------- ---------- -------... ...---.... ..-------- ----.----- ----------
Hyos.pusillus     ---------- ---------- ---------- ------.... .....TT... C......... .......... .......... .......... .TC.......
Hyos.aureus       .......... .......... .......... .......... .....T.... C......... .......... .......... .......... .TC.......
Lycium            .......... .......... .......... .......... .....T.... C......... ...---.... ..-------- ----.----- ----------
Jaborosa          .......... .......... .......... .......... .....T.... C......... ...---.... ..-------- ----.----- ----------
Exodeconus        .......... .......... .......... .......... .....T.... C......... ...---.... ..-------- ----.----- ----------
Juanulloa         .......... .......... .......... .......... .....T.... C......... ...---.... ..-------- ----.----- ----------
Man.officinarum   ---------- ---------- ---------- ------.... .....TT... C......... .......... .......... .......... .TC.......
Man.autumnalis    ---------- ---------- ---------- ---------- ---------- -------... ...---.... ..-------- ----.----- ----------
Man.caulescens    ---------- ---------- ---------- ------.... .....TT... C......... ...---.... ..-------- ----.----- ----------
Solanum           .......... .......... .......... .......... .....T.... C......... ...---.... ..-------- ----.----- ----------
Calceolaria       .....T.... .......... ...A...... .......... .......... C......... .......... .......... .......... .TC.......
Drymonia          .....T.... .......... ...A...... .......... .....T.... C......... .......... .......... .......... ..........
Nematanthus       .....T.... .......... ...A...... .......... .....T.... C......... .......... .......... .......... ..........
Veronica          .....T.... .......... ...A...... .......... .....T.... C......... .....A.... .......... .......... ..........
Digitalis         .....T.... .......... ...A...... .......... .....T.... C......... .......... .......... .......... ..........
Celsia            .....T.... .......... ...A...... .......... .......... C......... .......... .......... .......... .TC.......
Verbascum         .....T.... .......... ...A....G. .......... .......... C......... ...---.... ..-------- ----.----- ----------
Justicia          .....T.... ....A..... ...A...... .......... .......... C......... .....G.... .......... .......... .TC.......
Barleria          .....T.... .......... ...A...... .......... .......... C......... .....G.... .......... .......... .TC.......
Thunbergia        .....T.... .......... ...A...... .......... .......... C......... .....G.... .......... .......... .TC.......
Sanchezia         .....T.... .......... ...T...... .......... .......... C......... .....G.... .......... .......... .TC.......
Catalpa           .....T.... .......... ...A...... .......... .......... C......... .......... .......... .......... .TC.......
Campsis           .....T.... .......... ...A...... ..T....... .......... C......... ...---.... ..-------- ----.----- ----------
Ajuga             .....T.... .......... ...A...... .......... .......... C......... .......... .......... ......A... .TC.......
Callicarpa        .....T.... .......... ...A...... .......... .......... C......... ...---.... ..-------- ----.----- ----------
Sesamum           .....T.... .......... ...A...... .......... .....T.... C......... .......... .......... .......... .TC.......
```

	701	711	721	731	741	751	761	771	781	791
Rhamnus	AATACGCATT	GCGCTATATG	CTGGAACTGT	CTGCTTAATG	GTACTCCTAC	TATGTTCATA	AGTGGTTTTC	TAGTAAAACC	C------CGA	TCTAGTAAAA
Asimina	....	....	....G.....	....	....	....	....	....	....	....
Magnolia	----------	----------	----------	----------	----------	----------	----------	----------	-......---	----------
Knema	....	....	....G.....	....	....	....	....	....C.....	....	....
Myristica	....	....	....G.....	....	....	....	....	....C.....	....	....
Peperomia	....	.T......C.	....G.....	....	....	......A...	....	....C.....	....	....
Piper	----------	----------	----------	----------	----------	----------	----------	----------	-......---	----------
Arisaema	....	....	....G.....	....	....	....	....	....	....	......C...
Zamioculcas	....	....	....G.....	....	....	...C......	....	....	....	....
Xanthosoma	....	....	....G.....	....	....	....	....	....	....	....
Philodendron	....	....	....G.....	....	....	....	....	....	....	......C...
Peltandra	----------	----------	----------	----------	----------	----------	----------	----------	-......---	----------
Strelitzia	----------	----------	----------	----------	----------	----------	----------	----------	-......---	----------
Musa	....	....	....G.....	....	....	....	....	....C.....	....	......C...
Musella	....	....	....G.....	....	....	....	.........A	....C.....	.AAACCC...	......C...
Maranta	....	....	....G.....	....	....	....	....	..------..	....	......C...
Monotagma	....	....	....G.....	....	....	....	....	..------..	....	....
Haumania	----------	----------	----------	----------	----------	----------	----------	----------	-......---	----------
Globba	....	....	....G.....	....	....	....	....	....	....	....
Costus	----------	----------	----------	----------	----------	----------	----------	----------	-......---	----------
Triticum	----------	----------	----------	----------	----------	----------	----------	----------	-......---	----------
Grevillea	----------	----------	----------	----------	----------	----------	----------	----------	-......---	----------
Melia	....	....	....G.....	....	....	....	....	....	....	......C...
Dysoxylum	....	....	....G.....	....	A.........	....	....	....	....	....
Ailanthus	----------	----------	----------	----------	----------	----------	----------	----------	-......---	----------
Toxicodendron	----------	----------	----------	----------	----------	----------	----------	----------	-......---	----------
Rhus	....	....	....G.....	....	....	....	....	..------..	....	....
Bur.sp	....	....	....G.....	....	A.........	....	....	....	....	....
Bur.simarouba	----------	----------	----------	----------	----------	----------	----------	----------	-......---	----------
Breynia	....	.....G....	....G.....	....	A.........	....	....	....	....	......C...
Phyllanthus	....	.....G....	....G.....	....	A.........	....	....	....	....	......C...
Hevea	....	.....G....	....G.....	....	....	.....GA...	....	....	....	....
Acalypha	....	....	....G.....	....	....	....	.........A	....C.....	....	......C...
Croton	....	....	..T.G.....	....	....	....	....	....	....	......C...
Euphorbia	....	.....G....	....G.....	....	....	....	....	....	....	....
Hura	....	....	....G.....	....	....	....	....	....	....	....
Malpighia	....	.....G....	....G.....	....	....	....	....	....	....	......C...
Polygala	....	....	....G.....	....	....	....	....	..------..	....	....
Humulus	----------	----------	----------	----------	----------	----------	----------	----------	-......---	----------
Pilea	....	....	....G.....	....	....	.........G	....	....	....	....
Hovenia	....	....	....G.....	....	....	....	....	....	....	....
Cyn.songaricum	----------	----------	----------	----------	----------	----------	----------	----------	-......---	----------
Cyn.coccineum	....	....	....G.....	....	....	....	....	....	....	....
Citrullus	....	....	....G.....	....	....	....	....	....	....	....
Melothria	....	....	....G.....	....	....	....	....	....	....	....
Cucurbita	----------	----------	----------	----------	----------	----------	----------	----------	-......---	----------
Lepionurus	....	....	....G.....	....	....	....	....	....	....	....
Andromeda	----------	----------	----------	----------	----------	----------	----------	----------	-......---	----------
Pyrola	....	....	....G.....	....	....	....	....	....	....	....
Symplocus	....	....	....G.....	....	....	....	....	....	....	....
Diospyros	....	.....G....	....G.....	....	....	....	....	....	....	....
Mitrastema	....	....	....G.....	....	....	....	....	.........T	....	....
Barringtonia	....	....	....G.....	....	....	....	....	....	....	......C...
Daucus	----------	----------	----------	----------	----------	----------	----------	----------	-......---	----------
Hydrocotyle	....	....	....G.....	....	....	....	....	..------..	....	....
Helianthus	----------	----------	----------	----------	----------	----------	----------	----------	-......---	----------
Alstonia	....	....	....G.....	....	....	....	....	..------..	....	....
Vinca	....	....	....G.....	....	....C.....	....	....	..------..	....	....
Nerium	....	....	....G.....	....	....	....	....	..------..	....	....
Alyxia	....	....	....G..A..	....	....	....	....	..------..	....	......C...
Ochrosia	....	....	....G.....	....	....	....	....	..------..	....	....
Carissa	----------	----------	----------	----------	----------	----------	----------	----------	-......---	----------
Frasera	....	..A..G....	....G.T...	....	....	....	....	....	.......T..	....
Coffea	....	....	....G.....	....	....	....	.........T	..------..	....	....
Ixora	....	....	....G.....	....	....	C....C....	.........T	..------..	....	....
Heliotropium	....	....	....G.....	....	....	....	....	....	....	....
Ehretia	....	....	....G.....	....	....	....	....	....	....	....
Borago	----------	----------	----------	----------	----------	----------	----------	----------	-......---	----------
Ipomoea	....	....	....G.....	....	....	....	....	....	....	......C...
Schizanthus	----------	----------	----------	----------	----------	----------	----------	----------	-......---	----------
Goetzea	----------	----------	----------	----------	----------	----------	----------	----------	-......---	----------
Protoschwenckia	----------	----------	----------	----------	----------	----------	----------	----------	-......---	----------
Bru.densiflora	----------	----------	----------	----------	----------	----------	----------	----------	-......---	----------
Bru.jamaicensis	....	....	....G.....	....	....	....	....	....	....	......C...
Bru.grandiflora	----------	----------	----------	----------	----------	----------	----------	----------	-......---	----------
Nicotiana	----------	----------	----------	----------	----------	----------	----------	----------	-......---	----------
Atropa	----------	----------	----------	----------	----------	----------	----------	----------	-......---	----------
Anisodus	----------	----------	----------	----------	----------	----------	----------	----------	-......---	----------
Atropanthe	----------	----------	----------	----------	----------	----------	----------	----------	-......---	----------
Phys.orientalis	....	....	....G.....	....	....	....	....	....	....	....
Phys.infundib.	....	....	....G.....	....	....	....	....	....	....	....
Przewalskia	---.......	....	....G.....	....	....	....	....	....	....	....
Hyos.pusillus	....	....	....G.....	....	....	....	....	....	....	....
Hyos.aureus	....	....	....G.....	....	....	....	....	....	....	....
Lycium	----------	----------	----------	----------	----------	----------	----------	----------	-......---	----------
Jaborosa	----------	----------	----------	----------	----------	----------	----------	----------	-......---	----------
Exodeconus	----------	----------	----------	----------	----------	----------	----------	----------	-......---	----------
Juanulloa	----------	----------	----------	----------	----------	----------	----------	----------	-......---	----------
Man.officinarum	....	....	....G.....	....	....	....	....	....	....	......C...
Man.autumnalis	---------.	....	....G.....	....	....	....	....	....	....	......C...
Man.caulescens	----------	----------	----------	----------	----------	----------	----------	----------	-......---	----------
Solanum	----------	----------	----------	----------	----------	----------	----------	----------	-......---	----------
Calceolaria	....	....	....G.....	....	....	........G.	....	....	....	......C...
Drymonia	....	....	....G.....	....	....	.........G	....	....	....	......C...
Nematanthus	....	....	....G.....	....	....	.........G	....	....	....	....
Veronica	....	....	....G.....	....AAC...	....	.........G	....	....	....	......C...
Digitalis	....	....	....G.....	....AAC...	....	.........G	....	....	....	......C...
Celsia	....	....	....G.T...	....	....	....	....	..------..	....	....
Verbascum	----------	----------	----------	----------	----------	----------	----------	----------	-......---	----------
Justicia	....	.....C....	....G.....	....	.G........	....	....C.....	....	....	......C...
Barleria	....	.....G....	....G.....	....	.G........	....	....	....	....	......C...
Thunbergia	....	.....G....	....G.....	....	.G........	....	....	....	....	......C...
Sanchezia	....	.....G....	....G.....	....AAC...	.G........	....	....	....	....	......C...
Catalpa	....	....	....G.....	....	....	....	....	..------..	....	......C...
Campsis	----------	----------	----------	----------	----------	----------	----------	----------	-......---	----------
Ajuga	....	....	....G.....	....	....	....	....	..------..	....	....
Callicarpa	----------	----------	----------	----------	----------	----------	----------	----------	-......---	----------
Sesamum	....	....	....G.....	....	....	....	.........A	....C.....	....	......C...

```
                  801        811        821        831        841        851        861        871        881        891
                  |          |          |          |          |          |          |          |          |          |
Rhamnus           ATGGAGTATC TAAGACACAA TCAGCAGGTA ACCAACGACA TAAAAGCAGT CTAGTAGGAA CCTCAGAGAC TACACGCGCA ACAACTTATC CTAAATCCTT
Asimina           .......... ..G....... .......... .......... .......... .......... .......... .......... .......... ..........
Magnolia          ---------- ---------- ---------- ---------- ---------- ---------- ---------- ---------- ---------- ----------
Knema             .......... .......... .......... .......... .......... .......... .......... ....T..... .......... ..........
Myristica         .......... .......... .......... .......... .......... .......... .......... ....T..... .......... ..........
Peperomia         .......... .......... .......... .......... .......... .......... .......... .C..T..A.. .......... .A........
Piper             ---------- ---------- ---------- ---------- ---------- ---------- ---------- ---------- ---------- ----------
Arisaema          .......... .......... .......... .......... .......... .......... .......... ....T..... .......... ..........
Zamioculcas       .......... .......... .......... .......... .......... .......... .......... ....T..... .......... ..........
Xanthosoma        .......... .......... .......... .......... .......... .......... .......... ....T..... .......... ..........
Philodendron      .......... .......... .......... .......... .......... .......... .......... ....T..... .......... ..........
Peltandra         ---------- ---------- ---------- ---------- ---------- ---------- ---------- ---------- ---------- ----------
Strelitzia        ---------- ---------- ---------- ---------- ---------- ---------- ---------- ---------- ---------- ----------
Musa              .......... .......... .......... .......... .......... .......... .......... ....T..... .......... ..........
Musella           .......... .......... .......... .......... .......... .......... .......... ....T..... .......... ..........
Maranta           .....C.... .......... .......... .......... .......... .......... .......... .......... .......... ..........
Monotagma         .....C.... .......... .......... .......... .......... .......... .......... .......... .......... ..........
Haumania          ---------- ---------- ---------- ---------- ---------- ---------- ---------- ---------- ---------- ----------
Globba            .......... .......... .......... .......... .......... .......... .......... ....T..... .......... ..........
Costus            ---------- ---------- ---------- ---------- ---------- ---------- ---------- ---------- ---------- ----------
Triticum          ---------- ---------- ---------- ---------- ---------- ---------- ---------- ---------- ---------- ----------
Grevillea         ---------- ---------- ---------- ---------- ---------- ---------- ---------- ---------- ---------- ----------
Melia             .......... .......... .......... .......... .......... .......... .......... ....T..... .......... ..........
Dysoxylum         .......... .......... .......... .......... .......... ........G. .......... ....T..... .......... ..........
Ailanthus         ---------- ---------- ---------- ---------- ---------- ---------- ---------- ---------- ---------- ----------
Toxicodendron       ---------- ---------- ---------- ---------- ---------- ---------- ---------- ---------- ---------- ----------
Rhus              .......... .......... .......... .......... .......... .......... .......... .......... .......... AA........
Bur.sp            .......... .......... .......... .......... .......... ........G. .......... ....T..... .......... ..........
Bur.simarouba     ---------- ---------- ---------- ---------- ---------- ---------- ---------- ---------- ---------- ----------
Breynia           .......... .......... .......... .......... .......T.. .......... .......... ....T..... .......T.T G..TT.....
Phyllanthus       .......... .......... .......... .......... .......T.. .......... .......... ....T..... .......T.T G..TT.....
Hevea             .......... .......... .......... .......... .......... .......... .......... ....T..... .......... ..........
Acalypha          .......... .......... .......... .......... .......... .......... .......... ....T..... .......... ..........
Croton            .......... .......... .......... .......... .......... .......... .......... ....T..... .......... ..........
Euphorbia         .......... .......... .......... .......... .......... .......... .......... ....T...A. .......... ..........
Hura              .......... .......... .......... .......... .......... .......... .......... .......... .......... ..........
Malpighia         .......... .......... .......... .......... .......... .......... .......... ....T..... .......... ..........
Polygala          ....GC.... .......A.. .......... .......... .......... .......... .......... .......... .......... ..........
Humulus           ---------- ---------- ---------- ---------- ---------- ---------- ---------- ---------- ---------- ----------
Pilea             .......... .......... .......... .......... .......... .......... .......... .......... .......... ..........
Hovenia           .......... .......... .......... .......... .......... .......... .......... .......... .......... ..........
Cyn.songaricum    ---------- ---------- ---------- ---------- ---------- ---------- ---------- ---------- ---------- ----------
Cyn.coccineum     .......... .......... .......... .......... .......... .......... .......... ....T..... .......... ..........
Citrullus         .......... .......... .......... .......... ..CT...... .......... .......... ....T..... .......... ..........
Melothria         .......... .......... .......... .......... ..CT...... .......... .......... ....T..... .......... ..........
Cucurbita         ---------- ---------- ---------- ---------- ---------- ---------- ---------- ---------- ---------- ----------
Lepionurus        .......... .......... .......... .......... .......... T......... .......... ....T..... .......... ..........
Andromeda         ---------- ---------- ---------- ---------- ---------- ---------- ---------- ---------- ---------- ----------
Pyrola            .......... .......... .......... .......... .......... .......... .......... .......... .......... ..........
Symplocus         ..A....... .......... .......... .......... .......... .......... .......... .......... .......... ..........
Diospyros         .......... .......... .......... .......... .......... .......... .......... ....T..... .......... ..........
Mitrastema        .......... .......... .......... .......... .......... .......... .......... ....T..... .......... ..........
Barringtonia      .......... .......... .......... .......... .......... .......... .......... .......... .......... ..........
Daucus            ---------- ---------- ---------- ---------- ---------- ---------- ---------- ---------- ---------- ----------
Hydrocotyle       ....GC.... .......... .......... .......... .......... .......... .......... .......... .......... ..........
Helianthus        ---------- ---------- ---------- ---------- ---------- ---------- ---------- ---------- ---------- ----------
Alstonia          ....G..... .......... .......... .......... .......... .......... .......... .......... .......... ..........
Vinca             ....G..... .......... .......... .......... .......... .......... .......... .......... .......... ..........
Nerium            ....G..... .......... .......... .......... .......... .......... .......... .......... .......... ..........
Alyxia            .......... .......... .......... .......... .......... .......... .......... .......... .......... ..........
Ochrosia          ....G..... .......... .......... .......... .......... .......... .......... .......... .......... ..........
Carissa           ---------- ---------- ---------- ---------- ---------- ---------- ---------- ---------- ---------- ----------
Frasera           .......... .......... .......... .......... .......C.. .......... .......... ....T..... .......... .A........
Coffea            ....G..... .......... .......... .......... .......... .......... .......... .......... .......... ..........
Ixora             ....G..... .......... .......... .......... .......... .......... .......... .......... .......... ..........
Heliotropium      .......... .......... .......... .......... .......... .......... .......... .......... .......... ..........
Ehretia           .A........ .......... .......... .......... .......... .......... .......... ....T..... .......... ..........
Borago            ---------- ---------- ---------- ---------- ---------- ---------- ---------- ---------- ---------- ----------
Ipomoea           .......... .......... .......... .......... .......... .......... .......... .......... .......... ..........
Schizanthus       ---------- ---------- ---------- ---------- ---------- ---------- ---------- ---------- ---------- ----------
Goetzea           ---------- ---------- ---------- ---------- ---------- ---------- ---------- ---------- ---------- ----------
Protoschwenckia   ---------- ---------- ---------- ---------- ---------- ---------- ---------- ---------- ---------- ----------
Bru.densiflora    ---------- ---------- ---------- ---------- ---------- ---------- ---------- ---------- ---------- ----------
Bru.jamaicensis   .......... .......... .......... .......... .......... .......... .......... .......... .......... ..........
Bru.grandiflora   ---------- ---------- ---------- ---------- ---------- ---------- ---------- ---------- ---------- ----------
Nicotiana         ---------- ---------- ---------- ---------- ---------- ---------- ---------- ---------- ---------- ----------
Atropa            ---------- ---------- ---------- ---------- ---------- ---------- ---------- ---------- ---------- ----------
Anisodus          ---------- ---------- ---------- ---------- ---------- ---------- ---------- ---------- ---------- ----------
Atropanthe        ---------- ---------- ---------- ---------- ---------- ---------- ---------- ---------- ---------- ----------
Phys.orientalis   .....C.... .......... .......... .......... .......... .......... .......... ....T..... .......... ..........
Phys.infundib.    .....C.... .......... .......... .......... .......... .......... .......... ....T..... .......... ..........
Przewalskia       .....C.... .......... .......... .......... .......... .......... .......... ....T..... .......... ..........
Hyos.pusillus     .....C.... .......... .......... .......... .......... .......... .......... ....T..... .......... ..........
Hyos.aureus       .....C.... .......... .......... .......... .......... .......... .......... ....T..... .......... ..........
Lycium            ---------- ---------- ---------- ---------- ---------- ---------- ---------- ---------- ---------- ----------
Jaborosa          ---------- ---------- ---------- ---------- ---------- ---------- ---------- ---------- ---------- ----------
Exodeconus        ---------- ---------- ---------- ---------- ---------- ---------- ---------- ---------- ---------- ----------
Juanulloa         ---------- ---------- ---------- ---------- ---------- ---------- ---------- ---------- ---------- ----------
Man.officinarum   .....C.... .......... .......... .......... .......... .......... .......... ....T..... .......... ..........
Man.autumnalis    .....C.... .......... .......... .......... .......... .......... .......... ....T..... .......... ..........
Man.caulescens    ---------- ---------- ---------- ---------- ---------- ---------- ---------- ---------- ---------- ----------
Solanum           ---------- ---------- ---------- ---------- ---------- ---------- ---------- ---------- ---------- ----------
Calceolaria       .......... .......... .......... .......... .......... .......... .......... .......... .......... ..........
Drymonia          .......... .......... .......... .......... .......... .......... .......... .......... .......... ..........
Nematanthus       .......... .......... .......... .......... .......... .......... .......... .......... .......... ..........
Veronica          .......... .......... .......... .......... .......... .......... .......... .......... .......... ..........
Digitalis         .......... .......... .......... .......... .......... .......... .......... .......... .......... ..........
Celsia            ....G..... .......... .......... .......... .......... .......... .......... .......... .......... ..........
Verbascum         ---------- ---------- ---------- ---------- ---------- ---------- ---------- ---------- ---------- ----------
Justicia          .......... .......... .......... .......... .......... .......... .......... ....T..... .G........ ..........
Barleria          .......... .......... .......... .......... .......... .......... .......... ....T..... .......... ..........
Thunbergia        .......... .......... .......... .......... .......... .......... .......... ....T..... .......... .A........
Sanchezia         .......... .......... .......... .......... .......... .......... .......... ....T..... .......... .GTC......
Catalpa           ....G..... .......... .......... .......... .......... .......... .......... .......... .......... ..........
Campsis           ---------- ---------- ---------- ---------- ---------- ---------- ---------- ---------- ---------- ----------
Ajuga             ....G..... .......... .......... .......... .......... .......... .......... .......... .......C.. ..........
Callicarpa        ---------- ---------- ---------- ---------- ---------- ---------- ---------- ---------- ---------- ----------
Sesamum           .......... .......... .......... .......... .......... .......... .......... ....T..... .......... ..........
```

```
                  901        911        921        931        941        951        961        971        981        991
                  |          |          |          |          |          |          |          |          |          |
Rhamnus           CTGTGAGTGG CTAGCTGGAA TTATCGATGG TGATGGAACT -CTTCAAGTT AGTAAACAAG GATATACTTC TCTTGAAATT ACTATGGGAC TTGAA-----
Asimina           .......... .......... .......... ........G. .A........ .......... .......... .......... .......... ..........
Magnolia          ---------- ---------- ---------- ---------- .--------- ---------- ---------- ---------- ---------- -----.....
Knema             .......... .......... .......... ........G. .A........ .......... .......... .......... .......... ..........
Myristica         .......... .......... .......... ........G. .A........ .......... .......... .......... .......... ..........
Peperomia         .......... .....A.... .......... ........G. .......... .....CA... .........T ...G.....G ........C. .C........
Piper             ---------- ---------- ---------- ---------- .--------- ---------- ---------- ---------- ---------- -----.....
Arisaema          .......... .......... .......... ........G. .A........ ......A... .......... .........G .......... ..........
Zamioculcas       .......... .......... .......... ........G. .A........ ......A... .......... .......... .......... ..........
Xanthosoma        .......... .......... .......... ........G. .A........ .....CA... ......T... .......... .......... ..........
Philodendron      .......... .......... .......... ........G. .A........ .....CA... .......... .......... .......... ..........
Peltandra         ---------- ---------- ---------- ---------- .--------- ---------- ---------- ---------- ---------- -----.....
Strelitzia        ---------- ---------- ---------- ---------- .--------- ---------- ---------- ---------- ---------- -----.....
Musa              .......... .....A.... ..C....... ..C.....G. .A........ .......... ...C...... ...G...... .......... ...C......
Musella           .......... .......... .......... ........G. .A........ .......... .......... ....TC.... .......... ..TC......
Maranta           .......... .......... .......... ........G. .A.GA....G ...C..A... .......... .......... .......... ..........
Monotagma         .......... .......... ..C....... ........G. .A........ ......A... .......... .......... .......... ..........
Haumania          ---------- ---------- ---------- ---------- .--------- ---------- ---------- ---------- ---------- -----.....
Globba            .......... .......... .......... ........G. .A..A..A.. .......... .......... .......... .......... ..........
Costus            ---------- ---------- ---------- ---------- .--------- ---------- ---------- ---------- ---------- -----.....
Triticum          ---------- ---------- ---------- ---------- .--------- ---------- ---------- ---------- ---------- -----.....
Grevillea         ---------- ---------- ---------- ---------- .--------- ---------- ---------- ---------- ---------- -----.....
Melia             .......... .......... .......... ........G. .A........ .......... .......... .......... .......... ..........
Dysoxylum         .......... .......... .......... ........G. .A........ .......... .......... .......... ....C..... ..........
Ailanthus         ---------- ---------- ---------- ---------- .--------- ---------- ---------- ---------- ---------- -----.....
Toxicodendron       ---------- ---------- ---------- ---------- .--------- ---------- ---------- ---------- ---------- -----.....
Rhus              .......... .......... .......... ........G. .A........ ...C..A.G. .......... .......... .......... ..........
Bur.sp            .......... .......... .......... ........G. .A........ .......... ......T... .......... ....C..... ..........
Bur.simarouba     ---------- ---------- ---------- ---------- .--------- ---------- ---------- ---------- ---------- -----.....
Breynia           .A........ .......... .......... ........G. .A.GA..... T......... .......... .......... .......... ..........
Phyllanthus       .......... .......... .......... ........G. .A.GA..... T......... .......... .......... .......... ..........
Hevea             .......... .......... .......... ........G. .A........ .......... .......... .......... .....A.... ..........
Acalypha          .......... .......... ..C....... ........G. .......... ......A... .....G.... .......... .......... ..TC......
Croton            .......... .......... ..C....... ........G. .A........ .......... ....G..... .......... .......... .....GTGAA
Euphorbia         .......... .......... .......... ........G. .A.GA..... .......... .......... .......... .......... ..........
Hura              .......... .......... .......... .......... .......... .......... .......... .......... .......... ..........
Malpighia         .......... .......... .......... ........G. .A........ .......... .......... .A........ .......... ..........
Polygala          .......... .......... .......... ........G. .A........ ......A.G. .......... .......... .......... ...C......
Humulus           ---------- ---------- ---------- ---------- .--------- ---------- ---------- ---------- ---------- -----.....
Pilea             .........A .......... ..C....... .......... .A........ .......... .......... .......... .......... ..........
Hovenia           .......... .......... .......... .......... .......... .......... .......... .......... .......... ..........
Cyn.songaricum    ---------- ---------- ---------- ---------- .--------- ---------- ---------- ---------- ---------- -----.....
Cyn.coccineum     .......... .......... .......... ........G. .A..A..... .......C.. .......... .......... .......A.. ..........
Citrullus         .......... .......... .......C.. ........G. .A........ .......... .......... .......... .......... ..........
Melothria         .......... .......... .......C.. ........G. .A........ .......... .......... .......... .......... ..........
Cucurbita         ---------- ---------- ---------- ---------- .--------- ---------- ---------- ---------- ---------- -----.....
Lepionurus        .......... .......... .......... ........G. .A........ .......... .......... .......... .......... ..........
Andromeda         ---------- ---------- ---------- ---------- .--------- ---------- ---------- ---------- ---------- -----.....
Pyrola            .......... .......... .......... .......... .A........ .......... .......... .........G .......... ..........
Symplocus         .......... .......... ..C....... .......... .A........ .......... .......... .......... .......... ..........
Diospyros         .......... .......... .......... ........G. .A........ .......... .......... .......... .......... ..........
Mitrastema        .......... .......... .......... ........G. .A........ .......... .......... .......... .......... ..........
Barringtonia      .......... .......... .......... .......... .A........ .......... .......... .......... .......... ..........
Daucus            ---------- ---------- ---------- ---------- .--------- ---------- ---------- ---------- ---------- -----.....
Hydrocotyle       .......... .......... .......... ........G. .A........ ......A.G. .......... .......... .......... ..........
Helianthus        ---------- ---------- ---------- ---------- .--------- ---------- ---------- ---------- ---------- -----.....
Alstonia          .......... .......... .......... ........G. .A........ ......A... .......... .......... .......... ..........
Vinca             ...G...... .......... .......... ........G. .T........ ......A... .......... .......... .......... ..........
Nerium            .......... .......... ..C....... ........G. .A........ ......A... ....C..... .......... .......... ..........
Alyxia            .......... .......... .......... ........G. .A........ ......A... .......... .......... .......... ..........
Ochrosia          .......... .......... .......... ........G. .A........ ......A... .......... .......... .......... ..........
Carissa           ---------- ---------- ---------- ---------- .--------- ---------- ---------- ---------- ---------- -----.....
Frasera           ..A....... .......... .......... ........G. .A........ .......... .......... .......... .......... ..........
Coffea            ......A... .......... .......... ........G. .A........ ......A... ....C..... .......... .......... ..........
Ixora             .......... .......... .......... ........G. .A........ ......A... ....C..... .......... .......... ..........
Heliotropium      .......... .......... ..C....... .......... .......... .......... .......... .......... .......... ..........
Ehretia           .......... .......... .......... ........G. .A........ .......... .......... .......... .......... ..........
Borago            ---------- ---------- ---------- ---------- .--------- ---------- ---------- ---------- ---------- -----.....
Ipomoea           ....T..... .......... .......... .......... .......... .......... .......... ....T..... .......... .C........
Schizanthus       ---------- ---------- ---------- ---------- .--------- ---------- ---------- ---------- ---------- -----.....
Goetzea           ---------- ---------- ---------- ---------- .--------- ---------- ---------- ---------- ---------- -----.....
Protoschwenckia   ---------- ---------- ---------- ---------- .--------- ---------- ---------- ---------- ---------- -----.....
Bru.densiflora    ---------- ---------- ---------- ---------- .--------- ---------- ---------- ---------- ---------- -----.....
Bru.jamaicensis   .......... .......... .......... .......... C-........ .......... .......... .......... .......... ..........
Bru.grandiflora   ---------- ---------- ---------- ---------- .--------- ---------- ---------- ---------- ---------- -----.....
Nicotiana         ---------- ---------- ---------- ---------- .--------- ---------- ---------- ---------- ---------- -----.....
Atropa            ---------- ---------- ---------- ---------- .--------- ---------- ---------- ---------- ---------- -----.....
Anisodus          ---------- ---------- ---------- ---------- .--------- ---------- ---------- ---------- ---------- -----.....
Atropanthe        ---------- ---------- ---------- ---------- .--------- ---------- ---------- ---------- ---------- -----.....
Phys.orientalis   .......... .......... .......... ........G. .A........ .......... .......... .......... .......... ..........
Phys.infundib.    .......... .......... .......... ........G. .A........ .......... .......... .......... .......... ..........
Przewalskia       .......... .......... .......... ........G. .A........ .......... .......... .......... .......... ..........
Hyos.pusillus     .......... .......... .......... ........G. .A........ .......... .......... .......... .......... ..........
Hyos.aureus       .......... .......... .......... ........G. .A........ .......... .......... .......... .......... ..........
Lycium            ---------- ---------- ---------- ---------- .--------- ---------- ---------- ---------- ---------- -----.....
Jaborosa          ---------- ---------- ---------- ---------- .--------- ---------- ---------- ---------- ---------- -----.....
Exodeconus        ---------- ---------- ---------- ---------- .--------- ---------- ---------- ---------- ---------- -----.....
Juanulloa         ---------- ---------- ---------- ---------- .--------- ---------- ---------- ---------- ---------- -----.....
Man.officinarum   .C........ .......... .......... ........G. .A........ .......... .......... .......... .......... ..........
Man.autumnalis    .C........ .......... .......... ........G. .A........ .......... .......... .......... .......... ..........
Man.caulescens    ---------- ---------- ---------- ---------- .--------- ---------- ---------- ---------- ---------- -----.....
Solanum           ---------- ---------- ---------- ---------- .--------- ---------- ---------- ---------- ---------- -----.....
Calceolaria       .......... .......... .......... .......... .......... .......... .......... .......... T......... ...C......
Drymonia          .......... .......... .......... .......... .A........ .......... .......... .......... .......... ..........
Nematanthus       .......... .......... .......... .......... .A........ .......... .......... .......... .......... ..........
Veronica          .......... .......... .......... .......... .A........ .......... .......... .......... .......... ..........
Digitalis         .......... .......... .......... .......... .A.......G .......... .......... .......... .......... ..........
Celsia            .......... .......... .......... ........G. .A........ ......A... .......... .......... .......... ..........
Verbascum         ---------- ---------- ---------- ---------- .--------- ---------- ---------- ---------- ---------- -----.....
Justicia          T......... ..G....... .......... ........G. .A.GA..... ......G... .......... ....TC.... .......... ..........
Barleria          .......... .......... .......... ........G. .A.GA..... .......... .......... .......... .......... ..........
Thunbergia        .......... .......... .......... ........G. .A........ .......... .......... .......... .......... ..........
Sanchezia         .......... .......... ..C....... ........G. .A.GA..... .......... .......... .......... .......... ..........
Catalpa           .......... .......... .......... ........G. .A........ ......A... .......... .......... .......... ..........
Campsis           ---------- ---------- ---------- ---------- .--------- ---------- ---------- ---------- ---------- -----.....
Ajuga             .......... .......... .......... ........G. .A........ ......A... .......... .......... .......... ..........
Callicarpa        ---------- ---------- ---------- ---------- .--------- ---------- ---------- ---------- ---------- -----.....
Sesamum           .......... .......... .......... ........G. .......... .......... .....G.... .......... .......... ..........
```

```
                  1001       1011       1021       1031       1041       1051       1061       1071       1081       1091
                  |          |          |          |          |          |          |          |          |          |
Rhamnus           GATCTACCAT TACTACGATA TATCCAACAT ---ATGCTTG GTGGAAGTAT TAAA------ ---ATGCGAT CAGGT----- -GTTAAAGCT TATCGTTATC
Asimina           .......G.C .......... .......... .......... .......... .......... .......... .......... ..C....... ..........
Magnolia          ---------- ---------- ---------- ...------- ---------- ----...... ...------- -----..... .--------- ----------
Knema             .......G.C .......... .......... .......... .......... .......... .......... .......... ..C....... ..........
Myristica         .......G.C .......... .......... .......... .......... .......... .......... .......... ..C....... ..........
Peperomia         ..G....G.C .......... .........A CAAT..GAGT .......... .......... .......... .......... ..C....... ..........
Piper             ---------- ---------- ---------- ...------- ---------- ----...... ...------- -----..... .--------- ----------
Arisaema          .......G.C .......... .......... .......... .......... .......... .......... .......... ..C....... ..........
Zamioculcas       .......G.C .......... .......... .......... .......... .......... .......... .......... ..C....... ..........
Xanthosoma        .......G.C .......... .......... .......... .......... .......... .......... .......... ..C....... ..........
Philodendron      .......G.C .......... .......... .......... .......... .......... .......... .......... ..C....... ..........
Peltandra         ---------- ---------- ---------- ...------- ---------- ----...... ...------- -----..... .--------- ----------
Strelitzia        ---------- ---------- ---------- ...------- ---------- ----...... ...------- -----..... .--------- ----------
Musa              .......G.C .......... .......... .......... .......... C......... .....T...G .......... ..C....... ..........
Musella           .......G.C .......... .......... .......... .......... G......... .......... .......... ..C....... ..........
Maranta           .........C .......... .......... .......... ........C. .......... .........G .......... ..C......A AC........
Monotagma         .........C .......... .......... .......... ........C. .......... .........G .......... ..C....... ..........
Haumania          ---------- ---------- ---------- ...------- ---------- ----...... ...------- -----..... .--------- ----------
Globba            .........C .......... .......... .......... .......... .......... .......... .......... ..C....... ..........
Costus            ---------- ---------- ---------- ...------- ---------- ----...... ...------- -----..... .--------- ----------
Triticum          ---------- ---------- ---------- ...------- ---------- ----...... ...------- -----..... .--------- ----------
Grevillea         ---------- ---------- ---------- ...------- ---------- ----...... ...------- -----..... .--------- ----------
Melia             .......G.C .......... .......... .......... .......... .......... .......... .......... ..CGTC.... ..........
Dysoxylum         .......G.C .......... .......... .......... .......... .......... .......... .......... ..C....... ..........
Ailanthus         ---------- ---------- ---------- ...------- ---------- ----...... ...------- -----..... .--------- ----------
Toxicodendron       ---------- ---------- ---------- ...------- ---------- ----...... ...------- -----..... .--------- ----------
Rhus              .........C .......... .......... .......... .......... C......... .........G .......... ..C....... ..........
Bur.sp            .......G.C .......... .......... .......... .......... .......... .......... .......... ..C....... ..........
Bur.simarouba     ---------- ---------- ---------- ...------- ---------- ----...... ...------- -----..... .--------- ----------
Breynia           .........C .......... .......... .......... .......... .......... .......... .......... ..C....... ..........
Phyllanthus       .........C .......... .......... .......... .......... .TTT...... .......... .......... ..C....... ..........
Hevea             .........C .......... .......... .......... .......... .......... .......... .......... ..C....... ..........
Acalypha          .........C .......... .......... .......... .......... .C........ .......... .......... ..C.C..... ..........
Croton            .......G.C .......... .......--- ...---.... ........C. ....ATAAAA .......... .....AAGCT T.C....... ..........
Euphorbia         .........C .......... ...T...... .........T .......... ....ATAAAA AAA....... .......... ..C....... ...------.
Hura              .........C .......... .......... .......... .......... .......... .......... .......... ..C....... ..........
Malpighia         .........C .......... .......... .......... .......... .......... .......... .......... ..C....... ..........
Polygala          .........C .......... .......... .......... ........C. .......... .........G .......... ..C....... ..........
Humulus           ---------- ---------- ---------- ...------- ---------- ----...... ...------- -----..... .--------- ----------
Pilea             .........C .......... .......... .......... .......... .......... ....A..... .......... ..C....... .........A
Hovenia           .......... .......... .......... .......... .......... .......... .......... .......... ..C....... ..........
Cyn.songaricum    ---------- ---------- ---------- ...------- ---------- ----...... ...------- -----..... .--------- ----------
Cyn.coccineum     .......G.C .......... .......... .......... .......... .......... .......... .......... ..C....... ..........
Citrullus         .........C .......... .......... .......... .......... .......... .......... .......... ..C....... ..........
Melothria         .........C .......... .......... .......... .......... G......... .......... .......... ..C....... ..........
Cucurbita         ---------- ---------- ---------- ...------- ---------- ----...... ...------- -----..... .--------- ----------
Lepionurus        .......G.C .......... .......... .......... .......... .......... .......... .......... ..C....... ..........
Andromeda         ---------- ---------- ---------- ...------- ---------- ----...... ...------- -----..... .--------- ----------
Pyrola            .........C ....T..... .......... .......... .......... .......... .......... .......... ..C....... .........A
Symplocus         .........C .......... .......... .......... .......... .......... .......... .......... ..C....... .........A
Diospyros         .......G.C .......... .......... .......... .......... .......... .......... .......... ..C....... ..........
Mitrastema        .......G.C .......... .......... .......... .......... .......... .......... .......... ..C....... ..........
Barringtonia      .........C .......... .......... .......... .......... .......... .......... .......... ..C....... .........A
Daucus            ---------- ---------- ---------- ...------- ---------- ----...... ...------- -----..... .--------- ----------
Hydrocotyle       .........C .......... .......... .......... ........C. .......... .........G .......... ..C....... ..........
Helianthus        ---------- ---------- ---------- ...------- ---------- ----...... ...------- -----..... .--------- ----------
Alstonia          .........C .......... .......... .......... .......... .......... .........G .......... ..C....... ..........
Vinca             .........C .......... .......... .......... .......... .......... .........G .......... ..C....... ..........
Nerium            .........C .......... .......... .......... .......... .......... .........G .......... ..C....... ..........
Alyxia            .........C .......... .......... .......... .......... .......... .........G .......... ..C....... ..........
Ochrosia          .........C .......... .......... .......... .......... .......... .........G .......... ..C....... ..........
Carissa           ---------- ---------- ---------- ...------- ---------- ----...... ...------- -----..... .--------- ----------
Frasera           .........C .......... .......... .......... .......... .......... .......... .......... ..C....... ..........
Coffea            .........C .......... .......... ....C..... .......... .......... .........G .......... ..C....... ..........
Ixora             .........C .......C.. .......... .......... .......... .......... .........G .......... ..C....... ..........
Heliotropium      .........C .......... .......... .......... .......... .......... .......... .......... ..C....... ..........
Ehretia           .......G.C .......... .......... .......... .......... .......... .......... .......... ..C....... ..........
Borago            ---------- ---------- ---------- ...------- ---------- ----...... ...------- -----..... .--------- ----------
Ipomoea           .........C .......... .......... .......... ........C. .......... .......... .......... ..C....... ..........
Schizanthus       ---------- ---------- ---------- ...------- ---------- ----...... ...------- -----..... .--------- ----------
Goetzea           ---------- ---------- ---------- ...------- ---------- ----...... ...------- -----..... .--------- ----------
Protoschwenckia   ---------- ---------- ---------- ...------- ---------- ----...... ...------- -----..... .--------- ----------
Bru.densiflora    ---------- ---------- ---------- ...------- ---------- ----...... ...------- -----..... .--------- ----------
Bru.jamaicensis   .........C .......... .......... .......... .......... .......... .......... .......... ..C....... ..........
Bru.grandiflora   ---------- ---------- ---------- ...------- ---------- ----...... ...------- -----..... .--------- ----------
Nicotiana         ---------- ---------- ---------- ...------- ---------- ----...... ...------- -----..... .--------- ----------
Atropa            ---------- ---------- ---------- ...------- ---------- ----...... ...------- -----..... .--------- ----------
Anisodus          ---------- ---------- ---------- ...------- ---------- ----...... ...------- -----..... .--------- ----------
Atropanthe        ---------- ---------- ---------- ...------- ---------- ----...... ...------- -----..... .--------- ----------
Phys.orientalis   .......G.C .......... .......... .......... ........C. A......... .......... .......... ..C....... ..........
Phys.infundib.    .......G.C .......... .......... .......... ........C. A......... .......... .......... ..C....... ..........
Przewalskia       .......G.C .......... .......... .......... ........C. A......... .......... .......... ..C....... ..........
Hyos.pusillus     .......G.C .......... .......... .......... ........C. A......... .......... .......... ..C....... ..........
Hyos.aureus       .......G.C .......... .......... .......... ........C. A......... .......... .......... ..C....... ..........
Lycium            ---------- ---------- ---------- ...------- ---------- ----...... ...------- -----..... .--------- ----------
Jaborosa          ---------- ---------- ---------- ...------- ---------- ----...... ...------- -----..... .--------- ----------
Exodeconus        ---------- ---------- ---------- ...------- ---------- ----...... ...------- -----..... .--------- ----------
Juanulloa         ---------- ---------- ---------- ...------- ---------- ----...... ...------- -----..... .--------- ----------
Man.officinarum   .......G.C .......... .......... .......... ........C. A......... .......... .......... ..C....... ..........
Man.autumnalis    .......G.C .......... .......... .......... ........C. A......... .......... .......... ..C....... ..........
Man.caulescens    ---------- ---------- ---------- ...------- ---------- ----...... ...------- -----..... .--------- ----------
Solanum           ---------- ---------- ---------- ...------- ---------- ----...... ...------- -----..... .--------- ----------
Calceolaria       .........C .......... .......... .......... .......... .......... .......... .......... ..C....... ..........
Drymonia          .........C .......... .......... .......... .......... .......... .......... .......... ..C....... .........A
Nematanthus       .........C .......... .......... .......... .......... .......... .......... .......... ..C....... .........A
Veronica          .........C .......... .......... .......... .......... G......... .......... .......... ..C....... .........A
Digitalis         .........C .......... .......... .......... .......... .C........ .......... .......... ..C....... .........A
Celsia            .........C .......... .......... .......... .......... .......... .........G .......... ..C....... ..........
Verbascum         ---------- ---------- ---------- ...------- ---------- ----...... ...------- -----..... .--------- ----------
Justicia          .......G.C .......... .......... .......... .......... .......... .......... .......... ..C....... ..........
Barleria          .......G.C .......... .......... .......... .......... .......... .......... .......... ..C....... ..........
Thunbergia        .......G.C .......... .......... .......... .......... .......... .......... .......... ..C....... ..........
Sanchezia         .......G.C .......... .......... .......... .......... .......... .......... .......... ..C....... ..........
Catalpa           .........C .......... .......... .......... .......... .......... .........G .......... ..C....... ..........
Campsis           ---------- ---------- ---------- ...------- ---------- ----...... ...------- -----..... .--------- ----------
Ajuga             .........C .......... .......... .......... .......... G......... .........G .......... ..C....... ..........
Callicarpa        ---------- ---------- ---------- ...------- ---------- ----...... ...------- -----..... .--------- ----------
Sesamum           .........C .......... .......... .......... .......... .......... .......... .......... ..C....... ..........
```

```
                 1101       1111       1121       1131       1141       1151       1161       1171       1181       1191
                 |          |          |          |          |          |          |          |          |          |
Rhamnus          GACTACATAA TCAACTTGGT ATGATTAAAC TAATTAATTG TATTAATGGT CATATTCGAC ATTCAGCACG ACTACTTCAA CAACATCGTG TCTGTTAAGT
Asimina          .......... .A........ ..T..G...A ....G..... .......... .......... .......... .......... .T........ .....C....
Magnolia         ---------- ---------- ---------- ---------- ---------- ---------- ---------- ---------- ---------- ----------
Knema            .......... .......... .......... ....G..... .......... .......... .......... .......... .T........ .....C....
Myristica        .......... .......... .......... ....G..... .......... .......... .......... .........T .T........ .....C....
Peperomia        .......... .A........ .....G.... .......... .......... A......... .......... .......... .T........ ..C.CC....
Piper            ---------- ---------- ---------- ---------- ---------- ---------- ---------- ---------- ---------- ----------
Arisaema         .......... .A........ .........G ....G..... .......... .......... .......... .......... .T........ .....C....
Zamioculcas      .......... .......... .......... .......... .......... .......... .......... .......... .T........ .....C....
Xanthosoma       .......... .A........ .......... ....G..... .......... .......... .......... .......... .T........ .....C....
Philodendron     .......... .......... .......... ....G..... .......... .......... .......... .......... .T........ .....C....
Peltandra        ---------- ---------- ---------- ---------- ---------- ---------- ---------- ---------- ---------- ----------
Strelitzia       ---------- ---------- ---------- ---------- ---------- ---------- ---------- ---------- ---------- ----------
Musa             .......... .......... ......C... ....C...A. .C........ A......... .......... .......... .T........ .....C....
Musella          .......... .......... .....G.... .C...C.... .........G .......... .......... .......... .T........ .....C....
Maranta          .G........ .A........ .........G ....G..... ...A...... ........G. .......... .......... .T........ .....C....
Monotagma        .G........ .......... .........G ....G..... ...A...... ........G. .......... .......... .T........ .....C....
Haumania         ---------- ---------- ---------- ---------- ---------- ---------- ---------- ---------- ---------- ----------
Globba           .......... .A........ .......... ....G..... .......... .......... .......... .......... .T........ .....C....
Costus           ---------- ---------- ---------- ---------- ---------- ---------- ---------- ---------- ---------- ----------
Triticum         ---------- ---------- ---------- ---------- ---------- ---------- ---------- ---------- ---------- ----------
Grevillea        ---------- ---------- ---------- ---------- ---------- ---------- ---------- ---------- ---------- ----------
Melia            .......... .......... .......... ....G..... .......... .......... .......... .......... .T........ .....C....
Dysoxylum        .......... .......... .......... ....G..... .......... .......... .......... .......... .T........ .....C....
Ailanthus        ---------- ---------- ---------- ---------- ---------- ---------- ---------- ---------- ---------- ----------
Toxicodendron      ---------- ---------- ---------- ---------- ---------- ---------- ---------- ---------- ---------- ----------
Rhus             .......... .......... .......... ....A..... .......... A......... .......... .......... .T........ .....C....
Bur.sp           .......... .......... .......... ....G..... .......... .......... .......... .......... .T........ .....C....
Bur.simarouba    ---------- ---------- ---------- ---------- ---------- ---------- ---------- ---------- ---------- ----------
Breynia          .......... .TTTA..... .......... .....C.... .......... .......... .......... .......... .T........ .....C....
Phyllanthus      .......... .A........ ....AAC... .....C.... .......... .......... .......... .......... .T........ .....C....
Hevea            .......... .A........ .......... ....G..... .......... .......... .......... .......... .T........ .....C....
Acalypha         .......... .......... .......... ....G..... .......... .......T.. .......... ...C...... .T........ .....C....
Croton           .......... .......... .....A---- --..G..... .......... .......... .......... .......... .T........ .....C....
Euphorbia        .......... .......... .......... ....G..... .......... .........A .......... .......... .T........ ...T.C....
Hura             .......... .......... .......... ....G..... .......... .......... .......... .......... .T........ .....C....
Malpighia        .......... .......... .......... ....G..... .......... .......... .......... .......... .T........ .....C....
Polygala         .......... .A........ .........G ....G..... ...A...... ........G. .......... .......... .T........ .....C....
Humulus          ---------- ---------- ---------- ---------- ---------- ---------- ---------- ---------- ---------- ----------
Pilea            .......... .A........ .......... ....G..... .......... ...G...... .......... .......... .T........ .T...C....
Hovenia          .......... .......... .......... ....G..... .......... .......... .......... .......... .T........ .....C....
Cyn.songaricum   ---------- ---------- ---------- ---------- ---------- ---------- ---------- ---------- ---------- ----------
Cyn.coccineum    .......... .A........ .......... ....G..... .......... .......... .......... .......... .T........ .....C....
Citrullus        .......... .......... .......... ....G..... .......... .......... .......... .......... .T........ .....C....
Melothria        .......... .A........ .......... .......... .......... .......... .......... .......... .T........ .....C....
Cucurbita        ---------- ---------- ---------- ---------- ---------- ---------- ---------- ---------- ---------- ----------
Lepionurus       .......... .......... .......... ....G..... .......... .......... .......... .......... .T........ .....C....
Andromeda        ---------- ---------- ---------- ---------- ---------- ---------- ---------- ---------- ---------- ----------
Pyrola           .......... .A........ .........A ....G..... .......... ...G...... .......... .......... .T........ .T...C....
Symplocus        .......... .......... .......... ....G..... .......... ...G...... .......... .......... .T........ .T...C....
Diospyros        .......... .......... .....G.... ....G....T .......... .......... .......... .......... .T........ .....C....
Mitrastema       .......... .A........ .......... ....G..... .......... A......... .......... .......... .T........ .....C....
Barringtonia     .......... .A........ .......... .C..G..... .......... ...G...... .......... .......... .T........ .T...C....
Daucus           ---------- ---------- ---------- ---------- ---------- ---------- ---------- ---------- ---------- ----------
Hydrocotyle      .......... .A........ .....G...G ....G..... ...A.T.... ........G. .......... .......... .T........ .....C....
Helianthus       ---------- ---------- ---------- ---------- ---------- ---------- ---------- ---------- ---------- ----------
Alstonia         .......... .......... .......... ....G..... ...A...... .......... .......... .......... .T........ .....C....
Vinca            .......... .......... .......... ....G..... ...A...... .......... .......... .......... .T........ .....C....
Nerium           .......... ...C...... .......... ....G..... ...A...... .......... .....A.... .......... .T........ .....C....
Alyxia           .......... .......... .......... .......... ...A...... .......... .....A.... .......... .T........ .....C....
Ochrosia         .......... .......... .......... ....G..... ...A...... .......... ....C..... .......... .T........ .....C....
Carissa          ---------- ---------- ---------- ---------- ---------- ---------- ---------- ---------- ---------- ----------
Frasera          .......... .......... .......... ....G..... .......... .......... .......... .......... .T........ .....A....
Coffea           .......... ...C...... .......... ....G..... ...A...... .......... .....A.... .......... .T........ .....C....
Ixora            .......... ...C...... .......... ....G..... ...A...... .......... .......... .......... .T........ .....C....
Heliotropium     .......... .......... .......... ....G..... .......... .......... .......... .......... .T........ .....C....
Ehretia          .......... .A........ .......... ....G..... .......... .......... .......... .......... .T........ .....C....
Borago           ---------- ---------- ---------- ---------- ---------- ---------- ---------- ---------- ---------- ----------
Ipomoea          .......... .A........ .....G.... ....G..... .......... .......... ..GA...... .......... .T........ .....C....
Schizanthus      ---------- ---------- ---------- ---------- ---------- ---------- ---------- ---------- ---------- ----------
Goetzea          ---------- ---------- ---------- ---------- ---------- ---------- ---------- ---------- ---------- ----------
Protoschwenckia  ---------- ---------- ---------- ---------- ---------- ---------- ---------- ---------- ---------- ----------
Bru.densiflora   ---------- ---------- ---------- ---------- ---------- ---------- ---------- ---------- ---------- ----------
Bru.jamaicensis  .......... .......... .......... ....G..... .......... .......... .......... .......... .T........ .....C....
Bru.grandiflora  ---------- ---------- ---------- ---------- ---------- ---------- ---------- ---------- ---------- ----------
Nicotiana        ---------- ---------- ---------- ---------- ---------- ---------- ---------- ---------- ---------- ----------
Atropa           ---------- ---------- ---------- ---------- ---------- ---------- ---------- ---------- ---------- ----------
Anisodus         ---------- ---------- ---------- ---------- ---------- ---------- ---------- ---------- ---------- ----------
Atropanthe       ---------- ---------- ---------- ---------- ---------- ---------- ---------- ---------- ---------- ----------
Phys.orientalis  .......... .A........ .......... ....G..... .......... .......... .......... .......... .T........ .....C....
Phys.infundib.   .......... .A........ .......... ....G..... .......... .......... .......... .......... .T........ .....C....
Przewalskia      .......... .A........ .......... ....G..... .......... .......... .......... .......... .T........ .....C....
Hyos.pusillus    .......... .A........ .......... ....G..... .......... .......... .......... .......... .T........ .....C....
Hyos.aureus      .......... .A........ .......... ....G..... .......... .......... .......... .......... .T........ .....C....
Lycium           ---------- ---------- ---------- ---------- ---------- ---------- ---------- ---------- ---------- ----------
Jaborosa         ---------- ---------- ---------- ---------- ---------- ---------- ---------- ---------- ---------- ----------
Exodeconus       ---------- ---------- ---------- ---------- ---------- ---------- ---------- ---------- ---------- ----------
Juanulloa        ---------- ---------- ---------- ---------- ---------- ---------- ---------- ---------- ---------- ----------
Man.officinarum  .......... .A........ .......... ....G..... .......... .......... .......... .......... .T........ .....C....
Man.autumnalis   .......... .A........ .......... ....G..... .......... .......... .......... .......... .T........ .....C....
Man.caulescens   ---------- ---------- ---------- ---------- ---------- ---------- ---------- ---------- ---------- ----------
Solanum          ---------- ---------- ---------- ---------- ---------- ---------- ---------- ---------- ---------- ----------
Calceolaria      .......... .......... .......... ....G..... .......... .......... .......... .......... .T........ .....C....
Drymonia         .......... .A........ .......... ....G..... .......... ...G...... .......... .......... .T........ .T...C....
Nematanthus      .......... .A........ .......... ....G..... .......... ...G...... .......... .......... .T........ .T...C....
Veronica         .......... .A........ .......... ....G..... .......... ...G...... .......... .......... .T........ .T...C....
Digitalis        .......... GA........ .......... ....G..... .......... ...G...... .......... .......... .T........ .T...C....
Celsia           .......... ...C...... .......... ....G..... ...A...... .......... .......... .......... .T........ .....C..A.
Verbascum        ---------- ---------- ---------- ---------- ---------- ---------- ---------- ---------- ---------- ----------
Justicia         .......... .A........ .......... .....C...C .......... .......A.G .......... .......... .T........ .....C....
Barleria         .......... .......... .......... ....G..... .......... .......A.. .......... .......... .T........ .....C....
Thunbergia       .......... .......... .......... ....G..... .......... .......... .......... .......... .T........ .....C....
Sanchezia        .......... .......... .......... ....G..... .......... .......A.. .......... .......... .T........ .....C....
Catalpa          .......... ...C...... .......... ....G..... ...A...... .......... .......... .......... .T........ .....C....
Campsis          ---------- ---------- ---------- ---------- ---------- ---------- ---------- ---------- ---------- ----------
Ajuga            .......... ...C...... .......... ....G..... ...A...... .......... .......... .......... .T........ .....C....
Callicarpa       ---------- ---------- ---------- ---------- ---------- ---------- ---------- ---------- ---------- ----------
Sesamum          .......... .......... .......... ....G..... .......... .......... .......... ...C...... .T........ .....C....
```

```
                  1201       1211       1221       1231       1241       1251       1261       1271       1281       1291
                  |          |          |          |          |          |          |          |          |          |
Rhamnus           ACATGATACC CCTGTAATTC TACCTATTAC ACTAGATCCT CAATCAAATT GGTTTGCAGG ATTCTTTGAT ---------- ---GCTGATG GTACCATTGG
Asimina           ..TG....T. .......... C......... .......G.. .....C.... .......... .......... .......... .......... ..........
Magnolia          ---------- ---------- ---------- ---------- ---------- ---------- ---------- .......... ...------- ----------
Knema             ..TG....T. .........A .......... .......G.. .....C.... .......... .......... .......... .......... ..........
Myristica         ..TG....T. .........A .......... .......G.. .....C.... .......... .......... .......... .......... ..........
Peperomia         ..TG....A. ...T...... ......A... ...TTCAG.. .....C.... .......T.. .......... .......... ........G. ....T.....
Piper             ---------- ---------- ---------- ---------- ---------- ---------- ---------- .......... ...------- ----------
Arisaema          ..TG....T. .......... .......... .......G.. .....C.... .......... .......... .......... .......... ..........
Zamioculcas       ..TG....T. .......... .......... .......G.. .....C.... .......... .......... .......... .......... ..........
Xanthosoma        ..TG....T. .......... .......... .......G.. .....C.... .......... .......... .......... .......... ..........
Philodendron      ..TG....T. .......... .......... .......G.. .....C.... .......... .......... .......... .......... ..........
Peltandra         ---------- ---------- ---------- ---------- ---------- ---------- ---------- .......... ...------- ----------
Strelitzia        ---------- ---------- ---------- ---------- ---------- ---------- ---------- .......... ...------- ----------
Musa              ..T.....T. ...T...... .......... .......G.. .....C.... .......... .......... .......... .......... ..........
Musella           ..T.....T. .....C.... .......... .......G.. .....C.... .......T.. .......... .......... ........A. ..........
Maranta           ..T.....T. ..A....... ........G. .......G.. .......... .......... .......... .......... .......... ........A.
Monotagma         ..T.....T. ..A....... ........G. .......G.. .......... .......... .......... .......... .......... ........A.
Haumania          ---------- ---------- ---------- ---------- ---------- ---------- ---------- .......... ...------- ----------
Globba            ..TG....T. .......... .......... .......G.. .......... .......... .......... .......... .......... ..........
Costus            ---------- ---------- ---------- ---------- ---------- ---------- ---------- .......... ...------- ----------
Triticum          ---------- ---------- ---------- ---------- ---------- ---------- ---------- .......... ...------- ----------
Grevillea         ---------- ---------- ---------- ---------- ---------- ---------- ---------- .......... ...------- ----------
Melia             ..TG....T. .......... .......... .......G.. .....C.... .......... .......... .......... .......... ..........
Dysoxylum         ..TG....T. .......... .......... .......G.. .....C.... .......... .......... .......... .......... ..........
Ailanthus         ---------- ---------- ---------- ---------- ---------- ---------- ---------- .......... ...------- ----------
Toxicodendron       ---------- ---------- ---------- ---------- ---------- ---------- ---------- .......... ...------- ----------
Rhus              ..TG....T. ..C....... G......... .......G.. .......... .......... .......... .......... .......... ........A.
Bur.sp            ..TG....T. .......... ......AAC. .......G.. .....C.... .......... .......... .......... .......... ..........
Bur.simarouba     ---------- ---------- ---------- ---------- ---------- ---------- ---------- .......... ...------- ----------
Breynia           ..TG....T. .......... .......... .......G.. .......... .......... .......... .......... .......... ..........
Phyllanthus       ..TG....T. .........T .......... .......G.. .......... .......... .......... .......... .......... ..........
Hevea             ..TG....T. .......... .......... .......G.. .......... .......... .......... .......... .......... ..........
Acalypha          ..TG....T. .....C.... .......... .......G.. .C........ .......... .......... .......... .......... ..........
Croton            ..TG....T. ...T...... .......... .......G.. .....C.... .......... .......... .......... .......... ..........
Euphorbia         ..TG....T. .......... .......... .......G.. .......... .......... .......... .......... .......... ..........
Hura              ........T. .......... .......... .......G.. .......... .......... .......... .......... .......... ..........
Malpighia         ..TG....T. .......... .......... .......G.. .....C.... .......... .......... .......... .......... ........T.
Polygala          ..T.....T. ..A....... ........G. .......G.. .......... .......... .......... .......... .......... ........A.
Humulus           ---------- ---------- ---------- ---------- ---------- ---------- ---------- .......... ...------- ----------
Pilea             ........T. .......... .......... .......G.. .......... .......... .......... .......... .......... ........T.
Hovenia           ........T. .......... .......... .......G.. .......... .......... .......... .......... .......... ..........
Cyn.songaricum    ---------- ---------- ---------- ---------- ---------- ---------- ---------- .......... ...------- ----------
Cyn.coccineum     ..TG....T. .......... .......... .......G.. .....C.... .......... .......... .......... .......... ..........
Citrullus         ..TG....T. .......... .......... .......G.. .......... .......... .......... .......... .......... ..........
Melothria         ..TG....T. .......... .......... .......G.. .......... .......... .......... .......... .......... ..........
Cucurbita         ---------- ---------- ---------- ---------- ---------- ---------- ---------- .......... ...------- ----------
Lepionurus        ..TG....T. .......... .......... .......G.. .....C.... .......... .......... .......... .......... ..........
Andromeda         ---------- ---------- ---------- ---------- ---------- ---------- ---------- .......... ...------- ----------
Pyrola            ........T. .......... .......... .......G.. .......... .......... .......... .......... .......... ........T.
Symplocus         ........T. .......... .......... .......G.. .....C.... .......... ..C....... .......... .....G.... ........T.
Diospyros         ..TG....TT .......... .C........ .......G.. .......... .......... .......... .......... .......... ..........
Mitrastema        ..TG....T. .......... .......... .......G.. .....C.... ...A...... .......... .......... .....N.... ..........
Barringtonia      ........T. .......... .......... .......G.. .......... .......... .......... .......... .......... ........T.
Daucus            ---------- ---------- ---------- ---------- ---------- ---------- ---------- .......... ...------- ----------
Hydrocotyle       ..T.....T. ..A....... ........G. .......G.. .......... .......... .......... .......... .......... ........A.
Helianthus        ---------- ---------- ---------- ---------- ---------- ---------- ---------- .......... ...------- ----------
Alstonia          ..TG....T. ..C....... ........C. .......G.. .......... .......... .......... .......... .......... ....T...A.
Vinca             ..TG....T. ..C....... ........C. G......G.. .......... .......... .......... .......... .......... ....T...A.
Nerium            ..TG..A.T. ..C....... ........C. ...T...G.. .......... .......... .......... .......... .......... ....T...A.
Alyxia            ..TG..A.T. ..C..C.... ........C. .......G.. .......... .......... .......... .......... .......... ....T...A.
Ochrosia          ..TG....T. ..C....... .......... .......G.. .......... .......... .......... .......... .......... ....T...A.
Carissa           ---------- ---------- ---------- ---------- ---------- ---------- ---------- .......... ...------- ----------
Frasera           .TTG....T. ..C....... .......... .......A.. .......... .......... .......... CAAGGGCCGG GGTA.AAG.. ..........
Coffea            ..TG.GA.T. ..C....... ........C. ...T...G.. .......... .......... .......... .......... .......... ....TC..A.
Ixora             ..TG..A.T. ..C....... ........C. ...T...G.. .......... .......... .......... .......... .......... ....T...A.
Heliotropium      ........T. .......... .......... .......G.. .......... .......... .......... .......... .......... ..........
Ehretia           ..TG....T. .......... .......... .......G.. .....C.... .......... ......C... .......... .......... ..........
Borago            ---------- ---------- ---------- ---------- ---------- ---------- ---------- .......... ...------- ----------
Ipomoea           ....T...T. .......... .......... ....T..G.. .....C.... .......... .......... .......... .......... ..........
Schizanthus       ---------- ---------- ---------- ---------- ---------- ---------- ---------- .......... ...------- ----------
Goetzea           ---------- ---------- ---------- ---------- ---------- ---------- ---------- .......... ...------- ----------
Protoschwenckia   ---------- ---------- ---------- ---------- ---------- ---------- ---------- .......... ...------- ----------
Bru.densiflora    ---------- ---------- ---------- ---------- ---------- ---------- ---------- .......... ...------- ----------
Bru.jamaicensis   ........T. .......... .......... .......G.. .......... .......... .......... .......... .......... ..........
Bru.grandiflora   ---------- ---------- ---------- ---------- ---------- ---------- ---------- .......... ...------- ----------
Nicotiana         ---------- ---------- ---------- ---------- ---------- ---------- ---------- .......... ...------- ----------
Atropa            ---------- ---------- ---------- ---------- ---------- ---------- ---------- .......... ...------- ----------
Anisodus          ---------- ---------- ---------- ---------- ---------- ---------- ---------- .......... ...------- ----------
Atropanthe        ---------- ---------- ---------- ---------- ---------- ---------- ---------- .......... ...------- ----------
Phys.orientalis   ..TG....T. .......... .......... .......G.. .....C.... .......... .......... .......... .......... ..........
Phys.infundib.    ..TG....T. .......... .......... .......G.. .....C.... .......... .......... .......... .......... ..........
Przewalskia       ..TG....T. .......... .......... .......G.. .....C.... .......... .......... .......... .......... ..........
Hyos.pusillus     ..TG....T. .......... .......... .......G.. .....C.... .......... .......... .......... .......... ..........
Hyos.aureus       ..TG....T. .......... .......... .......G.. .....C.... .......... .......... .......... .......... ..........
Lycium            ---------- ---------- ---------- ---------- ---------- ---------- ---------- .......... ...------- ----------
Jaborosa          ---------- ---------- ---------- ---------- ---------- ---------- ---------- .......... ...------- ----------
Exodeconus        ---------- ---------- ---------- ---------- ---------- ---------- ---------- .......... ...------- ----------
Juanulloa         ---------- ---------- ---------- ---------- ---------- ---------- ---------- .......... ...------- ----------
Man.officinarum   ..TG....T. .......... .......... .......G.. .....C.... .......... .......... .......... .......... ..........
Man.autumnalis    ..TG....T. .......... .......... .......G.. .....C.... .......... .......... .......... .......... ..........
Man.caulescens    ---------- ---------- ---------- ---------- ---------- ---------- ---------- .......... ...------- ----------
Solanum           ---------- ---------- ---------- ---------- ---------- ---------- ---------- .......... ...------- ----------
Calceolaria       ........T. .......... .......... .......G.. .......... .......... .......... .......... .......... ..........
Drymonia          ........T. .......... .......... .......G.. .......... .......... .......... .......... ........G. ........T.
Nematanthus       ........T. .......... .......... .......G.. .......... .......... .......... .......... ........G. ........T.
Veronica          ........T. ...A...... .........G .......G.. .......... .......... .......... .......... .......... ........T.
Digitalis         ........T. .......... .........G .......G.. .......... .......... .......... .......... .......... ........T.
Celsia            ..TG..A.T. ..C....... ........C. .......G.. .......... .......... .......... .......... .......... ....T...AC
Verbascum         ---------- ---------- ---------- ---------- ---------- ---------- ---------- .......... ...------- ----------
Justicia          ..TG....T. ...C...... .........G .......G.. .......... .......... .......... .......... .......... ...G....A.
Barleria          ...G....T. .......... .......... ..GTC..G.. .......... .......... .......... .......... .......... ........A.
Thunbergia        ..TG....T. .......... .......... .......G.. .......... .......... .......... .......... .......... ........A.
Sanchezia         ..TG....T. .......... .......... ..GTC..G.. .......... .......... .......... .......... .......... ........A.
Catalpa           ..TG..A.T. ..C....A.. ........C. .......G.. .......... .......... .......... .......... .......... ....T...A.
Campsis           ---------- ---------- ---------- ---------- ---------- ---------- ---------- .......... ...------- ----------
Ajuga             ..TG..A.T. ..C....... ........C. .......G.. .......... .......... .......... .......... .......... ....TC..AC
Callicarpa        ---------- ---------- ---------- ---------- ---------- ---------- ---------- .......... ...------- ----------
Sesamum           ..TG....T. .....C.... .......... .......G.. .C........ .......... .......... .......... .......... ..........
```

```
               1301       1311       1321       1331       1341       1351       1361       1371       1381       1391
               |          |          |          |          |          |          |          |          |          |
Rhamnus        TATCGCTATG AAGCATCAAC TACCTCAACT AAGTATTCGA GTAACTAATA AACTTCTACA A------GAT GTAGAGTCTT AT----AAG- --GTAGTATT
Asimina        .......... .......G.. .......... .......... ....A....C ..T....... .......... .......... T.......A. ..........
Magnolia       ---------- ---------- ---------- ---------- ---------- ---------- -......--- ---------- --....---. ..--------
Knema          .......... .......G.. .......... .......... ....A..... .......... .......... .......... .......... ..........
Myristica      .......... .......G.. .......... .......... ....A..... .......... .......... .......... .......... ..........
Peperomia      .......... .......G.. .......... .......... ....A....C .......... .......... .......... T......G.. ....C..T..
Piper          ---------- ---------- ---------- ---------- ---------- ---------- -......--- ---------- --....---. ..--------
Arisaema       .......... .......G.. .......... .......... ....A..... ...A...... .......... .......... .......... ..........
Zamioculcas    .......... .......G.. .......... .......... ....A..... ...A....A. .......... .......... .......... ..........
Xanthosoma     .......... .......G.. .......... .......... ....A..... .......... .......... .......... .......... ..........
Philodendron   .......... .......G.. .......... .......... ....A..... .......... .......... .......... .......... ..........
Peltandra      ---------- ---------- ---------- ---------- ---------- ---------- -......--- ---------- --....---. ..--------
Strelitzia     ---------- ---------- ---------- ---------- ---------- ---------- -......--- ---------- --....---. ..--------
Musa           .C........ .......G.. .......... .......... ..C.A..... .......... .......... TGT....... .......... ....C.....
Musella        .......... ......GG.. .......... .......... ..C.A....C .......... .......... T......... .......... ....T.....
Maranta        .......... .......G.. .......... .......... ..C....... .......... .......... ..TTCC.... .......... ..........
Monotagma      .......... .......G.. .......... .......... .......... .......... .......... ..TTCC.... .......... ..........
Haumania       ---------- ---------- ---------- ---------- ---------- ---------- -......--- ---------- --....---. ..--------
Globba         .......... ...A..A... .......... .......... .......... .........G .......T.. .......... .......... ..........
Costus         ---------- ---------- ---------- ---------- ---------- ---------- -......--- ---------- --....---. ..--------
Triticum       ---------- ---------- ---------- ---------- ---------- ---------- -......--- ---------- --....---. ..--------
Grevillea      ---------- ---------- ---------- ---------- ---------- ---------- -......--- ---------- --....---. ..--------
Melia          .......... .......G.. .......... .......... ....A..... .......... .......... .......... .......... ..........
Dysoxylum      .......... .......G.. .......... .......... ....A..... .......... .......... .......... .......... ..........
Ailanthus      ---------- ---------- ---------- ---------- ---------- ---------- -......--- ---------- --....---. ..--------
Toxicodendron    ---------- ---------- ---------- ---------- ---------- ---------- -......--- ---------- --....---. ..--------
Rhus           .T........ .......G.. .......... .......... ..C....... .......... .......... .......... .......... ..........
Bur.sp         .......... .......G.. .......... .......... ....A..... .......... .......... ........C. .......... ..........
Bur.simarouba  ---------- ---------- ---------- ---------- ---------- ---------- -......--- ---------- --....---. ..--------
Breynia        .......... ...A...... .......... .......... .......... .......... .......... ......C... .......... ..........
Phyllanthus    .......... ...A...C.. .......... .......... .......... .......... .......... ......C... .......... ..........
Hevea          .......... ...A...... .......... .......... .......... .......... .......... .......... .......... ..........
Acalypha       .......... ...A...G.. .......... .......... .......... .......... .......... .......... .......... ..........
Croton         .......... .......G.. .......... ....C..... ....A..... ..T.....A. .CTAAAA... .......... .......... ..........
Euphorbia      .......... .TAA...G.. .......... .......... .......... .......... .......... .......... .........G GG........
Hura           .......... .......... .......... .......... .......... .......... .......... ..G....... .......... ..........
Malpighia      .......... ...A...G.. .......... .......... .......... .......... .......... .......... .......... ..........
Polygala       .......... .......G.. .......... .......... .......... .......... .......... ..TTCC.... .......... ..........
Humulus        ---------- ---------- ---------- ---------- ---------- ---------- -......--- ---------- --....---. ..--------
Pilea          .......... .......... .......... .......... .......... .......... .......... .......... .......... ....T.....
Hovenia        .......... .......... .......... .......... .......... .......... .......... .......... .......... ..........
Cyn.songaricum ---------- ---------- ---------- ---------- ---------- ---------- -......--- ---------- --....---. ..--------
Cyn.coccineum  ....A..... .......G.. .......... .......A.. ....A..... .......... .......... .......... .......... ..........
Citrullus      .T........ ...A...G.. .......... .......... .......... .......... .......... .......... .......... ..........
Melothria      .T........ ...A...G.. .......... .......... .......... .......... .......... .......... .......... ..........
Cucurbita      ---------- ---------- ---------- ---------- ---------- ---------- -......--- ---------- --....---. ..--------
Lepionurus     .......... .......G.. .......... .......... ....A..... .......... .......... .......... .......... ..........
Andromeda      ---------- ---------- ---------- ---------- ---------- ---------- -......--- ---------- --....---. ..--------
Pyrola         .......... .G........ .......... .......... .......... .......... .......... .......... .......... ....G.....
Symplocus      .......... .......... .......... .......... .......... .......... .......... .......... .......... ....G.....
Diospyros      .......... ...A...G.. .......... .......... .......... ........A. .......... .......... .......... ..........
Mitrastema     .......... .......G.. .......... .......... ..C.A..... .......... .......... .......... .......... ..........
Barringtonia   .......... .......... .......... .......... .......... .......... .......... .......... .......... ..........
Daucus         ---------- ---------- ---------- ---------- ---------- ---------- -......--- ---------- --....---. ..--------
Hydrocotyle    .......... .......G.. .......... .......... .......... .......... .......... ..G....... .......... ..........
Helianthus     ---------- ---------- ---------- ---------- ---------- ---------- -......--- ---------- --....---. ..--------
Alstonia       ...A.T.... ......GG.. .......... .......... .......... .......... .......... .......... .......... ..........
Vinca          ...A...... ......GG.. .......... .......... ........A. .......... .......... .......... .......... ..........
Nerium         ...A...... ......GG.. .......... .......... .......... .......... .......... .......... .......... .....A....
Alyxia         ...A.....A ......GG.. .......... .......... .......... .......... .......... ..G....... .......... ..........
Ochrosia       ...A...... .......G.. .......... .......... .......... .......... .......... .......... .......... ..........
Carissa        ---------- ---------- ---------- ---------- ---------- ---------- -......--- ---------- --....---. ..--------
Frasera        .C..A..... ..TA...T.. .......... .......... .......... .......... .......... .......... T.TTTT.... ..........
Coffea         ...A...... ......GG.. .......... .......... .......... .......... .......... .......... .......... .....A....
Ixora          ...A...... ......GG.. .......... .......... .......... .......... .......... .......... .......... .....A....
Heliotropium   .......... .......... .......... .......AT. .......... .......... C......... ..G....... .......... ..........
Ehretia        .......... .......G.. .......... .......... ....A..... .......... .......... .......... .......... ..........
Borago         ---------- ---------- ---------- ---------- ---------- ---------- -......--- ---------- --....---. ..--------
Ipomoea        .......... .......... .......... .......... ........G. .......... .......... ..G....... .......... ..........
Schizanthus    ---------- ---------- ---------- ---------- ---------- ---------- -......--- ---------- --....---. ..--------
Goetzea        ---------- ---------- ---------- ---------- ---------- ---------- -......--- ---------- --....---. ..--------
Protoschwenckia ---------- ---------- ---------- ---------- ---------- ---------- -......--- ---------- --....---. ..--------
Bru.densiflora ---------- ---------- ---------- ---------- ---------- ---------- -......--- ---------- --....---. ..--------
Bru.jamaicensis .......... .......... .......... .......... .......... .......... .......... ..G....... .......... ..........
Bru.grandiflora ---------- ---------- ---------- ---------- ---------- ---------- -......--- ---------- --....---. ..--------
Nicotiana      ---------- ---------- ---------- ---------- ---------- ---------- -......--- ---------- --....---. ..--------
Atropa         ---------- ---------- ---------- ---------- ---------- ---------- -......--- ---------- --....---. ..--------
Anisodus       ---------- ---------- ---------- ---------- ---------- ---------- -......--- ---------- --....---. ..--------
Atropanthe     ---------- ---------- ---------- ---------- ---------- ---------- -......--- ---------- --....---. ..--------
Phys.orientalis .......... .......G.. .......... .......... ....A..... .......... .......... .......... .......... ..........
Phys.infundib. .......... .......G.. .......... .......... ....A..... .......... .......... .......... .......... ..........
Przewalskia    .......... .......G.. .......... .......... ....A..... .......... .......... .......... .......... ..........
Hyos.pusillus  .......... .......G.. .......... .......... ....A..... .......... .......... .......... .......... ..........
Hyos.aureus    .......... .......G.. .......... .......... ....A..... .......... .......... .......... .......... ..........
Lycium         ---------- ---------- ---------- ---------- ---------- ---------- -......--- ---------- --....---. ..--------
Jaborosa       ---------- ---------- ---------- ---------- ---------- ---------- -......--- ---------- --....---. ..--------
Exodeconus     ---------- ---------- ---------- ---------- ---------- ---------- -......--- ---------- --....---. ..--------
Juanulloa      ---------- ---------- ---------- ---------- ---------- ---------- -......--- ---------- --....---. ..--------
Man.officinarum .......... .......G.. .......... .......... ..C.A..... .......... .......... .......... .......... ..........
Man.autumnalis .......... .......G.. .......... .......... ..C.A..... .......... .......... .......... .......... ..........
Man.caulescens ---------- ---------- ---------- ---------- ---------- ---------- -......--- ---------- --....---. ..--------
Solanum        ---------- ---------- ---------- ---------- ---------- ---------- -......--- ---------- --....---. ..--------
Calceolaria    .......... .......... .......... .......... .......... ...A...... .......... ..G....... .......... ..........
Drymonia       .......... .......... .......... .......... ..C....... .......... .......... .......... .......... ....T.....
Nematanthus    .......... .......... .......... .......... .......... .......... .......... .......... .......... ....T.....
Veronica       G......... .......... .......... .......... ..C....... ...A....G. .......... T......... .......... ....T.....
Digitalis      .......... .......... .......... .......... ..C....... ...A...... .......... .......... .......... ....T.....
Celsia         ...A...... ......GG.. .......... .......... .......... .......... .......... .......... .......... ..........
Verbascum      ---------- ---------- ---------- ---------- ---------- ---------- -......--- ---------- --....---. ..--------
Justicia       ...G...... ...A..GG.. .......... .......... .......... .......... .......... .......... .......... ..........
Barleria       .......... ...A...G.. .......... .......... .......... .......... .......... .......... .......... ..........
Thunbergia     .......... ...A...G.. .......... .......... ..C....... .......... .......... ........C. .......... ..........
Sanchezia      .......... ...A...G.. .......... .......... .......... .......... .......... .......... .......... ..........
Catalpa        ...A...... ......GG.. .......... .......... ...G...... .......... .......... .......... .......... ..........
Campsis        ---------- ---------- ---------- ---------- ---------- ---------- -......--- ---------- --....---. ..--------
Ajuga          ...A...... ......GG.. .......... .......... .......... .......... .......... .......... .......... .......C..
Callicarpa     ---------- ---------- ---------- ---------- ---------- ---------- -......--- ---------- --....---. ..--------
Sesamum        .......... ...A...G.. .......... .......... .......... .......... .......... .......... .......... ..........
```

```
                 1401       1411       1421       1431       1441       1451       1461       1471       1481       1491
                 |          |          |          |          |          |          |          |          |          |
Rhamnus          TGGAGGAAAT ATCTACTTTG ATAGTAGTCA AAATGGTTAC TATCAATGGT CTGTACAAAG TCGAAAAGAT GTTATCATGA TGCTAGATTA CTTTAAATCA
Asimina          .......... .......... .......... .......... .......... .......... .......... .......... .....TC... ..........
Magnolia         ---------- ---------- ---------- ---------- ---------- ---------- ---------- ---------- ---------- ----------
Knema            .......... .......... ........A. .......... .......... .......... .......... .......... .......... ..........
Myristica        .......... .......... .......... .......... .......... .......... .......... .......... .......... ..........
Peperomia        C..T..T... .........T C......... .......... .......... .......... .......... .......... ...GTC.... ..........
Piper            ---------- ---------- ---------- ---------- ---------- ---------- ---------- ---------- ---------- ----------
Arisaema         .......... .......... .......... .......... .......... .......... .......... .......... .......... ..........
Zamioculcas      .......... .......... .......... .......... .......... .......... .......... ........A. .......... ..........
Xanthosoma       .......... .......... .......... .......... .......... .......... .......... .......... .......... ..........
Philodendron     .......... .......... .......... .......... .......... .......... .......... .......... .......... ..........
Peltandra        ---------- ---------- ---------- ---------- ---------- ---------- ---------- ---------- ---------- ----------
Strelitzia       ---------- ---------- ---------- ---------- ---------- ---------- ---------- ---------- ---------- ----------
Musa             .......... .......... .......... .......C.. .......... .......... .......... .......... .......... ..........
Musella          .......... .......... .......... .......... .......... .......... .......... .......... .......... ..........
Maranta          ........C. ..A....... .......... .......... ...A.C.... .......... .......... .......... ...GTC.... ..........
Monotagma        ........C. ..A....... .......... .......... ...A.C.... .......... .......... .......... .......... ..........
Haumania         ---------- ---------- ---------- ---------- ---------- ---------- ---------- ---------- ---------- ----------
Globba           .......... .......... .....T.... .......... .......... .......... .......... .......... .......... ..........
Costus           ---------- ---------- ---------- ---------- ---------- ---------- ---------- ---------- ---------- ----------
Triticum         ---------- ---------- ---------- ---------- ---------- ---------- ---------- ---------- ---------- ----------
Grevillea        ---------- ---------- ---------- ---------- ---------- ---------- ---------- ---------- ---------- ----------
Melia            .......... .......... .......... .......... .......... .......... .......... .......... .......... ..........
Dysoxylum        .......... .......... .......... .......... .......... .......... .......... .......... ...GTC.... ..........
Ailanthus        ---------- ---------- ---------- ---------- ---------- ---------- ---------- ---------- ---------- ----------
Toxicodendron      ---------- ---------- ---------- ---------- ---------- ---------- ---------- ---------- ---------- ----------
Rhus             .......... .......... .......... .......... ....TT.... .......... .......... .......... .......... ..........
Bur.sp           .......... .......... .......... .......... .......... .......... .......... .......... .......... ..........
Bur.simarouba    ---------- ---------- ---------- ---------- ---------- ---------- ---------- ---------- ---------- ----------
Breynia          ........T. .......... ........A. .......... .......... .......... .A........ .......... .......... ..........
Phyllanthus      ........T. .......... .......... .......... .......... .......... .A........ .......... .......... .........T
Hevea            .......... .......... .......... .......... .......... .......... .......... .......... .......... ..........
Acalypha         .......... .......... .......... .......... .......... .......... .A........ .......... .......... ..........
Croton           .......... .-----.... .......... .......... .......... .......... .......... .......... .......--- -.........
Euphorbia        .......... .......... ........A. .......... .......... .......... .......... .......... .......... ..........
Hura             .......... .......... .......... .......... .......... .......... .......... .......... .......... ..........
Malpighia        .......... .......... .......... .......... .......... .......... .......... .......... .......... ..........
Polygala         ........C. ..A....... .......... .......... ...A.C.... .......... .A.......C .......... .......... ........T.
Humulus          ---------- ---------- ---------- ---------- ---------- ---------- ---------- ---------- ---------- ----------
Pilea            .......... .......... .......... .......... .......... .......... .......... .......... .......... ..........
Hovenia          .......... .......... .......... .......... .......... .......... .......... .......... .......... ..........
Cyn.songaricum   ---------- ---------- ---------- ---------- ---------- ---------- ---------- ---------- ---------- ----------
Cyn.coccineum    .......... .......... ........A. .......... .......... .......... .......... .......... .......... ..........
Citrullus        .......... .......... .......... .......... .......... .......... .......... .......... .......... ..........
Melothria        .......... .......... .......... .......... .......... .......... .......... .......... .......... ..........
Cucurbita        ---------- ---------- ---------- ---------- ---------- ---------- ---------- ---------- ---------- ----------
Lepionurus       .......... .......... .......... .......... .......... .......... .......... .......... .......... ..........
Andromeda        ---------- ---------- ---------- ---------- ---------- ---------- ---------- ---------- ---------- ----------
Pyrola           .......... .......... .......... .......... .......... .......... .......... .......... .....T.... ..........
Symplocus        .......... .......... ........A. .......... .......... .......... .......... .......... .......... ..........
Diospyros        .......... .......... .......... .......... .......... .......... .......... .......... .......... ..........
Mitrastema       .......... .......... ........A. .......... .......... .......... .......... .......... .......... ..........
Barringtonia     ........T. .......... .......... .......... .......... .......... .......... .......C.. .......... ..........
Daucus           ---------- ---------- ---------- ---------- ---------- ---------- ---------- ---------- ---------- ----------
Hydrocotyle      ........C. ..A....... ........A. .......... ...A.C.... .......... .A.......C .......... .......... ..........
Helianthus       ---------- ---------- ---------- ---------- ---------- ---------- ---------- ---------- ---------- ----------
Alstonia         ........G. .......... .......... .......... .......... .......... .......... .......... .......... ..........
Vinca            ........G. .......... .......... .......... .......... .......... .......... A......... .......... ..........
Nerium           ........G. C......... .......... .......... .......... .......... .......... .......... .......... ..........
Alyxia           ........G. C......... .......... .......... .......... .......... .......... .......... .......... ..........
Ochrosia         .......... .......... .......... .......... .......... .......... .......... .......... .......... ..........
Carissa          ---------- ---------- ---------- ---------- ---------- ---------- ---------- ---------- ---------- ----------
Frasera          .......--- --........ .......... .......... .......... .......... .......... .......... .......... ...A......
Coffea           ........G. C......... .......... .......... .......... .......... .......... .......... .......... ..........
Ixora            ........G. C......... .......... .......... .......... .......... ....G..... .......... .......... ..........
Heliotropium     .......... .......... .......... .......... .......... .......... .......... .......... .......... ..........
Ehretia          .......... .......... .......... .......... .......... .......... .......... .......... .......... ..........
Borago           ---------- ---------- ---------- ---------- ---------- ---------- ---------- ---------- ---------- ----------
Ipomoea          .......... .......... ........A. .......... .....C.... .......... .......... ..GC.TT.T. ....TTC.G. ....C...A.
Schizanthus      ---------- ---------- ---------- ---------- ---------- ---------- ---------- ---------- ---------- ----------
Goetzea          ---------- ---------- ---------- ---------- ---------- ---------- ---------- ---------- ---------- ----------
Protoschwenckia  ---------- ---------- ---------- ---------- ---------- ---------- ---------- ---------- ---------- ----------
Bru.densiflora   ---------- ---------- ---------- ---------- ---------- ---------- ---------- ---------- ---------- ----------
Bru.jamaicensis  .......... .......... .......... .......... .......... .......... .......... .......... .......... ..........
Bru.grandiflora  ---------- ---------- ---------- ---------- ---------- ---------- ---------- ---------- ---------- ----------
Nicotiana        ---------- ---------- ---------- ---------- ---------- ---------- ---------- ---------- ---------- ----------
Atropa           ---------- ---------- ---------- ---------- ---------- ---------- ---------- ---------- ---------- ----------
Anisodus         ---------- ---------- ---------- ---------- ---------- ---------- ---------- ---------- ---------- ----------
Atropanthe       ---------- ---------- ---------- ---------- ---------- ---------- ---------- ---------- ---------- ----------
Phys.orientalis  .......... .......... .......... .......... .......... .......... .A........ .......... .......... ..........
Phys.infundib.   .......... .......... .......... .......... .......... .......... .......... .......... .......... ..........
Przewalskia      .......... .......... ........A. .......... .......... .......... .......... .......... .......... ..........
Hyos.pusillus    .......... .......... .......... .......... .......... .......... .A........ .......... .......... ..........
Hyos.aureus      .......... .......... .......... .......... .......... .......... .......... .......... .......... ..........
Lycium           ---------- ---------- ---------- ---------- ---------- ---------- ---------- ---------- ---------- ----------
Jaborosa         ---------- ---------- ---------- ---------- ---------- ---------- ---------- ---------- ---------- ----------
Exodeconus       ---------- ---------- ---------- ---------- ---------- ---------- ---------- ---------- ---------- ----------
Juanulloa        ---------- ---------- ---------- ---------- ---------- ---------- ---------- ---------- ---------- ----------
Man.officinarum  .......... .......... .......... .......... .......... .......... .......... .......... .......... ..........
Man.autumnalis   .......... .......... .......... .......... .......... .......... .......... .......... .......... ..........
Man.caulescens   ---------- ---------- ---------- ---------- ---------- ---------- ---------- ---------- ---------- ----------
Solanum          ---------- ---------- ---------- ---------- ---------- ---------- ---------- ---------- ---------- ----------
Calceolaria      ........T. C......... .......... .......... .......... .......... .......... .......... .......... ..........
Drymonia         .......... .......... .......... .......... .......... .......... .......... .......... .......... ..........
Nematanthus      .........A .......... .......... .......... .......... .......... .......... .......... .......... ..........
Veronica         .......... .......... .......... T......... .......... .......... .A........ .......... .......... ..........
Digitalis        .......... .......... .......... .......... .......... .......... .......... .......... .......... ..........
Celsia           ........G. C......... .......... .......... .......... ..A....... .......... .......... .......... ..........
Verbascum        ---------- ---------- ---------- ---------- ---------- ---------- ---------- ---------- ---------- ----------
Justicia         .......... ...C.....T C......... .....C.... ...A.C.... .....G.... ...C...... C......... .......... ..........
Barleria         .......... ...C.....T C......... .......... ...A.C.... .......... .......... .......... ...GTC.... ..........
Thunbergia       .......... ...C...... .......... .......... ...A.C.... .......... .......... .......... ........G. ..........
Sanchezia        .......... ...C.....T C......... .......... ...A.C.... .......... .......... .......... .......... ..........
Catalpa          ........G. C......... .......... .......... .......... ..A....... .A........ .......... .......... ..........
Campsis          ---------- ---------- ---------- ---------- ---------- ---------- ---------- ---------- ---------- ----------
Ajuga            ........G. C......... .......... .......... .......... ..A....... .......... .......... .......... ...G......
Callicarpa       ---------- ---------- ---------- ---------- ---------- ---------- ---------- ---------- ---------- ----------
Sesamum          .......... .......... .......... .......... .......... .......... .......... .......... .......... ..........
```

```
                  1501       1511       1521       1531       1541       1551       1561       1571       1581       1591
                  |          |          |          |          |          |          |          |          |          |
Rhamnus           AGTACTTTCC GAAGTCATAA ATCACGACGA TTCTTCCTTA TTGAGGAATA TTACAGTCTT TATGATCTCA AAGCATTTAA ACCT-----G ACAGT-----
Asimina           .......... .T........ .......... .......... .......... .......... ..C....... .......... ...C.....C ..........
Magnolia          ---------- ---------- ---------- ---------- ---------- ---------- ---------- ---------- ----.....- -----.....
Knema             .......... .......... .......... .......... .......... .......... ..C....... .......... .......... ..........
Myristica         .......... .......... .......... .......... .......... .......... ..C....... .......... .......... ..........
Peperomia         .......... .......... .......... .......... ..TC...... .......... ..C....... ........C. .........T C.........
Piper             ---------- ---------- ---------- ---------- ---------- ---------- ---------- ---------- ----.....- -----.....
Arisaema          .......... .......... .......... .......... .......... ......C... ..C....... .......... .......... ..........
Zamioculcas       .......... .......... .......... .......... ....A..... .......... ..C....... .......... .......... ..........
Xanthosoma        .......... .......... .......... .......... .......... .......... ..C....... .......... .......... ..........
Philodendron      .......... .......... .......... .......... .......... .......... ..C....... .......... .......... ..........
Peltandra         ---------- ---------- ---------- ---------- ---------- ---------- ---------- ---------- ----.....- -----.....
Strelitzia        ---------- ---------- ---------- ---------- ---------- ---------- ---------- ---------- ----.....- -----.....
Musa              ...G...... .....A.... .....T.... .......... .......... .......... ..C....... ........C. .......... ..........
Musella           ...G...... .....A.... .......... .......... .......... .......... ..C....... .......... .......... ..........
Maranta           .......... .......... .......... ..A....... ....A..... .......... ..C....... .......... .......... ..........
Monotagma         .......... .......... .......... ..T....... ....A..... .......... ..C....... .......... .......... ..........
Haumania          ---------- ---------- ---------- ---------- ---------- ---------- ---------- ---------- ----.....- -----.....
Globba            .......... .......... .......... .......A.. .......... .......... ..C....... .......... .......... ..........
Costus            ---------- ---------- ---------- ---------- ---------- ---------- ---------- ---------- ----.....- -----.....
Triticum          ---------- ---------- ---------- ---------- ---------- ---------- ---------- ---------- ----.....- -----.....
Grevillea         ---------- ---------- ---------- ---------- ---------- ---------- ---------- ---------- ----.....- -----.....
Melia             .......... .......... .......... .......... .......... .C........ ..C....... ........C. .......... .A........
Dysoxylum         .......... .......... .......... .......... ..A....... .......... ..C....... .......... .......... ..........
Ailanthus         ---------- ---------- ---------- ---------- ---------- ---------- ---------- ---------- ----.....- -----.....
Toxicodendron       ---------- ---------- ---------- ---------- ---------- ---------- ---------- ---------- ----.....- -----.....
Rhus              .......... .......... .......... ..T....... .......... .....A.... ..C....... .......... .......... ..........
Bur.sp            .......... .......... .......... .......... ..A....... .......... ..C....... .......... .......... ..........
Bur.simarouba     ---------- ---------- ---------- ---------- ---------- ---------- ---------- ---------- ----.....- -----.....
Breynia           .......... .......... .......... .......... ..A....... .......... ..C....... ........C. .......... ..........
Phyllanthus       .......... .......... .......... .......... ..A....... .......... ..C....... ........C. .......... ..........
Hevea             .......... .......... .......... .......... .......... .......... ..C....... .......... .......... ..........
Acalypha          ........T. .......... .......... .......... .......... .......... ..C....... ........C. .......... ..........
Croton            .......... .......... .......T.. .......... .......... .......... ..C....... .......... C.A....... ..........
Euphorbia         .......... .......... .......... .......... .......... .......... ..C....... .......... .......... ..........
Hura              .......... .......... ...G...... .......... .......... .......... ..C....... .......... .......... ..........
Malpighia         .......... .......... .......... .......... .......... .......... ..C....... .......... .......... ..........
Polygala          .......... .......... .......... .......... .......... .......... ..C....... ........C. .......... ..........
Humulus           ---------- ---------- ---------- ---------- ---------- ---------- ---------- ---------- ----.....- -----.....
Pilea             .......... .......... .......... A......... .......... .......... ..C....... .......... .......... ..........
Hovenia           .......... .......... .......... .......... .......... .......... ..C....... .......... .......... ..........
Cyn.songaricum    ---------- ---------- ---------- ---------- ---------- ---------- ---------- ---------- ----.....- -----.....
Cyn.coccineum     .......... .......... .......... .......... .......... .......... ..C....... .......... ...G...... .A........
Citrullus         .......... .......... .......... .......... .......... .......... ..C....... .......... .......... ..........
Melothria         .......... .......... .......... .......... .......... .......... ..C....... .......... .......... ..........
Cucurbita         ---------- ---------- ---------- ---------- ---------- ---------- ---------- ---------- ----.....- -----.....
Lepionurus        .......... .......... .......... .......... .......... .......... ..C....... .......... .......... ..........
Andromeda         ---------- ---------- ---------- ---------- ---------- ---------- ---------- ---------- ----.....- -----.....
Pyrola            .......... .......... .......... .......... .......... .......... ..C....... .......... .T........ ..........
Symplocus         .......... .......... .......... .......... .......... .......... ..C....... .......... .......... ..........
Diospyros         .......... .......... .......... .......... .......... .......... ..C....... .......... .......... ..........
Mitrastema        .......... .......... .......... .......... ..A....... .......... ..C....... .......... .......... ..........
Barringtonia      .......... .......... .......... .......... .......... .......... ..C....... .......... .......... ..........
Daucus            ---------- ---------- ---------- ---------- ---------- ---------- ---------- ---------- ----.....- -----.....
Hydrocotyle       .......... .......... .......... .......... .......... .......... ..C....GA. ........C. .......... ..........
Helianthus        ---------- ---------- ---------- ---------- ---------- ---------- ---------- ---------- ----.....- -----.....
Alstonia          .A........ .......... .......... .......... .......... .......... ..C....... .......... .......... ..........
Vinca             .A........ .......... .......... .......... .......... .......... ..C....... .......... .......... ..........
Nerium            .A........ .......... .......... .......... .......... .......... ..C....... .......... .......... ..........
Alyxia            .A........ .......... .......... .........C .A........ .......... ..C....... .......... .......... C.........
Ochrosia          .A..C..... .......... .......... .......... .......... .......... ..C....... .......... .......... ..........
Carissa           ---------- ---------- ---------- ---------- ---------- ---------- ---------- ---------- ----.....- -----.....
Frasera           .......... .......... .......... .......... .......... .....A.... ..C....... .......... ....GCATGA T....ATAGG
Coffea            .A........ .......... .......... .......... .......... .......... ..C....... .......... .......... ..........
Ixora             .A........ .......... .......... .......... .......... .......... ..C....... .......... .......... ..........
Heliotropium      .......... .......... .......... .......... .......... .......... ..C....... .......... .......... ..........
Ehretia           .......... .......... .......... .......... .......... .......... ..C....... .......... .......... ..........
Borago            ---------- ---------- ---------- ---------- ---------- ---------- ---------- ---------- ----.....- -----.....
Ipomoea           .......GGA .......... .......... .......... .......... .......... ..C....... .......G.. .......... ..........
Schizanthus       ---------- ---------- ---------- ---------- ---------- ---------- ---------- ---------- ----.....- -----.....
Goetzea           ---------- ---------- ---------- ---------- ---------- ---------- ---------- ---------- ----.....- -----.....
Protoschwenckia   ---------- ---------- ---------- ---------- ---------- ---------- ---------- ---------- ----.....- -----.....
Bru.densiflora    ---------- ---------- ---------- ---------- ---------- ---------- ---------- ---------- ----.....- -----.....
Bru.jamaicensis   G......... .......... .......... .......... .......... .......... ..C....... .......... .......... ..........
Bru.grandiflora   ---------- ---------- ---------- ---------- ---------- ---------- ---------- ---------- ----.....- -----.....
Nicotiana         ---------- ---------- ---------- ---------- ---------- ---------- ---------- ---------- ----.....- -----.....
Atropa            ---------- ---------- ---------- ---------- ---------- ---------- ---------- ---------- ----.....- -----.....
Anisodus          ---------- ---------- ---------- ---------- ---------- ---------- ---------- ---------- ----.....- -----.....
Atropanthe        ---------- ---------- ---------- ---------- ---------- ---------- ---------- ---------- ----.....- -----.....
Phys.orientalis   .......... .......... .......... .......... .......... ..T....... ..C....... .......... .......... .A........
Phys.infundib.    .......... .......... .......... .......... .......... ..T....... ..C....... .......... .......... .A........
Przewalskia       .......... .......... .......... .......... .......... ..T....... ..C....... .......... .......... .A........
Hyos.pusillus     .......... .......... .......... .......... .......... ..T....... ..C....... .......... .......... .A........
Hyos.aureus       .......... .......... .......... .......... .......... ..T....... ..C....... .......... .......... .A........
Lycium            ---------- ---------- ---------- ---------- ---------- ---------- ---------- ---------- ----.....- -----.....
Jaborosa          ---------- ---------- ---------- ---------- ---------- ---------- ---------- ---------- ----.....- -----.....
Exodeconus        ---------- ---------- ---------- ---------- ---------- ---------- ---------- ---------- ----.....- -----.....
Juanulloa         ---------- ---------- ---------- ---------- ---------- ---------- ---------- ---------- ----.....- -----.....
Man.officinarum   .......... .......... .......... .......... .......... ..T....... ..C....... .......... .......... .A........
Man.autumnalis    .......... .......... .......... .......... .......... ..T....... ..C....... .......... .......... .A........
Man.caulescens    ---------- ---------- ---------- ---------- ---------- ---------- ---------- ---------- ----.....- -----.....
Solanum           ---------- ---------- ---------- ---------- ---------- ---------- ---------- ---------- ----.....- -----.....
Calceolaria       .......... .......... ...G...... .......... .......... .......... ..C....... .......... .......... ..........
Drymonia          .......... .......... .......... .......AAC ....A..... .......... ..C....... .......... .......... ..........
Nematanthus       .......... .......... .......... .......AAC ....A..... .......... ..C....... .......... .......... ..........
Veronica          .......... ......C... .......... .....T.... ....A..... .......... ..C......C ........C. .........T ..........
Digitalis         .......... .......... .......... .......... ....A..... .......... ..C......C .......... .......... ..........
Celsia            .A........ .......... .......... .......... .......... .......... ..C....... .....----- -......... ..........
Verbascum         ---------- ---------- ---------- ---------- ---------- ---------- ---------- ---------- ----.....- -----.....
Justicia          .......... .......... .......... .......... .......... .......... ..C....... .......... .......... ..........
Barleria          .......... .......... .......... .......... .......... .......... ..C....... .......... .......... ..........
Thunbergia        .......... .......... .......... .......... .......... .......... ..C....... .......... .......... ..........
Sanchezia         .......... .......... .......... .......... .......... .......... ..C....... .......... .......... ..........
Catalpa           .A........ .......... .......... .......... .......... .......... ..C....... .......... .......... ..........
Campsis           ---------- ---------- ---------- ---------- ---------- ---------- ---------- ---------- ----.....- -----.....
Ajuga             .A........ .......... .......... .......... .......... .......... ..C....... .......... .......... ..........
Callicarpa        ---------- ---------- ---------- ---------- ---------- ---------- ---------- ---------- ----.....- -----.....
Sesamum           ........T. .......... .......... .......... .......... .......... ..C....... .......... .......... ..........
```

```
                 1601       1611       1621       1631       1641       1651       1661       1671       1681       1691
                 |          |          |          |          |          |          |          |          |          |
Rhamnus          --------AT TCACCATAAA GCATGGCTAG CTTTCCTAGA CAAATGGAAT -----AAGTT GATGATATAG TCCACCTTTC TTCTATTCAT C----CGCTT
Asimina          .......... .......... .......... .......... .......... .......... .......... ....T..... .......... ..........
Magnolia         ........-- ---------- ---------- ---------- ---------- .....----- ---------- ---------- ---------- -....-----
Knema            .......... .......... .......... .......... .......... .......... .......... .......... .......... ..........
Myristica        .......... .......... .......... .......... .......... .......... .......... .......... .......... ..........
Peperomia        .......... GA........ .......... .......... .......... .......... .......... .......... .......... ..........
Piper            ........-- ---------- ---------- ---------- ---------- .....----- ---------- ---------- ---------- -....-----
Arisaema         .......... .......... .......... .......... .......... .......... .......... .......... .......... ..........
Zamioculcas      .......... .......... .......... .......... .......... .......... .......... .......... .......... ..........
Xanthosoma       .......... .......... .......... .......... .......... .......... .......... .......... ......GA.. ..........
Philodendron     .......... .......... .......... .......... .......... .......... .......... .......... .......... ..........
Peltandra        ........-- ---------- ---------- ---------- ---------- .....----- ---------- ---------- ---------- -....-----
Strelitzia       ........-- ---------- ---------- ---------- ---------- .....----- ---------- ---------- ---------- -....-----
Musa             .......... .......... .......... .......... .......... .......... .......... .......... .......... .CG....A.G
Musella          .......... .......... .......... .......... .......... AGAAT..... .......... .......... .......... .CG....A.G
Maranta          .......... .......... .......... .......... .......... .......... .......... .......... .......... .CGCT.....
Monotagma        .......... .......... .......... .......... .......... .......... .......... .......... .......... .CGCT.....
Haumania         ........-- ---------- ---------- ---------- ---------- .....----- ---------- ---------- ---------- -....-----
Globba           .......... .......... .......... .......... .......... .......... .......... .......... .......... .TT..--...
Costus           ........-- ---------- ---------- ---------- ---------- .....----- ---------- ---------- ---------- -....-----
Triticum         ........-- ---------- ---------- ---------- ---------- .....----- ---------- ---------- ---------- -....-----
Grevillea        ........-- ---------- ---------- ---------- ---------- .....----- ---------- ---------- ---------- -....-----
Melia            .......... .......... .......... .......... .......... .......... .......... .......... .......... ..........
Dysoxylum        .......... .......... .......... .......... .......... .......... .......... .......... .......... ..........
Ailanthus        ........-- ---------- ---------- ---------- ---------- .....----- ---------- ---------- ---------- -....-----
Toxicodendron    ........-- ---------- ---------- ---------- ---------- .....----- ---------- ---------- ---------- -....-----
Rhus             .......... .......... .......... ........A. .......... .......... .......... .......... .......... ......A---
Bur.sp           .......... .......... .......... .......... .......... .......... .......... .......... .......... ..........
Bur.simarouba    ........-- ---------- ---------- ---------- ---------- .....----- ---------- ---------- ---------- -....-----
Breynia          .......... .......... ........T. .......... .......... .......... .......... .......... .......... ..........
Phyllanthus      .......... .......... ........T. .......... .......... .......... .......... .......... .......... ..........
Hevea            .......... .......... .......... .......... .......... .......... .......... .......... .......... ........AA
Acalypha         .......... .......... .......... .......... .......... .......... .......... .......... .......... ..........
Croton           ........C. .......... .......... .......... .........G .......... .......... .......... .......... ..........
Euphorbia        .......... .......... .......... .......... .......... .......... .......... .......... .......... ..........
Hura             .......... .......... .......... .......... .......... .......... .......... .......... .......... ..........
Malpighia        .......... .......... .......... .......... .......... .......... .......... .......... .......... ..........
Polygala         .......... .......... .......... .......... .......... .......... .......... .......... .......... .........C
Humulus          ........-- ---------- ---------- ---------- ---------- .....----- ---------- ---------- ---------- -....-----
Pilea            .......... .......... .......... .......... .......... .......... .......... ...------- ---------- -....A.---
Hovenia          .......... .......... .......... .......... .......... .......... .......... .......... .......... ..........
Cyn.songaricum   ........-- ---------- ---------- ---------- ---------- .....----- ---------- ---------- ---------- -....-----
Cyn.coccineum    .......... .......... .......... .......... G........G .......... .......... .......... .......... ..........
Citrullus        ........C. .......... .......... .......... .......... .......... .......... .......... .......... ..........
Melothria        ........C. .......... .......... .......... .......... .......... .......... .......... .......... ..........
Cucurbita        ........-- ---------- ---------- ---------- ---------- .....----- ---------- ---------- ---------- -....-----
Lepionurus       .......... .......... .......... .......... .......... .......... .......... .......... .......... ..........
Andromeda        ........-- ---------- ---------- ---------- ---------- .....----- ---------- ---------- ---------- -....-----
Pyrola           .......... .......... .......... .....T.... .......... .......... .......... .......... .......... ..........
Symplocus        .......... .......... .......... .......... .......... .......... .......... .......... .......... ..........
Diospyros        .......... .......... .......... .......... .......... .......... .......... .......... .......... ..........
Mitrastema       .......... .......... .......... .......... .......... .......... .......... .......... .......... ..........
Barringtonia     .......... .......... .......... .......... .......... .......... .......... .......... .......A.. ..........
Daucus           ........-- ---------- ---------- ---------- ---------- .....----- ---------- ---------- ---------- -....-----
Hydrocotyle      .......... GA........ .......... .......... .......... .......... .......... .......... .......... .TGCT.....
Helianthus       ........-- ---------- ---------- ---------- ---------- .....----- ---------- ---------- ---------- -....-----
Alstonia         .......... .......... .......... .......... .......... .......... .......... .......... .......... ..........
Vinca            ........C. .......... .......... .......... .......... .......... .......... .......... .......... .....T....
Nerium           .......... .......... .......... .......... T......... .......... .......... .......... .......... ..........
Alyxia           .......... .......... .......... .......... .........A .......... .......... .......... .......... ..........
Ochrosia         .......... .......... .......... .......... .......... .......... .......... .......... .......... ..........
Carissa          ........-- ---------- ---------- ---------- ---------- .....----- ---------- ---------- ---------- -....-----
Frasera          TGTTCTTG.. .......... .......... .......... .......... .......... .......... .......... .......... ..........
Coffea           ........C. .......... .T........ .......... T......... .......... .......... .......... .......... .......---
Ixora            .......... .......... .......... ....T..... T......... .......... .......... .......... .......... .......---
Heliotropium     .......... .......... .......... .......... .......... .......... .......... .......... .......... ..........
Ehretia          .......... .......... .......... .......... .......... .......... .......... .......... .......... ..........
Borago           ........-- ---------- ---------- ---------- ---------- .....----- ---------- ---------- ---------- -....-----
Ipomoea          .......... GA........ .......... .......... .........G .......... .......... .......... .......... ..........
Schizanthus      ........-- ---------- ---------- ---------- ---------- .....----- ---------- ---------- ---------- -....-----
Goetzea          ........-- ---------- ---------- ---------- ---------- .....----- ---------- ---------- ---------- -....-----
Protoschwenckia  ........-- ---------- ---------- ---------- ---------- .....----- ---------- ---------- ---------- -....-----
Bru.densiflora   ........-- ---------- ---------- ---------- ---------- .....----- ---------- ---------- ---------- -....-----
Bru.jamaicensis  .......... .......... .......... .......... .......... .......... .......... .......... .......... ..........
Bru.grandiflora  ........-- ---------- ---------- ---------- ---------- .....----- ---------- ---------- ---------- -....-----
Nicotiana        ........-- ---------- ---------- ---------- ---------- .....----- ---------- ---------- ---------- -....-----
Atropa           ........-- ---------- ---------- ---------- ---------- .....----- ---------- ---------- ---------- -....-----
Anisodus         ........-- ---------- ---------- ---------- ---------- .....----- ---------- ---------- ---------- -....-----
Atropanthe       ........-- ---------- ---------- ---------- ---------- .....----- ---------- ---------- ---------- -....-----
Phys.orientalis  .......... .......... .......... .......... .......... .......... .......... .......... .......... ..........
Phys.infundib.   .......... .......... .......... .......... .......... .......... .......... .......... .......... ..........
Przewalskia      .......... .......... .......... .......... .......... .......... .......... .......... .......... ..........
Hyos.pusillus    .......... .......... .......... .......... .......... .......... .......... .......... .......... ..........
Hyos.aureus      .......... .......... .......... .......... .......... .......... .......... .......... .......... ..........
Lycium           ........-- ---------- ---------- ---------- ---------- .....----- ---------- ---------- ---------- -....-----
Jaborosa         ........-- ---------- ---------- ---------- ---------- .....----- ---------- ---------- ---------- -....-----
Exodeconus       ........-- ---------- ---------- ---------- ---------- .....----- ---------- ---------- ---------- -....-----
Juanulloa        ........-- ---------- ---------- ---------- ---------- .....----- ---------- ---------- ---------- -....-----
Man.officinarum  .......... .......... .......... .......... .......... .......... .......... .......... .......... ..........
Man.autumnalis   .......... .......... .......... .......... .......... .......... .......... .......... .......... ..........
Man.caulescens   ........-- ---------- ---------- ---------- ---------- .....----- ---------- ---------- ---------- -....-----
Solanum          ........-- ---------- ---------- ---------- ---------- .....----- ---------- ---------- ---------- -....-----
Calceolaria      .......... .T........ .......... .......... .....A.... .......... .......... .......... .......... ..........
Drymonia         .......... .......... .......... .......... .......... .......... .......... .......... .......... ..........
Nematanthus      .......... .......... .......... .......... .......... .......... .......... .......... .......... ..........
Veronica         .......... .......... .......... .......... .......... .......... .......... .......... .....----- -....-----
Digitalis        .......... .......... .......... .......... .......... .......... .......... .......... ....G----- -....-----
Celsia           .......... .......... .......... .......... .......... .......... .......... .......... .......... A.........
Verbascum        ........-- ---------- ---------- ---------- ---------- .....----- ---------- ---------- ---------- -....-----
Justicia         .......... .......... .......... ......GTC. .......... TACAT..... .......... .......... .......... .....TC..G
Barleria         .......... .......... .......... .......... .......... TACAT..... .......... .......... .......... ..........
Thunbergia       .......... .......... .......... .......... .......... GGAAT..... .......... .......... .......... ..........
Sanchezia        .......... .......... .......... ......GTC. .......... TACAT..... .......... ...G...... .......... ..........
Catalpa          .......... .......... .......... .......... .......... .......... .......... .......... .......... ..........
Campsis          ........-- ---------- ---------- ---------- ---------- .....----- ---------- ---------- ---------- -....-----
Ajuga            .......... .......... .......... .......... .......... .......... .......... .......... .......... ..........
Callicarpa       ........-- ---------- ---------- ---------- ---------- .....----- ---------- ---------- ---------- -....-----
Sesamum          .......... .......... .......... .......... .......... .......... .......... .......... .......... ..........
```

	1701	1711	1721	1731	1741	1751	1761	1771	1781	1791
Rhamnus	ATA------T	TA--------	----------	---GTAGAAG	AG-AGAAG--	-----CATCC	AGAGGTGTAT	ATTCCCATTC	TGCCTGGATC	CGGTATCATA
Asimina	C.........	....	....	....	....	.......C..	T..A..T..C	..C.T.....	....	....
Magnolia	---......-	--........	....	...-------	--.-----..	....	....	....	....	....
Knema	....	....	....	....	....	.......C..	T..A..T..C	..C.T.....	....	....
Myristica	....	....	....	....	....	.......C..	T..A..T..C	..C.T.....	....	....
Peperomia	....	....	....	....	....	.......C..	T..A......	....	....	T.........
Piper	---......-	--........	....	...-------	--.-----..	....	....	....	C.........	T.........
Arisaema	....	....	....	....	....	.......C..	T..A..T..C	..C.T.....	.........T	......T..T
Zamioculcas	....	....	....	....	....	.......C..	T..A..T..C	..C.T.....	.........T	......T..T
Xanthosoma	....	....	....	....	....	.......C..	....	.....A....	.........T	......T..T
Philodendron	....	....	....	....	....	.......C..	T..A......	....	.........T	......T..T
Peltandra	---......-	--........	....	...-------	--.-----..	....	....	....	.........T	......T..T
Strelitzia	---......-	--........	....	...-------	--.-----..	....	....	....	....	......T..T
Musa	...TAGT..C	..CCTTTCTT	CTATTCATCC	GCTTATAGTA	GT......AG	AGAAG..C..	T..A..T..C	..C.TA....	....	....
Musella	...TAGT..C	.TCTATTCAT	CCGC......	...T..T.TT	..T.....AG	AGAAG..C..	T..A..T..C	..C.TA....	....	....
Maranta	....	....	....	....	....	.......C..	T..A..T..C	..C.T.....	....	......T..T
Monotagma	....	....	....	....	....	.......C..	T..A..T..C	..C.T.....	....	......T..T
Haumania	---......-	--........	....	...-------	--.-----..	....	....	....	....	......T..T
Globba	....	....	....	....	....	.......C..	T..A..T...	....T.....	.........T	......T..T
Costus	---......-	--........	....	...-------	--.-----..	....	....	....	....	......T..T
Triticum	---......-	--........	....	...-------	--.-----..	....	....	....T.....	.........T	T.....T..T
Grevillea	---......-	--........	....	...-------	--.-----..	....	....	....	....	....
Melia	....	....	....	....	....	.......C..	T..A..T..C	..C.T.....	.A.......T	....
Dysoxylum	....	....	....	....	....	.......C..	T..A..T..C	..C.T.....	....	....
Ailanthus	---......-	--........	....	...-------	--.-----..	....	....	....	....	....
Toxicodendron	---......-	--........	....	...-------	--.-----..	....	....	....	....	....
Rhus	---.......	....	....	....	.A........	.......C..	T..A..T..C	..C.T.....	....	....
Bur.sp	---.......	....	....	....	....	.......C..	T..A..T..C	..C.T.....	....	....
Bur.simarouba	---......-	--........	....	...-------	--.-----..	....	....	....	....	....
Breynia	....	....	....	......A...	....	.......C..	T..A..T..C	....TA....	....	T.........
Phyllanthus	....	....	....	......A...	....	.......C..	T..A..T..C	....TA....	....	T.........
Hevea	C.........	....	....	....	....	.......C..	T..A..T..C	....TA....	....G.....	....
Acalypha	C.........	....	....	....	....	.......C..	T..A..T..C	..C.TA....	....	....
Croton	....	....	....	....	....A.....	.......C..	T..A..T..C	..C.T.....	....G.....	....
Euphorbia	....	....	....	....	.A........	.......C..	T..A..T..C	....TA....	....	....
Hura	....	....	....	....	....	.......C..	T..A..T...	....T.....	....G.....	....
Malpighia	....	....	....	....	....	.......C..	T..A..T..C	..C.TA....	.........T	....
Polygala	GCT..TTTA.	....	....	....	....	.......C..	T..A..T..C	..C.T.....	....	....
Humulus	---......-	--........	....	...-------	--.-----..	....	....	....T.....	.........T	....
Pilea	-.-.......	--........	....	...-------	--.-----..	.......C..	T..A..T...	....T.....	.........T	....
Hovenia	....	....	....	....	....	.......C..	T..A..T...	....T.....	....	....
Cyn.songaricum	---......-	--........	....	...-------	--.-----..	.......C..	T..A..T..C	..C.T.....	.A.......T	....
Cyn.coccineum	....	....	....	....	....	.......C..	T..A..T..C	..C.T.....	.A.......T	....
Citrullus	....	....	....	....	....	.......C..	T..A..T..C	..C.TA....	....	....
Melothria	....	....	....	....	....	.......C..	T..A..T..C	..C.TA....	....	....
Cucurbita	---......-	--........	....	...-------	--.-----..	....	....	....	....	C
Lepionurus	....	....	....	....	....	.......C..	T..A..T..C	..C.T.....	....	....
Andromeda	---......-	--........	....	...-------	--.-----..	....	....	....T.....	.........T	....
Pyrola	....	....	....	....	....	.......C..	T..A..T...	....T.....	.........T	....
Symplocus	....	....	....	....	....	.......C..	T..A..T...	....T.....	....	....
Diospyros	....	....	....	....	....	.......C..	T..A..T..C	..C.T.....	.........T	....
Mitrastema	....TTATA.	....	....	....	....	.......C..	T..A..T..C	..C.T.....	....	....
Barringtonia	....	....	....	....	....	.......C..	....	....	....	....
Daucus	---......-	--........	....	...-------	--.-----..	....	....	....	....	....
Hydrocotyle	....	....	....	....	....	.......C..	T..A..T..C	..C.T.....	....	....
Helianthus	---......-	--........	....	...-------	--.-----..	....	....	....	....	....
Alstonia	....	....	....	....	....	.......C..	T..A..T..C	..C.T.....	....	....
Vinca	....	....	....	....	....	.......C..	T..A..T..C	..C.T.....	....	....
Nerium	....	....	....	....	....	.......C..	T..A..T..C	..C.T.....	....	....
Alyxia	....	....	....	....	....	.......C..	T..A..T..C	..C.T.....	....	....
Ochrosia	....	....	....	....	....	.......C..	T..A..T..C	..C.T.....	....	....
Carissa	---......-	--........	....	...-------	--.-----..	....	....	....	....	....
Frasera	....	....	....	....	....	.......C..	T..A..T..C	..C.TA....	....	....
Coffea	--........	....	....	....	....	....	....	....	....	....
Ixora	--........	....	....	....	....	.......C..	T..A..T..C	..C.T.....	....	....
Heliotropium	....	....	....	....	....	.......C..	T..A..T...	....T.....	....	....
Ehretia	....	....	....	....	....	.......C..	T..A..T..C	..C.T.....	....	....
Borago	---......-	--........	....	...-------	--.-----..	....	....	....	....	....
Ipomoea	.....TATA.	....	....	....	....	....	T..A..T...	....T.....	....G....T	T.........
Schizanthus	---......-	--........	....	...-------	--.-----..	....	....	....	....	....
Goetzea	---......-	--........	....	...-------	--.-----..	....	....	....	....	....
Protoschwenckia	---......-	--........	....	...-------	--.-----..	....	....	....	....A.....	....
Bru.densiflora	---......-	--........	....	...-------	--.-----..	....	....	....	....	....
Bru.jamaicensis	....	....	....	....	....	.......C..	T..A..T...	....T.....	....	....
Bru.grandiflora	---......-	--........	....	...-------	--.-----..	....	....	....	....	....
Nicotiana	---......-	--........	....	...-------	--.-----..	....	....	....	....	....
Atropa	---......-	--........	....	...-------	--.-----..	....	....	....	....	....
Anisodus	---......-	--........	....	...-------	--.-----..	....	....	....	....	....
Atropanthe	---......-	--........	....	...-------	--.-----..	....	....	....	....	....
Phys.orientalis	....	....	....	....	....	.......C..	T..A..T..C	..C.T.....	.A.......T	....
Phys.infundib.	....	....	....	....	....	.......C..	T..A..T..C	..C.T.....	.A.......T	....
Przewalskia	....	....	....	....	....	.......C..	T..A..T..C	..C.T.....	.A.......T	....
Hyos.pusillus	....	....	....	....	....	.......C..	T..A..T..C	..C.T.....	.A.......T	....
Hyos.aureus	....	....	....	....	....	.......C..	T..A..T..C	..C.T.....	.A.......T	....
Lycium	---......-	--........	....	...-------	--.-----..	....	....	....	....	....
Jaborosa	---......-	--........	....	...-------	--.-----..	....	....	....	....	....
Exodeconus	---......-	--........	....	...-------	--.-----..	....	....	....	....	....
Juanulloa	---......-	--........	....	...-------	--.-----..	....	....	....	....	....
Man.officinarum	....	....	....	....	....	.......C..	T..A..T..C	..C.T.....	.A.......T	....
Man.autumnalis	....	....	....	....	....	.......C..	T..A..T..C	..C.T.....	.A.......T	....
Man.caulescens	---......-	--........	....	...-------	--.-----..	....	....	....	....	....
Solanum	---......-	--........	....	...-------	--.-----..	....	....	....	....	....
Calceolaria	....	....	....	....	....	.......C..	T..A..T...	....T.....	....	....
Drymonia	C.........	....	....	....	....G.....	.......C..	T..A..T...	....T.....	....	....
Nematanthus	C.........	....	....	....	....G.....	.......C..	T..A..T...	....T.....	....	....
Veronica	---......-	--........	....	....	....G.....	.......C..	T..A..T...	....T.....	....	....
Digitalis	---......-	--........	....	....	....G.....	.......C..	T..A..T...	....T.....	....	....
Celsia	....	....	....	....	....	.......C..	T..A..T..C	..C.T.....	....	....
Verbascum	---......-	--........	....	...-------	--.-----..	....	....	....	....	....
Justicia	....	....	....	....	....	....	T..A..T..C	..C.TA....	....C....T	....
Barleria	C.........	....	....	....	....	.......C..	T..A..T..C	..C.TA....	....C....T	....
Thunbergia	C.........	....	....	....	....	.......C..	T..A..T..C	..C.TA....	....C....T	....
Sanchezia	C.........	....	....	....	....	.......C..	T..A..T..C	..C.TA....	....C....T	....
Catalpa	....	....	....	....	....	.......C..	T..A..T..C	..C.T.....	....	....
Campsis	---......-	--........	....	...-------	--.-----..	....	....	....	....	....
Ajuga	....	....	....	....	....	.......C..	T..A..T..C	..C.TA....	....	....
Callicarpa	---......-	--........	....	...-------	--.-----..	....	....	....	....	....
Sesamum	....	....	....	....	....	.......C..	T..A..T..C	..C.TA....	....	....

```
                 1801       1811       1821       1831       1841       1851       1861       1871       1881       1891
                 |          |          |          |          |          |          |          |          |          |
Rhamnus          AGTCATATCG TTTCGACTTT TTCGGGAAAA CCGGTCTTCG GGTATCTAGG CATGGTTTAT GCCATGATCA GTATAGGTGT TCTTGGATTT CTTGTTTGGG
Asimina          .......... .A........ .......... .......... .......... .......... .......... ...C...... ..C.....C. ......C...
Magnolia         .......... .A........ .......... .......... .......... .......... .......... ...C...... ..C.....C. ......C...
Knema            .......... .A.....C.. .......... .......... .......... .......... .......... ...C...... ..C.....C. ......C...
Myristica        .......... .A.....C.. .......... .......... .......... .......... .......... ...C...... ..C.....C. ......C...
Peperomia        .......... .A........ .......... .......... .....GAT.. .......... ..A....... .......... .......... ......C...
Piper            .......... .A........ .......... .......... .......... .......... .......... .......... .......... ......C...
Arisaema         .......... .A.....C.. ....A..... .......... .......... .......... .......... .......... ..C....... ..G.......
Zamioculcas      .......... .A.....C.. .......... .......... .......... .......... .......... .......... ..C.....C. ..G.......
Xanthosoma       .......... .A.....C.. ....A..... .......... .......... .......... .......... .......... ..C....... ..G.......
Philodendron     .......... .A.....C.. .......... .......... .......... .......... .......... .......... ..C.....C. ..G.......
Peltandra        .......... .A.....C.. ....A..... .......... .......... .......... .......... .......... ..C.....C. ..G.......
Strelitzia       .......... .A.....C.. ....A..... .......... .......... .......... .......... .......... ..C.....C. ..........
Musa             .......... ....T..... ....AA.... .......... .......... .......... .......... .......... .......... ..........
Musella          .......... ....T..... ....A..... ..T....... .......... .......... .......... .......... .......... ..........
Maranta          .......... .A.....C.. ....A..... .......... .......... .......... .......... .......... ..C.....C. ..........
Monotagma        .......... .A.....C.. ....A..... .......... .......... .......... .......... .......... ..C.....C. ..........
Haumania         .......... .A.....C.. ....A..... .......... .......... .......... .......... .......... ..C.....C. ..........
Globba           .......... .A.....C.. ....A..... .......... .......... .......... .......... ........C. .......... A.........
Costus           .......... .A.....C.. ....A..... .......... .......... .......... .......... .......... ..C.....C. ..........
Triticum         .......... .A.....C.. ...AA..... .......... .......... .......... ........A. .......... .......... ..A.......
Grevillea        .......... .A........ .......... .......... .......... .......... .......... .......... .......... ......C...
Melia            .......... ....C..... .......... ..T....... .......... .......... .......... .......... .......... ..........
Dysoxylum        .......... .......... .......... .......... .......... .......... .......... .......... .......... ..........
Ailanthus        .......... .......... .......... .......... .......... .......... .......... .......... .......... ..........
Toxicodendron      .......... .......... .......... .......... .......... .......... .......... .......... .......... ..........
Rhus             .......... .......... .......... .......... .......... .......... .......... .......... .......... ..........
Bur.sp           .......... .......... .......... .......... .......... .......... .......... .......... .......... ..........
Bur.simarouba    .......... .......... .......... .......... .......... .......... .......... .......... .......... ..........
Breynia          .......... ....T..... .......... .....T.... .......... .......... .......... .......... .......... ..........
Phyllanthus      .......... ....T..... .......... .....T.... .......... .......... .......... .......... .......... ..........
Hevea            .......... .......... .......... .....T.... .......... .......... .......... .......... .......... ..........
Acalypha         .......... .......... .......... .....T.... .......... .......... .......... .......... .......... ..........
Croton           .......... .......... .......... .....T.... .......... .......... .......... .......... .......... ..........
Euphorbia        .......... ....T..... .......... .....T.... .......... .......... .......... .......... .......... ..........
Hura             .......... .......... .......... .....T.... .......... .......... .......... .......... .......... ..........
Malpighia        .......... .......... .......... .....T.... .......... .......... .......... .......... .......... ..........
Polygala         .......... .......... .......... .......... .......... .......... .......... .......... .......... ..........
Humulus          ........T. .......... .......... .......... .......... .......... .......... .......... .......... ..........
Pilea            ........T. .......... .......... .......... .......... .......... .......... .......... .......... ..........
Hovenia          .......... .......... .......... .......... .......... .......... .......... ...C...... .......... ..........
Cyn.songaricum   .......... .......... .......... .......... .......... .......... .......... .......... .......... ..........
Cyn.coccineum    .......... ....C..... .......... ..T....... .......... .......... .......... .......... .......... ..........
Citrullus        .......... .......... .......... .....T.... .......... .......... .......... .......... .......... ..........
Melothria        .......... .......... .......... .....T.... .......... .......... .......... .......... .......... ..........
Cucurbita        .......... ....T..... .......... ..A..T.... .......... .......... .......... .......... .......... ..........
Lepionurus       .......... .......... .......... ..A....... .......... A......... .......... ...C...... ........C. ......C...
Andromeda        .......... .......... .......... .......... .....T.... .......... .......... .......... .......... ..........
Pyrola           .......... .......... .......... .......... .....T.... .......... .......... .......... .......... ..........
Symplocus        ......G... .......... .......... .......... .....T.... .......... .......... .......... .......... ..........
Diospyros        .......... .......... .......... .......... .....T.... .......... .......... .......... .......... ..........
Mitrastema       .......... .......... .......... .......... .....T.... .......... .......... .......... .......... ..........
Barringtonia     .......... .......... .......... ..C....... .....T.... .......... .......... ...C...... ........C. ..........
Daucus           .......... ....T..... .......... .......... .......... .......... .......... .......... .......... ..........
Hydrocotyle      .......... ....T..... .......... .......... .......... .......... .......... .......... ........C. ..........
Helianthus       .......... ....T..... .......... .......... .......... .......... .......... .......... ..C.....C. ..........
Alstonia         .......... .......... .......... .......... .......... .......... .......... .......... .......... ..........
Vinca            .......... .......... ...A...... .......... .......... .......... .......... .......... .......... ..........
Nerium           .......... .......... .......... .......... .......... .......... .......... .......... .......... ..........
Alyxia           .......... .......... .......... .......... .......... .......... .......... .......... .......... ..........
Ochrosia         .......... .......... .......... .......... .......... .......... .......... .......... .......... ..........
Carissa          .......... .......... .......... .......... .......... .......... .......... .......... .......... ..........
Frasera          .......... .......... .......... .......... .......... .......... .......... .......... .......... ..........
Coffea           .......... .......... ...A...... .......... .......... .......... .......... .......... .......... ..........
Ixora            .......... .......... ...A...... .......... .......... .......... .......... .......... .......... ..........
Heliotropium     .......... .......... .......... .......... .......... .......... .......... .......... .......... ..........
Ehretia          .......... .......... .......... .......... .......... .......... .......... .......... .......... ..........
Borago           .......... .......... .......... .......... .......... .......... .......... .......... .......... ..........
Ipomoea          .......... ....T..... .......... .....T.... .......... .......... .......... .......... .......... ..........
Schizanthus      .......... ....T..... .......... .....T.... .......... .......... .......... ........C. .......... ..........
Goetzea          .......... ....T..... .......... .....T.... .......... .......... .......... .......... .......... ..........
Protoschwenckia  .......... ....T..... .......... ..T..T.... .......... .......... .......... .......... .......... ..........
Bru.densiflora   .......... ....T..... .......... .....T.... .......... .......... .......... .......... .......... ..........
Bru.jamaicensis  .......... .......... .......... .......... .......... .......... .......... .......... .......... ..........
Bru.grandiflora  .......... .......... .......... .....T.... .......... .......... .......... .......... .......... ..........
Nicotiana        .......... ....T..... .......... .....T.... .......... .......... .......... .......... .......... ..........
Atropa           .......... ....T..... .......... .....T.... .......... .......... .......... .......... .......... ..........
Anisodus         .......... ....T..... .......... .....T.... .......... .......... .......... .......... .......... ..........
Atropanthe       .......... .......... .......... .....T.... .......... .......... .......... .......... .......... ..........
Phys.orientalis  .......... ....T..... .......... .....T.... .......... .......... .......... .......... .......... ..........
Phys.infundib.   .......... .......... .......... .....T.... .......... .......... .......... .......... .......... ..........
Przewalskia      .......... ....T..... .......... .....T.... .......... .......... .......... .......... .......... ..........
Hyos.pusillus    .......... .......... .......... .....T.... .......... .......... .......... .......... .......... ..........
Hyos.aureus      .......... .......... .......... .....T.... .......... .......... .......... .......... .......... ..........
Lycium           .......... .......... .......... .....T.... .......... .......... .......... .......... .......... ..........
Jaborosa         .......... ....T..... .......... .....T.... .......... .......... .......... .......... .......... ..........
Exodeconus       .......... ....T..... .......... .....T.... .......... .......... .......... .......... .......... ..........
Juanulloa        .......... ....T..... .......... .....T.... .......... .......... .......... .......... .......... ..........
Man.officinarum  .......... ....T..... .......... .....T.... .......... .......... .......... .......... .......... ..........
Man.autumnalis   .......... ....T..... .......... .....T.... .......... .......... .......... .......... .......... ..........
Man.caulescens   .......... ....T..... .......... .....T.... .......... .......... .......... .......... .......... ..........
Solanum          .......... ....T..... .......... .....T.... .......... .......... .......... .......... .......... ..........
Calceolaria      .......... ....T..... .......... .....T.... .......... .......... .......... .......C.. C......... .....G....
Drymonia         .......... .......... .......... .....T.... .......... .......... .......... .......C.. C......... ..........
Nematanthus      .......... .......... .......... .....T.... .......... .......... .......... .......C.. C......... ..........
Veronica         .......... ....T..... .......... ..C..T.... .......... .......... .......... .......C.. C......... ..........
Digitalis        .......... .......... .......... .....T.... .......... .......... .......... .......C.. C......... ..........
Celsia           .......... .......... .......... .....T.... .......... .......... .......... .......C.. C......... ..........
Verbascum        .......... .......... .......... .....T.... .......... .......... .......... .......C.. C......... ..........
Justicia         ........TC ....C..... ......C... ..A..T..T. .......... .......... .......... .......C.. C......... ..........
Barleria         ........T. .......... .......... .....T.... .......... .......... .......... .......C.. C......... ..........
Thunbergia       ........T. .......... .......... .....T.... .......... .......... .......... .......C.. C......... ..........
Sanchezia        ........T. .......... .......... .....T.... .......... .......... .......... .......C.. C......... ..........
Catalpa          .......... .......... .......... .....T.... .......... .......... .......... .......C.. C......... ..........
Campsis          .......... .......... .......... .....T.... .......... .......... .......... .......C.. C......... ..........
Ajuga            .......... ....C..... ...AA..... ..T..T.... .C........ .......... .......... .......C.C C......... ..........
Callicarpa       .......... ....C..... .......... .....T.... .......... .......... .......... .......C.. C......... ..........
Sesamum          .......... .......... .......... .....T.... .......... .......... .......... .......C.. C......... ..........
```

```
                   1901       1911       1921       1931       1941       1951       1961       1971       1981       1991
                   |          |          |          |          |          |          |          |          |          |
Rhamnus            CTCATCATAT GTTTACTGTG GGCTTAGACG TTGATACCCG TGCCTACTTC ACCGCAGCTA CCATGATCAT AGCTGTCCCC ACTGGAATCA AAATCTTTAG
Asimina            .......... .......... .......... .......G.. .......... .......... .......... .......... ..A....... ....A.....
Magnolia           .......... .......... .......... .......G.. .......... .......... .......... .......... ..A....... ..........
Knema              .......... .......... .......... .......G.. .......... .......... .......... .......... .......... ..........
Myristica          .......... .......... .......... .......G.. .......... .......... .......... .......... .......... ..........
Peperomia          .......... ......A... .......... .......G.. .......... .......... .......... ...G...... .......... ....A.....
Piper              .......... ......A... .......... .......G.. .......... .......... .......... ...G...... .......... ..........
Arisaema           .......... .......... ....C..... .......G.. .......... .......... .......... .......... .......... ..........
Zamioculcas        .......... .......... ....C..... .......G.. .......... .......... .......... .......... .......... ..........
Xanthosoma         .......... .......... ....C..... .......G.. .......... .......... .......... .......... ..A.....A. ..........
Philodendron       .......... .......... ....C..... .......G.. .......... .......... .......... .......... ........A. ..........
Peltandra          .......... .......... ....C..... .......G.. .......... .......... .......... .......... .....C..A. ..........
Strelitzia         .......... .......... .......... .......G.. .......... .......... .......... .......... .....G.... ..........
Musa               .......... .......... .......... .......G.. .......... .....T.... .......... .........T .....G.... ..........
Musella            .......... .......... .......... ..-------- ---------- ---------- ---------- ---------- ---------- ----------
Maranta            .......... .......... .......... .......G.. .......... .......... .......... .......... .....G.... ..........
Monotagma          .......... .......... .......... ..-------- ---------- ---------- ---------- ---------- ---------- ----------
Haumania           .......... .......... .......... ..-------- ---------- ---------- ---------- ---------- ---------- ----------
Globba             .......... .......... .......... .......G.. .......... .......... .......... .......... .....G.... ..........
Costus             .......... .......... .......... .......G.. .......... .....T.... .......... .......... .....G.... ..........
Triticum           .......... .......... .......... .......G.. .......... .......... .......... ......G... ..A....... ..........
Grevillea          .......... .......... ....C..... .......G.. .......... .......... .......... ...A...... .....G.... ..........
Melia              .......... .......... .......... .......... .......... .......... .......... .......... .......... ..........
Dysoxylum          .......... .......... .......... .......... .......... .......... .......... .......... .......... ..........
Ailanthus          .......... .......... .......... .......... .......... .......... .......... .......... .......... ..........
Toxicodendron        .......... .......... .......... .......... .......... .......... .......... .......... .......... ..........
Rhus               .......... .......... .......... .......... .......... .......... .......... .......... .......... ..........
Bur.sp             .......... .......... .......... .......... .......... .......... .......... .......... .......... ..........
Bur.simarouba      .......... .......... .......... .......... .......... .......... .......... .......... .......... ..........
Breynia            .......... .......... .......... .......... .........T .......... .......... .......... ........T. ..........
Phyllanthus        .......... .......... .......... .......... .........T .......... .......... .......... ........T. ..........
Hevea              .......... .......... .......... .......... .......... .......... .......... .......... .......... ..........
Acalypha           .......... .......... .......... .......... .......... .......... .......... ...A...... .......... ..........
Croton             .......... .......... .......... .......... .......... .......... .......... .......... ........A. ..........
Euphorbia          .......... .......... .......... .......... .......... .......... .......... .......... .......... ..........
Hura               .......... .......... .......... .......... .......... .......... .......... .......... ........A. ..........
Malpighia          .......... .......... .......... .......... .......... .....G.... .......... .......... .......... ..........
Polygala           .......... .......... .......... .......... .......... .......... .......... ......A... ..A....... ..........
Humulus            .......... .......... ........T. .......... .......... .......... .......... .......... ........A. ..........
Pilea              .......... .......... ........T. .......... .......... .......... .......... .......... .......... ..........
Hovenia            .......... .......... .......... .......... .......... .......... .......... .......... .......... ..........
Cyn.songaricum     .......... .......... .......... .......... .......... .......... .......... .......... .......... ..........
Cyn.coccineum      .......... .......... .......... .......... .......... .......... .......... .......... .......... ....A.....
Citrullus          .......... .......... .......... .......... .......... .....T.... .......... ...A...... .......... ..........
Melothria          .......... .......... .......... .......... .......... .....T.... .......... ...A...... .......... ..........
Cucurbita          .......... .......... .......... .......... .......... .....T.... .......... ...A...... .......... ..........
Lepionurus         .......... .......... .......... .......... .......... .......... .......... .......... .......... ..........
Andromeda          .......... .......... .......... .......... .......... .......... .......... .......... .......... ..........
Pyrola             .......... .........A .......... .......... .......... .......... .......... .......... .......... ..........
Symplocus          .......... .......... .......... .......... .......... .......... .......... .......... .......... ..........
Diospyros          .......... .......... .......... .......... .......... .......... .......... .......... .......... ..........
Mitrastema         .......... .......... .......... .......... .......... .......... .......... .......... .......... ..........
Barringtonia       .......... .......... .......... .......... .........T .......... .......... .......... .......... ..........
Daucus             .......... .......... .......... .......... .......... .......... .......... .......... ........A. ....A.....
Hydrocotyle        .......... .......... ..A....... .......... .......... .......... .......... .......... ........A. ..........
Helianthus         .......... .......... .......... .......... .......... .......... .......... .......... .......... ..........
Alstonia           .......... .......... .......... .......... G......... .......... .......... .......... ........A. ..........
Vinca              .......... .......... .......... .......... G......... .......... .......... .......... .......... ..........
Nerium             .......... .......... .......... .......... G......... .......... .......... .......... ........A. ..........
Alyxia             .......... .......... .......... .......... G......... .......... .......... .......... .......... ..........
Ochrosia           .......... .......... .......... .......... G......... .......... .......... .......... ........A. ..........
Carissa            .......... .......... .......... .......... G......... .......... .......... .......... ........A. ..........
Frasera            .......... .......... .......... .......... .......... .......... .......... .......... .......... ..........
Coffea             .......... .......... .......... .......... .......... .......... .......... .......... .......... ..........
Ixora              .......... .......... .......... .......... .......... .......... .......... .......... .......... ..........
Heliotropium       .......... .......... .......... .......... .......... .......... .......... .......... .......... ..........
Ehretia            .......... .......... .......... .......... .......... .......... .......... .......... .......... ..........
Borago             .......... .......... .......... .......... .......... .......... .......... .......... .......... ..........
Ipomoea            .......... .......... .......... .......... .......... .......... .......... .......... ..A.....A. ..........
Schizanthus        .......... .......... .......... .......... .......... .......... .......... .......... .......... ..........
Goetzea            .......... .......... .......... .......... .......... .......... .......... .......... .......... ..........
Protoschwenckia    .......... .......... .......... .......... .......... .......... .......... .......... .......... ..........
Bru.densiflora     .......... .......... .......... .......... .......... .......... .......... .......... .......... ..........
Bru.jamaicensis    .......... .......... .......... .......... .......... .......... .......... .......... .......... ..........
Bru.grandiflora    .......... .......... .......... .......... .......... .......... .......... .......... .......... ..........
Nicotiana          .......... .......... .......... .......... .......... .......... .......... .......... .......... ..........
Atropa             .......... .......... .......... .......... .......... .......... .......... .......... .......... ..........
Anisodus           .......... .......... .......... .......... .......... .......... .......... .......... .......... ..........
Atropanthe         .......... .......... .......... .......... .......... .......... .......... .......... .......... ..........
Phys.orientalis    .......... .......... .......... .......... .......... .......... .......... .......... .......... ..........
Phys.infundib.     .......... .......... .......... .......... .......... .......... .......... .......... .......... ..........
Przewalskia        .......... .......... .......... .......... .......... .......... .......... .......... .......... ..........
Hyos.pusillus      .......... .......... .......... .......... .......... .......... .......... .......... .......... ..........
Hyos.aureus        .......... .......... .......... .......... .......... .......... .......... .......... .......... ..........
Lycium             .......... .......... .......... .......... .......... .......... .......... .......... .......... ..........
Jaborosa           .......... .......... .......... .......... .......... .......... .......... .......... .......... ..........
Exodeconus         .......... .......... .......... .......... .......... .......... .......... .......... .......... ..........
Juanulloa          .......... .......... .......... .......... .......... .......... .......... .......... .......... ..........
Man.officinarum    .......... .......... .......... .......... .......... .......... .......... .......... .......... ..........
Man.autumnalis     .......... .......... .......... .......... .......... .......... .......... .......... .......... ..........
Man.caulescens     .......... .......... .......... .......... .......... .......... .......... .......... ........A. ..........
Solanum            .......... .......... .......... .......... .......... .......... .......... .......... .......... ..........
Calceolaria        .......... .......... .......... .......... .......... .......... .......... .......... .......... ..........
Drymonia           .......... .......... .......... .......... ...A...... .......... .......... .......... .......... ..........
Nematanthus        .......... .......... .......... .......... ...A...... .......... .......... .......... .......... ..........
Veronica           .......... .......... .......... .......... .......... .......... .......... .......... .......... ..........
Digitalis          .......... .......... .......... .......... .......... .......... .......... .......... .......... ..........
Celsia             .......... .......... .......... .......... .......... .......... .......... .......... .......... ..........
Verbascum          .......... .......... .......... .......... .......... .......... .......... .......... .......... ..........
Justicia           .......... .......... .......... .......... .......... .......... .......... .......... .......... ..........
Barleria           .......... .......... .......... .......... .......... .......... .......... .......... .......... ..........
Thunbergia         .......... .......... .......... .......... .......... .......... .......... .......... .......... ..........
Sanchezia          .......... .......... .......... .......... .......... .......... .......... .......... .......... ..........
Catalpa            .......... .......... .......... .......... .......... .......... .......... .......... .......... ..........
Campsis            .......... .......... .......... .......... .......... .......... .......... .......... .......... ..........
Ajuga              .......... .......... .......... .......... .......... .......... .......T.. .......... .......... ..........
Callicarpa         .......... .......... .......... .......... .......... .......... .......T.. .......... .......... ..........
Sesamum            .......... .......... .......... .......... .......... .......... .......... .......... .......... ..........
```

```
                  2001       2011       2021       2031       2041       2051       2061       2071       2081       2091
                  |          |          |          |          |          |          |          |          |          |
Rhamnus           TTGGATCGCT ACCATGTGGG GGGGTTCGAT ACAATACAAA ACACCCATGT TATTTGCTGT AGGGTTCATC TTTTTGTTTA CCATAGGAGG ACTCACTGGA
Asimina           .......... .......... .A........ .......... .......... .......... .....C.... ........C. .......... ..........
Magnolia          .......... .......... .A........ .......... .......... .......... .....C.... ........C. .......... ..........
Knema             .......... .......... .A........ .......... .......... .......... .....C.... ........C. .......... ..........
Myristica         .......... .......... .A........ .......... .......... .......... .....C.... ........C. .......... ..........
Peperomia         .......... .......... .A........ .......... .....A.... .......... .....C.... ........C. .......... ..........
Piper             .......... .......... .A........ .......... .......... .......... .....C.... ........C. .......... ..........
Arisaema          .......... .......... .A........ .......... .......... .......... .......... ........C. .A........ G.........
Zamioculcas       .......... .......... .A........ .......... .......... .......... .....C.... ........C. .A........ G.........
Xanthosoma        .......... .......... .A........ .......... .......... .......... .....C.... ........C. .A........ G.........
Philodendron      .......... .......... .A........ .......... .......... .......... .....C.... ........C. .A........ G.........
Peltandra         .......... .......... .A........ .......... .......... .......... .....C.... ........C. .A........ G.........
Strelitzia        .......... .......... .A........ .....T.... .......... .......... .....C.... ........C. .......... G.........
Musa              .......... ..T....... .A........ .....T.... .......... .......A.. .....C.... ........C. .T........ G.........
Musella           ---------- ---------- ---------- ---------- ---------- ---------- ---------- ---------- ---------- ----------
Maranta           .......... .......... .A........ .....T.... .......... .......... .....C.... ........C. .......... G.........
Monotagma         ---------- ---------- ---------- ---------- ---------- ---------- ---------- ---------- ---------- ----------
Haumania          ---------- ---------- ---------- ---------- ---------- ---------- ---------- ---------- ---------- ----------
Globba            .......... .......... .A........ .....T.... .......... .......... .......... ........C. .......... G.........
Costus            .......... .......... .A........ .....T.... .......... .......... .....C.... ........C. .......... G.........
Triticum          .......... .......... .A........ .......... .......... .......... .......... ........C. .......... G.........
Grevillea         .......... .......... .A........ .......... .......... .......... .......... ........C. .......... ..........
Melia             .......... .......... .......... .......... .......... .......... .......... ........C. .T........ ..........
Dysoxylum         .......... .......... .......... .......... .......... .......... .......... ........C. .T........ ..........
Ailanthus         .......... .......... .......... .......... .......... .......... .......... ........C. .T........ ..........
Toxicodendron       .......... .......... .......... .......... .......... .......... .......... ........C. .T........ ..........
Rhus              .......... .......... .......... .......... .......... .......... .......... ........C. .T........ ..........
Bur.sp            .......... .......... .......... .......... .......... .......... .......... ........C. .T........ ..........
Bur.simarouba     .......... .......... .......... .......... .......... .......... .......... ........C. .T........ ..........
Breynia           ......T... .......... .......... .......... .......... .......... .......... ........C. .......... ..........
Phyllanthus       ......T... .......... .......... .......... .......... .......... .......... ........C. .......... ..........
Hevea             .......... .......... .......... .......... .......... .......... .......... ........C. .......... ..........
Acalypha          .......... .......... .......... .......... .......... .......... .......... ........C. .......... ..........
Croton            .......... .......... .......... .......... .......... .......... .......... ........C. .......... ..........
Euphorbia         .......... .......... .......... .......... .......... .......... .......... ........C. .......... ..........
Hura              .......... .......... .......... .......... .......... .......... .......... ........C. .......... ..........
Malpighia         .......... .......... .T........ .......... .......... .......... .......... ........C. .......... ..........
Polygala          .........G .......... .......... .......... .......... .......A.. ......TC.A ........C. .......... ..........
Humulus           .......... .......... .......... .......... .......... .......... .......... ........C. .......... ..........
Pilea             .......... .......... .......... .......... .......... .......... .......... ........C. .......... ..........
Hovenia           .......... .......... .......... .......... .......... .......... .......... ........C. .......... ..........
Cyn.songaricum    .......... .......... .......... .......... .......... .......... .......... ........C. .T........ ..........
Cyn.coccineum     .......... .......... .......... .......... .......... .......... ...C...... ........C. .T........ ..........
Citrullus         .......... .......... .......... .......... .......... .......... .....C.... ........C. .......... ..........
Melothria         .......... .......... .......... .......... .......... .......... .....C.... ........C. .......... ..........
Cucurbita         .......... .......... .......... .......... ..C....... .......... .......... ........C. .......... ..........
Lepionurus        .......... .......... .......... .......... .......... .......... .......... ........C. .......... ..........
Andromeda         .......... .......... .......... .......... .....T.... .......... .......... ........C. .......... ......A...
Pyrola            .......... .......... .......... .......... .....T.... .......... .......... ........C. .......... ......A...
Symplocus         .......... .......... .......... .......... .......... .......... .....C.... ........C. .G........ ..........
Diospyros         .......... .......... .......... .......... .......... .......... .......... ........C. .......... ..........
Mitrastema        .......... .......... .......... .......... .......... .......... .....C.... ..C.....C. .......... ......A...
Barringtonia      .......... .......... .......... .......... .......... .......... .....C.... ........C. .......... ..........
Daucus            .......... .......... .......... .......... .......... .......... .....C.... ........C. .......... ..........
Hydrocotyle       .......... .......... .......... .......... .......... .......... .....C.... ........C. .......... ..........
Helianthus        .......... .......... .......... .......... .......... .......... .....C.... ........C. .......G.. G.........
Alstonia          .......... .......... .......... .......... .......... .......... .......... .......... .......... ..........
Vinca             .......... .......... .......C.. .......... .......... .......... .......... .......... .......G.. ..........
Nerium            .......... .......... .......... .......... .......... .......... .....C.... .......... .......... ..........
Alyxia            .......... .......... .......... .......... .......... .......... .....C.... .......... .......... ..........
Ochrosia          .......... .......... .......... .......... .......... .......... .......... .......... .......... ..........
Carissa           .......... .......... .......... .......... .......... .......... .....C.... .......... .......... ..........
Frasera           .......... .......... .......... .......... .......... .......... .....C.... .......... .......... ..........
Coffea            .......... .......... .......A.. .......... .......... .......... .......... .......... .......... ..........
Ixora             .......... .......... .......A.. .......... .......... .......... .......... .......... .......... ..........
Heliotropium      .......... .......... .......... .......... .......... .......... ...A...... ........C. .......... ..........
Ehretia           .......... .......... .......... .......... .......... .......... .......... ........C. .......... ..........
Borago            .......... .......... .......... .......... .......... .......... .......... ........C. .......... ..........
Ipomoea           .......... .......... .......... .......... .......... .......... .......... ........C. .......... ..........
Schizanthus       .......... .......... .......... .......... .......... .......... .......... ........C. .......... ..........
Goetzea           .......... .......... .......... .......... .......... .......... .......... ........C. .......... ..........
Protoschwenckia   .......... .......... .......... .......... .......... .......... .......... ........C. .......... ..........
Bru.densiflora    .......... .......... .......... .......... .......... .......... .......... ........C. .......... ..........
Bru.jamaicensis   .......... .......... .......... .......... .......... .......... .......... ........C. .......... ..........
Bru.grandiflora   .......... .......... .......... .......... .......... .......... .......... ........C. .......... ..........
Nicotiana         .......... .......... .......... .......... .......... .......... .......... ........C. .......... ..........
Atropa            .......... .......... .......... .......... .......... .......... .......... ........C. .......... ..........
Anisodus          .......... .......... .......... .......... .......... .......... .......... ........C. .......... ..........
Atropanthe        .......... .......... .......... .......... .......... .......... .......... ........C. .......... ..........
Phys.orientalis   .......... .......... .......... .......... .......... .......... .......... ........C. .......... ..........
Phys.infundib.    .......... .......... .......... .......... .......... .......... .......... ........C. .......... ..........
Przewalskia       .......... .......... .......... .......... .......... .......... .......... ........C. .......... ..........
Hyos.pusillus     .......... .......... .......... .......... .......... .......... .......... ........C. .......... ..........
Hyos.aureus       .......... .......... .......... .......... .......... .......... .......... ........C. .......... ..........
Lycium            .......... .......... .......... .......... ..C....... .......... .......... ........C. .......... ..........
Jaborosa          .......... .......... .......... .......... .......... .......... .......... ........C. .......... ..........
Exodeconus        .......... .......... .......... .......... .......... .......... .......... ........C. .......... ..........
Juanulloa         .......... .......... .......... .......... .......... .......... .......... ........C. .......... ..........
Man.officinarum   .......... .......... .......... .......... .......... .......... .......... ........C. .......... ..........
Man.autumnalis    .......... .......... .......... .......... .......... .......... .......... ........C. .......... ..........
Man.caulescens    .......... .......... .......... .......... .......... .......... .......... ........C. .......... ..........
Solanum           .......... .......... .......... .......... .......... .......... .......... .....A..C. .......... ..........
Calceolaria       .......... .......... .......A.. .......... .......... .......... .......... ........C. .......... ..........
Drymonia          .......... .......... .......... .......... .......... .......... .......... ........C. .......... ..........
Nematanthus       .......... .......... .......... .......... .......... .......... .......... ........C. .......... ..........
Veronica          .......... .......... .......... .......... .......... .......... .......... ........C. .......... ......G...
Digitalis         .......... .......... .......... .......... .......... .......... .......... ........C. .......... ......G...
Celsia            .......... .......... .......... .......... .......... .......... .......... ........C. .......... ......G...
Verbascum         .......... .......... .......... .......... .......... .......... ...-...... ........C. .......... ......G...
Justicia          .......... .......... .A........ .G........ .........C .......... .......... ........C. .......... ...G..G...
Barleria          .......... .......... .......... .......... .......... .......... .......... ........C. .......... ......G...
Thunbergia        .......... .......... .......... .......... .......... .......... .......... ........C. .......... ......G...
Sanchezia         .......... .......... .......... .......... .......... .......... .......... ........C. .......... ......G...
Catalpa           .......... .......... .......... .......... .......... .......... .......... ........C. .......... ......G...
Campsis           .......... .......... .......... .......... .......... .......... .......... ........C. .......... ......G...
Ajuga             .......... .......... .C........ .......... .......... ........T. .......... ........C. .......... ......A...
Callicarpa        .......... .......... .......... .......... .......... .......... ...-...... ........C. .......... ......G...
Sesamum           .......... .......... .......... .......... .......... .......... .......... ........C. .......... ......G...
```

```
                2101       2111       2121       2131       2141       2151       2161       2171       2181       2191
                |          |          |          |          |          |          |          |          |          |
Rhamnus         ATAGTCCCGG CAAATTCTGG GCTAGACATT GCTCTACATG ATACTTATTA TGTGGTTGCA CATTTCCATT ATGTACTTTC TATGGGAGCA GTTTTTGCTT
Asimina         .......... .......... .......... .......... .......... ..C....... .......... .......... .........C ...C......
Magnolia        .......... .......... .......... .......... .......... ..C....... .......... .......... .........C ...C......
Knema           .......... .......... .......... .......... .......... ..C....... .......... .......... .........C ..........
Myristica       .......... .......... .......... .......... .......... ..C....... .......... .......... .........C ..........
Peperomia       .......... .G........ .......... .......... .......... .--------- ---------- ---------- ---------- ----------
Piper           ......T... .......... .......... .......... .......... ..C....... .......... .......... .........C ..........
Arisaema        .....T.TA. .T........ .......... .......... .......... ..C....... .......... .......... .........C ..........
Zamioculcas     .....T.TA. .T........ .......... .......... .......... ..C....... .......... .......... .........C ..........
Xanthosoma      .....T.TA. .......... .......... .......... .......... ..C....... .......... .......... .........C ..........
Philodendron    .....T.TA. .T.....A.. .......... .......... .......... ..C....... .......... .......... .........C ..........
Peltandra       .....T.TA. .T........ .......... .......... .......... ..C....... .......... .......... .........C ..........
Strelitzia      .....T.TA. .......... .......... .......... .......... .......... .......... .......... .........C ..........
Musa            .....T.TA. .......C.. .......... .......... .......... ...A...... .......... .......C.. .........C ..........
Musella         ---------- ---------- ---------- ---------- ---------- ---------- ---------- ---------- ---------- ----------
Maranta         .....T.TC. .......... .......... .......... .......... .......... .......... .......... .........C ..........
Monotagma       ---------- ---------- ---------- ---------- ---------- ---------- ---------- ---------- ---------- ----------
Haumania        ---------- ---------- ---------- ---------- ---------- ---------- ---------- ---------- ---------- ----------
Globba          .....T.TA. .T........ .......... .......... .......... .......... .......... .......... .........C ..........
Costus          .....T.TA. .......... .......... .......... .......... .......... .......... .......... .........C ..........
Triticum        .....T.TA. ....C..... .......... .......... .......... .......... .......... .......... .........C ..........
Grevillea       ......T... .T........ .......... .......... .......... ..C....... .......... .......... .........C ..........
Melia           .......... .......... .......... .......... .......... .......... .......... .......... .........C ..........
Dysoxylum       .......... .T........ .......... .......... .......... .......... .......... .......... .........C ..........
Ailanthus       .......... .......... .......... .......... .......... .......... .......... .......... .........C ..........
Toxicodendron     ..-------- ---------- ---------- ---------- ---------- ---------- ---------- ---------- ---------- ----------
Rhus            .......... .T........ .......... .......... .......... .......... .......... .......... .........C ..........
Bur.sp          .....A.... .......... .......... .......... .......... .......... .......... .......... .........C ..........
Bur.simarouba   ......T... .T........ .......... .......... .......... .......... .......... .......... .........C ..........
Breynia         .......... .......A.. .......... .......... .......... .......... .......... .......... .........C ..........
Phyllanthus     .......... .......A.. .......... .......... .......... .......... .......... .......... .........C ..........
Hevea           .......... .......... .......... .......... .......... .......... .......... .......... .........C ..........
Acalypha        .......... .......... .......... .......... .......... .......... .......... .......... .........C ..........
Croton          .......... .......... .......... .......... .......... .......... .......... .......... .........C ..........
Euphorbia       .......... .......... .......... .......... .......... .......... .......... .......... .........C ..........
Hura            .......... .......... .......... .......... .......... .......... .......... .......... .........C ..........
Malpighia       .......... .......... .......... .......... .......... .......... .......... .......... .........C ..........
Polygala        .......... .......... .......... .......... .......... .......... .......... .......... .........C ..........
Humulus         ......T... .......... .......... .......... .......... .......... .......... .......... .........C ..........
Pilea           ......T... .......... .......... .......... .......... .......... .......... .......... .........C ..........
Hovenia         .......... .......A.. .......... .......... .......... .......... .......... .......... .........C ..........
Cyn.songaricum  ......T... .T........ .......... .......... .......... .......... .......... .......... .........C ..........
Cyn.coccineum   ......T... .T........ .......... .......... .......... .......... .......... .......... .........C ..........
Citrullus       .......... .......A.. .......... .......... .......... .......... .......... .......... .........C ..........
Melothria       .......... .......A.. .......... .......... .......... .......... .......... .......... .........C ..........
Cucurbita       .......... .......A.. .......... .......... .......... .........G .......... .......... .........C ..........
Lepionurus      .......... .......... .......... .......... .......... ..C....... .......... .......... .........C ..........
Andromeda       .....----- ---------- ---------- ---------- ---------- ---------- ---------- ---------- ---------- ----------
Pyrola          ......T... .......... .......... .......... .......... .......... .......... .......... .........C ..........
Symplocus       ......T... .......... .......... .......... .......... .......... .......... .......... .........C ..........
Diospyros       ......T... .T........ .......... .......... .......... .......... .......... .......... .........C ..........
Mitrastema      ......T... .......... .......... .......... .......... .......... .......... .......... .........C ..........
Barringtonia    .......... .......... ......T... .......... .......... .......... .......... ....G..... .........C ..........
Daucus          .....T.... .......... .......... .......... .......... .......... .......... .......... .........C ..........
Hydrocotyle     .....T.... .......... .......... .......... .......... .......... .......... .......... .........C ..........
Helianthus      .....T.... .......... .......... .......... .......... .......... .......... .......... .........C ..........
Alstonia        .....T.T.. .......... .......... .......... .......... .......... .......... .......... .........C ..........
Vinca           .....T.T.. .......... .......... .......... .......... .......... .......... .......... .........C ..........
Nerium          .....T.T.. .......... .......... .......... .......... .......... .......... .......... .........C ..........
Alyxia          .....T.T.. .......... .......... .......... .......... .......... .......... .......... .........C ..........
Ochrosia        .....T.T.. .......... .......... .......... .......... .......... .......... .......... .........C ..........
Carissa         .....T.T.. .......... .......... .......... .......... .......... .......... .......... .........C ..........
Frasera         .....T.T.. .......... .......... .......... .......... .......... .......... .......... .........C ..........
Coffea          .....T.T.. .......... A......... .......... .......... ...A...... .......... .......... .........C ..........
Ixora           .....T.T.. .......... A......... .......... .......... ...A...... .......... .......... .........C ..........
Heliotropium    .....T.T.. .......... .......... .......... .......... .......... .......... .......... .........C ..........
Ehretia         .....T.T.. .......... .......... .......... .......... .......... .......... .......... .........C ..........
Borago          .....T.T.. .......... .......... .......... .......... .......... .......... .......... .........C ..........
Ipomoea         .....T.T.. .......... .......... .......... .......... .......... .......... .......... .........C ..........
Schizanthus     ..T..T.T.. .T........ .......... .......... .......... .......... .......... .......... .........C ..........
Goetzea         .....T.T.. .......... .......... .......... .......... .......... .......... .......... .........C ..........
Protoschwenckia .....T.T.. .T........ .......... .......... .......... .......... .......... .......... .........C ..........
Bru.densiflora  ...A.T.T.. .T........ .......... .......... .......... .......... .......... .......... .........C ..........
Bru.jamaicensis ...A.T.T.. .T........ .......... .......... .......... .......... .......... .......... .........C ..........
Bru.grandiflora .....T.T.. .......... .......... .......... .......... .......... .......... .......... .........C ..........
Nicotiana       .....T.T.. .T........ .......... .......... .......... .......... .......... .......... .........C ..........
Atropa          .....T.T.. .T........ .......... .......... .......... .......... .......... .......... .........C ..........
Anisodus        .....T.T.. .T........ .......... .......... .......... .......... .......... .......... .........C ..........
Atropanthe      .....T.T.. .G........ .......... .......... .......... .......... .......... .......... .........C ..........
Phys.orientalis .....T.T.. .T........ .......... .......... .......... .......... .......... .......... .........C ..........
Phys.infundib.  .....T.T.. .T........ .--------- ---------- ---------- ---------- ---------- ---------- ---------- ----------
Przewalskia     .....T.T.. .T........ .......... .......... .......... .......... .......... .......... .........C ..........
Hyos.pusillus   .....T.T.. .T........ .......... .......... .......... .......... .......... .......... .........C ..........
Hyos.aureus     .....T.T.. .T........ .......... .......... .......... .......... .......... .......... .........C ..........
Lycium          ..T..T.T.. .......A.. .......... .......... .......... .......... .......... .......... .........C ..........
Jaborosa        .....T.T.. .T........ .......... .......... .......... .......... .......... .......... .........C ..........
Exodeconus      .....T.T.. .T........ .......... .......... .......... .......... .......... .......... .........C ..........
Juanulloa       .....T.T.. .......... .......... .......... .......... .......... .......... .......... .........C ..........
Man.officinarum .....T.T.. .T........ .......... .......... .......... .......... .......... .......... .........C ..........
Man.autumnalis  .....T.T.. .T........ .......... .......... .......... .......... .......... .......... .........C ..........
Man.caulescens  .....T.T.. .T........ .......... .......... .......... .......... .......... .......... .........C ..........
Solanum         .....T.T.. .T........ .......... .......... .......... .......... .......... .......... .........C ..........
Calceolaria     .....T.T.. .......... .......... .......... .......... .......... .......... .......... .........C ..........
Drymonia        .....T.T.. .......... .......... .......... .......... .......... .......... .......... .........C ..........
Nematanthus     .....T.T.. .......... .......... .......... .......... .......... .......... .......... .........C ..........
Veronica        .....T.T.. .......... .......... .......... .......... .......... ..C....... .......... .........C ..........
Digitalis       ..C..T.T.. .T........ .......... .......... .......... .......... .......... .......... .........C ..........
Celsia          .....T.T.. .......... .......... .......... .......... .......... .......... .......... .........C ..........
Verbascum       .....T.--- ---------- ---------- ---------- ---------- ---------- ---------- ---------- ---------- ----------
Justicia        .....T.T.. .......... .......... ........C. .......... .......... .......... .......... .........C ..........
Barleria        .....T.T.. .......... .......... .......... .......... .......... .......... .......... .........C ..........
Thunbergia      .....T.T.. .......... .......... .......... .......... .......... .......... .......... .........C ..........
Sanchezia       .....T.T.. .......... .......... .......... .......... .......... .......... .......... .........C ..........
Catalpa         .....T.T.. .......... .......... .......... .......... .......... .......... .......... .........C ..........
Campsis         .....T.T.. .......... .......... .......... .......... .......... .......... .......... .........C ..........
Ajuga           .....T.T.. .....G.G.. .......... .......... .......... .......... .......... .......... .........C ..........
Callicarpa      ...-.T.T.- .-...----- ---------- ---------- ---------- ---------- ---------- ---------- ---------- ----------
Sesamum         .....T.T.. .......... .......... .......... .......... .......... .......... .......... .........C ..........
```

```
                  2201       2211       2221       2231       2241       2251       2261       2271       2281       2291
                  |          |          |          |          |          |          |          |          |          |
Rhamnus           TATTTGCAGG ATTTTACTAT TGGGTTGGTA AAATCTTTGG TCGGACATAC CCTGAAACTT TAGGTCAAAT CCATTTTTGG ATCACTTCTT TCGGGGTTAA
Asimina           .......... ....C..... .....G.... .......... .......... .........C .......... .......... .......... ..........
Magnolia          .......... ....C..... .....G.... .......... .......... .........C .......... .......... .......... ..........
Knema             .......... ....C..... .....G.... .......... .......... .......... .......... .......... .......... ..........
Myristica         .......... ....C..... .......... .......... .......... .......... .......... .......... .......... ..........
Peperomia         ---------- ---------- ---------- ---------- ---------- ---------- ---------- ---------- ---------- ----------
Piper             .......... ....C..... .....G.... .......... .......... .......... .......... .......... .......... ....A.....
Arisaema          .......... ....C..... .....G.... .......... .......... .......... .......... .......... .......T.. ..........
Zamioculcas       .......... ....C..... .....G.... .......... .........T .......... .......... .......... .......T.. ..........
Xanthosoma        .......... ....C..... .....G.... .......... .......... .......... .......... .......... .......T.. ..........
Philodendron      .......... ....C..... .....G.... .......... .......... .......... .......... .......... .......T.. ..........
Peltandra         .......... ....C..... .....G.... .......... .......... .......... .......... .......... .......T.. ..........
Strelitzia        .......... ....C..... .....G.... .......... .......... .......... ....C..... .......... .......... ..........
Musa              .......... ....C..... .....G.... .......... .......... .......... ....A..... N.....C... .......... ..........
Musella           ---------- ---------- ---------- ---------- ---------- ---------- ---------- ---------- ---------- ----------
Maranta           .......... .......... .....G...C .......... .......... .......... ....C..... .......... .......... ..........
Monotagma         ---------- ---------- ---------- ---------- ---------- ---------- ---------- ---------- ---------- ----------
Haumania          ---------- ---------- ---------- ---------- ---------- ---------- ---------- ---------- ---------- ----------
Globba            .......... .......... .....G...C .......... .......... .......... ....C..... .......... .......T.A ..........
Costus            .......... ....C..... .....G.... .......... .......... .......... ....C..... .......... .......... ..........
Triticum          .......T.. .......... .....G.... .......... .........T .......... ....C..... .......... .......T.. ..........
Grevillea         .......... ....C..... .....G.... .......... .......... .......... .......... .......... .......... ..........
Melia             .......... ....C..... .....G.... .......... .......... .......... .......... .......... .......T.. .........G
Dysoxylum         .......... ....C..... .....G.... .......... .......... .......... .......... .......... .......T.. ..........
Ailanthus         .......... ....C..... .....G.... .......... .......... .......... .......... .......... .......T.. ..........
Toxicodendron       ---------- ---------- ---------- ---------- ---------- ---------- ---------- ---------- ---------- ----------
Rhus              .......... ....C..... .....G.... .......... .......... .......... .......... .......... .......T.. ..........
Bur.sp            .......... ....C..... .....G.... .......... .......... .......... .......... .......... ..A....T.. ..........
Bur.simarouba     .......... ....C..... .....G.... .......... .......... .......... .......... .......... .....C.T.. ..........
Breynia           .......C.. .......... .....G.... .......... .......... .......... .......... .......... .......T.. ....A..G..
Phyllanthus       .......C.. .......... .....G.... .......... .......... .......... .......... .......... .......T.. ....A..G..
Hevea             .......... ....C..... .....G.... .......... .......... .......... .......... .......... .......T.. ..........
Acalypha          .......... .......... .....G..A. .......... .......... .......... .......... .......... .......T.. ..........
Croton            .......... ....C.T... .....G.... .......... .......... .......... .......... .......... .......T.. ..........
Euphorbia         .......... ....C..... .....G.... .......... .......... .......... .......... .......... .......T.. ..........
Hura              .......... ....C..... .....G.... .......... .......... .......... .......... .......... .......T.. ..........
Malpighia         .......... .........C .....G.... .......... .......... .......... .......... .......... .......T.. ..........
Polygala          .......... .......... .....G.... .......... .......... .......... .......... .......... .......T.. ..........
Humulus           .......... .......... .......... .......... .......... .......... .......... .......... .......T.. ..........
Pilea             .......... .......... .......... .......... .......... .......... .......... .......... .......T.. ..........
Hovenia           .......... .......... .......... .......... .......... .......... .......... .......... .......... ..........
Cyn.songaricum    .......... .......... .....G.... .......... .......... .......... .......... .......... .......T.. ..........
Cyn.coccineum     .......... .......... .....G.... .......... .......... .......... .......... .......... .......T.. .......G..
Citrullus         .......... ....C..... .....G.... .......... .......... .......... .......... .......... .......T.. ..........
Melothria         .......... ....C..... .....G.... .......... .......... .......... .......... .......... .......T.. ..........
Cucurbita         .......... ....C..... .....G.... .......... .......... .....G.... ....G..... .......... .......T.. ..........
Lepionurus        .......... ....C..... .....G.... .......... .......... .......... .......... .......... .......T.. ..........
Andromeda         ---------- ---------- ---------- ---------- ---------- ---------- ---------- ---------- ---------- ----------
Pyrola            .......... G...C..... .....A.... .......... .........T .......... .......... .......... .......T.. ..........
Symplocus         .......... ....C..... .....A.... .......... .......... .......... .......... .......... .......T.. ..........
Diospyros         .......... ....C..... .....A.... .......... .......... .......... .......... .......... .......... ..........
Mitrastema        .......... ....C..... .....A.... .......... .........T .......... .......... .......... .......T.. ..........
Barringtonia      .......... ....C..... .....A.... .......... .......... .......... .......... .......... .......T.. ....A.....
Daucus            .......... ....C..... .....A.... .......... .........T .......... .......... .......... .......T.. .......G..
Hydrocotyle       .......... ....C..... .....A.... .......... .........T .......... .......... .......... .......T.. .......G..
Helianthus        .......... ....C..... .....A.... .......... ...A.....T .......... .......... .......... .......T.. ..........
Alstonia          .......... ....C..... .....A.... .......... .......... .......... .......... .......... ..A....T.. ..........
Vinca             .......... ....C..... .....A.... .......... .......... .......... .......... .......... ..A....T.. ..........
Nerium            .......... ....C..... .....A.... ....A..... .......... .......... .......... .......... ..A....T.. ..........
Alyxia            .......... ....C..... .....A.... .......... .......... .......... .......... .......... ..A....T.. ..........
Ochrosia          .......... ....C..... .....A.... .......... .......... .......... .......... .......... ..A....T.. ..........
Carissa           .......... ....C..... .....A.... .......... .......... .......... .......... .......... ..A....T.. ..........
Frasera           .......... ....C..... .....A.... .......... .......... .......... .....A.... .......... .......T.. ..........
Coffea            .......... ....C..... .....A.... .......... .......... .......... .......... .......... ..A....T.. .T........
Ixora             .......... ....C..... .....A.... .......... .......... .......... .......... .......... ..A....T.. .T........
Heliotropium      .......... ....C..... .....A.... ..G....... .......... .......... .......... .......... .......T.. ..........
Ehretia           .......... ....C..... .....A.... .......... .......... .......... .......... .......... .......T.. ..........
Borago            .......... ....C..... .....A.... .......... .......... .......... .......... .......... .......T.. .......G..
Ipomoea           .......... ....C..... .....A.... .......... .......... .......... .......... .......... .......T.. .......G..
Schizanthus       .......... ....C..... .....A.... .......... .......... .......... .......... .......... .......T.. ..........
Goetzea           .......... ....C..... .....A.... .......... .......... .....G.... .......... .......... .......T.. .......G..
Protoschwenckia   .......... ....C..... .....A.... .......... .......... .......... .......... .......... .......T.. ..........
Bru.densiflora    .......... ....C..... .....A.... .......... .......... .......... .......... .......... .......T.. .......G..
Bru.jamaicensis   .......... ....C..... .....A.... .......... .......... .......... .......... .......... .......T.. .......G..
Bru.grandiflora   .......... ....C..... .....A.... .......... .......... .......... .......... .......... .......T.. .......G..
Nicotiana         .......... ....C..... .....A.... .......... .......... .......... .......... .......... .......T.. ..........
Atropa            .......... ....C..... .....A.... .......... .......... .......... .......... .......... .......T.. ..........
Anisodus          .......... ....C..... .....A.... .......... .......... .......... .......... .......... .......T.. ..........
Atropanthe        .......... ....C..... .....A.... .......... .......... .......... .......... .......... .......T.. ..........
Phys.orientalis   .......... ....C..... .....A.... .......... .......... .......... .......... .......... .......T.. ..........
Phys.infundib.    ---------- ---------- ---------- ---------- ---------- ---------- ---------- ---------- ---------- ----------
Przewalskia       .......... ....C..... .....A.... .......... .......... .......... .......... .......... .......T.. ..........
Hyos.pusillus     .......... ....C..... .....A.... .......... .......... .......... .......... .......... .......T.. ..........
Hyos.aureus       .......... ....C..... .....A.... .......... .......... .......... .......... .......... .......T.. ..........
Lycium            .......... ....C..... .....A.... .......... .......... ..C....... .......... .......... .......T.. ..........
Jaborosa          .......... ....C..... .....A.... .......... .......... .......... .......... .......... .....C.T.. ..........
Exodeconus        .......... ....C..... .....A.... .......... .......... .......... .......... .......... .......T.. ..........
Juanulloa         .......... ....C..... .....A.... .......... .......... .......... .......... .......... .......T.. ..........
Man.officinarum   .......... ....C..... .....A.... .......... .......... .......... .......... .......... .......T.. ..........
Man.autumnalis    .......... ....C..... .....A.... .......... .......... .......... .......... .......... .......T.. ..........
Man.caulescens    .......... ....C..... .....A.... .......... .......... .......... .......... .......... .......T.. ..........
Solanum           .......... ....C..... .....A.... .......... .......... .......... .......... .......... .....C.T.. ..........
Calceolaria       .......... ....C..... .....A.... .......... .......... .......... .....A.... .......... .......T.. ..........
Drymonia          .......... ....C..... .....A.... .......... .......... .......... .......... .......... .......T.. ..........
Nematanthus       .......... ....C..... .....A.... .......... .......... .......... .......... ........T. .......T.. ..........
Veronica          .......... ....C..... .....A.... .......... .........T .......... .......... .......... .......T.A ..........
Digitalis         .......... ....C..... .....A.... .......... .......... .......... .......... .......... .......T.C .......G..
Celsia            .......... .......... .....A.... .......... .......... .......... .......... .......... .......T.. ..........
Verbascum         ---------- ---------- ---------- ---------- ---------- ---------- ---------- ---------- ---------- ----------
Justicia          .......... .......... .....A.... .......... .......... .......... .......... .......... .......T.. ..........
Barleria          .......... ....C..... .....A.... .......... .......... .......... .......... .......... .......T.. ..........
Thunbergia        .......... ....C..... .....A.... .......... .......... .......... .......... .......... .......T.. ..........
Sanchezia         .......... ....C..... .....A.... .......... .......... .......... .......... .......... .......T.. ..........
Catalpa           .......... ....C..... .....A.... .......... .......... .......... .......... .......... .......T.. ..........
Campsis           .......... ....C..... .....A.... .......... .......... .......... .......... .......... .......T.. ..........
Ajuga             .......... ....C..... ....CA.... .......... G......... ..G....... ....G..... .......... .....C.T.G .......G..
Callicarpa        ---------- ---------- ---------- ---------- ---------- ---------- ---------- ---------- ---------- ----------
Sesamum           .......... ....C..... .....A.... .......... .......... .......... .......... .......... .......T.A ..........
```

```
                  2301       2311       2321       2331       2341       2351       2361       2371       2381       2391
                  |          |          |          |          |          |          |          |          |          |
Rhamnus           TCTGACCCTC TTTCCCATGC ATTTCTTAGG GCTTTCGGGT ATGCCACGTC GCATTCCAGA TTATCCAGAT GCTTACGCTG GATGGAACGC CCTTAGCAGT
Asimina           ...T...... .......... .......... .......... .......... .......... .......... .......... .......T.. ..........
Magnolia          ..C....... .......... .......... .......... .......... .......... .......... .......... .......T.. ..........
Knema             ..C....... .......... .......... .......... .......... .......... .......... .......... .......T.. ..........
Myristica         ..C....... .......... .......... .......... .......... .......... .......... .......... .......T.. ..........
Peperomia         ---------- ---------- ---------- ---------- ---------- ---------- ---------- ---------- ---------- ----------
Piper             ..C....... .......... .......... .......... .......... .......... ..T....... ........A. .......T.. ..........
Arisaema          .......... .......... .......... .......... .......... .......... .......... ........C. .......T.. T..G......
Zamioculcas       .......... C......... .......... .......... .......... .......... .......... ........C. .......T.. T..G......
Xanthosoma        .......... .......... .......... .......... .......... .......... .......... ........C. .......T.. T..G......
Philodendron      .......... .......... .......... .......... .......... .......... .......... ........C. .......T.. T..G......
Peltandra         .......... .......... .......... .......... .......... .......... .......... ........C. .......T.. T..G......
Strelitzia        ..C....... .......... .......... .......... .......... .......... .......... ........C. .......T.. T..G......
Musa              .......T.. .......... .......... ...A...... .....T.... .......C.. .C....N... ..C..T..C. .......T.T T---------
Musella           ---------- ---------- ---------- ---------- ---------- ---------- ---------- ---------- ---------- ----------
Maranta           ..C....... .......... .......... .......... .......... .......... .......... ........C. .......T.. T..G......
Monotagma         ---------- ---------- ---------- ---------- ---------- ---------- ---------- ---------- ---------- ----------
Haumania          ---------- ---------- ---------- ---------- ---------- ---------- ---------- ---------- ---------- ----------
Globba            .......T.. .......... .......... .......... .......... .......... .......... ........C. .......T.. T..G......
Costus            ..C....... .......... .......... .......... .......... .......... .......... ........C. .......T.. T..G......
Triticum          .......T.. .......... .......... .......... .......... .......... .......... ........C. .......T.. T..G......
Grevillea         ..C....... .......... .......... .......... .......... .......... .......... .......... .......T.. ..........
Melia             .......T.. .......... .......... .......... .......... .......... .......... .......... .......T.. ..........
Dysoxylum         .......T.. .......... .......... .......... .......... .......... .......... .......... .......T.. ..........
Ailanthus         .......T.. .......... .......... .......... .......... .......... .......... .......... .......T.. ..........
Toxicodendron       ---------- ---------- ---------- ---------- ---------- ---------- ---------- ---------- ---------- ----------
Rhus              .......T.. .......... .......... .......... .......... .......... .......... .......... .......T.. ..........
Bur.sp            .......T.. .......... .......... .......... .......... .......... .......... .......... .......T.. ..........
Bur.simarouba     .......T.. .......... .......... .......... .......... .......... .......... .......... .......T.. T.........
Breynia           ...T...T.. .......... .......... .......... .......... .......... .......... .......... .......T.. ..........
Phyllanthus       ...T...T.. .......... .......... .......... .......... .......... .......... .......... .......T.. ..........
Hevea             ..C....T.. .......... .......... .......... .......... .......... .......... .......... .......T.. ..........
Acalypha          ..C....T.. .......... .......... .......... .......... .......... .......... .......... .......T.. ..........
Croton            .......T.. .......... .......... .......... .......... .......... .......... .......... .......T.. ..........
Euphorbia         ..C....T.. .......... .......... .......... .......... .......... .......... .......... .......T.. ..........
Hura              ..C....T.. .......... .......... .......... .......... .......... .......... .......... .......T.. ..........
Malpighia         .......T.. .......... .......... .......... .......... .......... .......... .....T.... .......T.. ..........
Polygala          .......T.. .......... .......... .......... .......... .......... .......... .......... .......T.. ..........
Humulus           .......T.. .......... .......... .......... .......... .......... .......... .......... .......... ..........
Pilea             .......T.. .......... .......... .......... .......... .......G.. .......... .......... .......... ..........
Hovenia           .......... .......... .......... .......... .......... .......... .......... .......... .......... ..........
Cyn.songaricum    ......TT.. .......... .......... .......... .......... .......... ..T....... .......... .......T.. ..........
Cyn.coccineum     ......TT.. .......... .......... .......... .......... .......... ..T....... .......... .......T.. ..........
Citrullus         ..C....... .......... .......... .......... .......... .......T.. .......... .......... .......T.. ..........
Melothria         ..C....... .......... .......... .......... .......... .......T.. .......... .......... .......T.. ..........
Cucurbita         ..C....... .......... .....C.... .......... ........G. .......... .......... .......... .......T.. ..........
Lepionurus        ..C....... .......... .......... .......... .......... .......... .......... ........A. .......T.. ..........
Andromeda         ---------- ---------- ---------- ---------- ---------- ---------- ---------- ---------- ---------- ----------
Pyrola            ..CT...T.. .....T.... .......... .......... .......... .......... .......... .......... .......T.. ..........
Symplocus         ..CT...T.. .....T.... .......... .......... .......... .......... .......... .......... .......T.. ..........
Diospyros         ..C....T.. .....T.... .......... .......... .......... .......... .......... .......... .......T.. ..........
Mitrastema        ..C....T.T .....T.... .......... .......... .......... .......... .......... ...C.T.... .......T.. ...A......
Barringtonia      ..CT...T.. .....T.... .......... .......... .......... .......... .......... .....T.... .......T.. ..........
Daucus            ..C....T.T .......... .......... .......... .......... .......... .......... .......... .......T.. ..........
Hydrocotyle       .......T.. .......... .......G.. .......... .......... .......... .......... .......... .......T.. ..........
Helianthus        ..C....T.. .......... .......... .......... .......... .......... .......... .......... .......T.. ..........
Alstonia          .......T.. .......... .......... .......... .......... .......... .......... .......... .......T.. ..........
Vinca             .......T.. .......... .......... .......... .......... .......... .......... .......... .......T.. ..........
Nerium            .......T.. .......... .......... .......... .......... .......... .......... .......... .G.....T.. ..........
Alyxia            .......T.. .......... .......... .......... .......... .......... .......... .......... .......T.. ..........
Ochrosia          .......T.. .......... .......... .......... .......... .......... .......... .......... .......T.. ..........
Carissa           .......T.. .......... .......... .......... .......... .......... .......... .......... .......T.. ..........
Frasera           .......T.. .......... .......... .......... .......... .......... .......... .......... .......T.. ..........
Coffea            .......T.. .......... .......... .......... .......... .......... .......... .......... .......T.. ..........
Ixora             .......T.. .......... .......... .......... .......... .......... .......... .......... .......T.. ..........
Heliotropium      .......T.. .......... .......... .......... .......... .......... .......... .......... .......T.. ..........
Ehretia           .......T.. .......... .......... .......... .......... .......... .......... .......... .......T.. ..........
Borago            ...T...T.. .......... .......... .......... .......... .......... .......... .......... .......T.. ..........
Ipomoea           ...T...T.. .....T.... .......... .......... .......... .......... .......... .......... .......T.. ..........
Schizanthus       .A.....T.. .....T.... .......... .......... .......... .......... .......... .......... .......T.. ..........
Goetzea           .A.....T.. .....T.... .......... .......... .......... .......... .......... .......... .......T.. ..........
Protoschwenckia   .A.....T.. .....T.... .......... .......... .......... .......... .......... .......... .......T.. ..........
Bru.densiflora    .A.....T.. .....T.... .......... .......... .......... .......... .......... .......... .......T.. ..........
Bru.jamaicensis   .A.....T.. .....T.... .......... .......... .......... .......... .......... .......... .......T.. ..........
Bru.grandiflora   .A.....T.. .....T.... .......... .......... .......... .......... .......... .......... .......T.. ..........
Nicotiana         .A.....T.. .....T.... .......... .......... .......... .......... .......... .......... .......T.. ..........
Atropa            .A.....T.. .....T.... .......... .......... .......... .......... .......... .......... .......T.. ..........
Anisodus          .A.....T.. .....T.... .......... .......... .......... .......... .......... .......... .......T.. ..........
Atropanthe        .A.....T.. .....T.... .......... .......... .......... .......... .......... .......... .......T.. ..........
Phys.orientalis   .A.....T.. .....T.... .......... .......... .......... .......... .......... .......... .......T.. ..........
Phys.infundib.    ---------- ---------- ---------- ---------- ---------- ---------- ---------- ---------- ---------- ----------
Przewalskia       .A.....T.. .....T.... .......... .......... .......... .......... .......... .......... .......T.. ..........
Hyos.pusillus     .A.....T.. .....T.... .......... .......... .......... .......... .......... .......... .......T.. ..........
Hyos.aureus       .A.....T.. .....T.... .......... .......... .......... .......... .......... .......... .......T.. ..........
Lycium            .A.....T.. .....T.... .......... .......... .......... .......... .......... .......... .......T.. ..........
Jaborosa          .A.....T.. .....T.... .......... .......... .......... .......... .......... .......... .......T.. ..........
Exodeconus        .A.....T.. .....T.... .......... .......... .......... .......... .......... .......... .......T.. ..........
Juanulloa         .A.....T.. .....T.... .......... .......... .......... .......... .......... .......... .......T.. ..........
Man.officinarum   .A.....T.. .....T.... .......... .......... .......... .......... .......... .......... .......T.. ..........
Man.autumnalis    .A.....T.. .....T.... .......... .......... .......... .......... .......... .......... .......T.. ..........
Man.caulescens    .A.....T.. .....T.... .......... .......... .......... .......... .......... .......... .......T.. ..........
Solanum           .A.....T.. .....T.... .......... .......... .......... .......... .......... .......... .......T.. ..........
Calceolaria       .......T.. .....T.... .......... .......... .......... .......... .......... .......... .......T.. ..........
Drymonia          .......T.. .....T.... .......... .......... .......... .......... .......... .......... .......T.. ..........
Nematanthus       .......T.. .....T.... .......... .......... .......... .......... .......... .......... .......T.. ..........
Veronica          .......T.. .....T.... .......... .......... .......... .......... .......... .......... .......T.. ..........
Digitalis         .......T.. .....T.... .......... .......... .......... .......... .......... .......... .......T.. ..........
Celsia            .......T.. .....T.... .......... .......... .......... .......... .......... ........G. .......T.. ..........
Verbascum         ---------- ---------- ---------- ---------- ---------- ---------- ---------- ---------- ---------- ----------
Justicia          .......T.. .....T.... .......... .......... .......... .......... .......... .......... .......T.. ..........
Barleria          .......T.. .....T.... .......... .......... .......... .......... .......... .......... .......T.. ..........
Thunbergia        .......T.. .....T.... .......... .......... .......... .......... .......... .......... .......T.. ..........
Sanchezia         .......T.. .....T.... .......... .......... .......... .......... .......... .......... .......T.. ..........
Catalpa           .......T.. .....T.... .......... .......... .......... .......... .......... .......... .......T.. ..........
Campsis           .......T.. .....T.... .......... .......... .......... .......... .......... .......... .......T.. ..........
Ajuga             .......T.. .....T.... .......... .......... .......... .......... .......... .......... .......T.. .A........
Callicarpa        ---------- ---------- ---------- ---------- ---------- ---------- ---------- ---------- ---------- ----------
Sesamum           .......T.. .....T.... .......... .......... .......... .......... .......... .......... .......T.. ..........
```

```
                 2401       2411       2421       2431       2441       2451       2461       2471
                 |          |          |          |          |          |          |          |
Rhamnus          TTTGGCTCTT ATATATCCGT AGTTGGGATT CGTCGTTTCT TCGTGGTCGT AACAATCACT TCAAGCAGTG GAAATAAC
Asimina          .C....C... .......... .......... .......... .......... .......... ........G. ....C...
Magnolia         .C....C... .......... .......... .......... .......... .......... .......... ....C...
Knema            .C....C... .......... .......... .......... .......... .......... .......... ....C...
Myristica        .C....C... .......... .......... .......... .......... .......... .......... ....C...
Peperomia        ---------- ---------- ---------- ---------- ---------- ---------- ---------- --------
Piper            .C........ .......... .......... .......... .......... C......... .......... ....C...
Arisaema         ..C....... .......... .......... .......... .......... .......... .......... ....G...
Zamioculcas      ..C....... .......... .......... .......... .......... .......... .......... ....G...
Xanthosoma       ..C....... .......... .......... .......... .......... .......... .......... ....G...
Philodendron     ..C....... .......... .......... .......... .......... .......... .......... ....G...
Peltandra        ..C....... .......... .......... .......... .......... .......... .......... ....G...
Strelitzia       ..C....... .......... .......... .......... .......... .......... .......... ....G...
Musa             ---------- ---------- ---------- ---------- ---------- ---------- ---------- --------
Musella          ---------- ---------- ---------- ---------- ---------- ---------- ---------- --------
Maranta          ..C....... .......... .......... .......... .......... C......... .......... ....G...
Monotagma        ---------- ---------- ---------- ---------- ---------- ---------- ---------- --------
Haumania         ---------- ---------- ---------- ---------- ---------- ---------- ---------- --------
Globba           ..G....... .......... .......... T..TC..... .......... .......... .T........ ....G...
Costus           ..C....... .......... .......... .......... .......... C......... .......... ....G...
Triticum         ..C..T.... .......... .......... .......... ....A..T.. CG........ .......... ....G...
Grevillea        ......C... .......... .......... .......... .......... .G........ .......... ....C...
Melia            .......... .......... .......... .......... .......... .......... .......... ........
Dysoxylum        .......... .......... .......... .......... .......... .......... .......... ........
Ailanthus        .......... .......... .......... .......... .......... .......... .......... ........
Toxicodendron      ---------- ---------- ---------- ---------- ---------- ---------- ---------- --------
Rhus             .......... .......... .......... .......... .......... .......... .......... ........
Bur.sp           .......... .......... .......... .......... .......... .......... .......... ........
Bur.simarouba    .......... .......... .......... .......... .......... .......... .......... ........
Breynia          ........A. .......... .......... .......... .T........ .......... .......... ........
Phyllanthus      ........A. .......... .......... .......... .T........ .......... .......... ........
Hevea            .......... .......... .......... .......... .......... .......... .......... ........
Acalypha         .......... .......... ......A... .......... .......... .......... .......... ........
Croton           .......... .......... .......... .......... .......... .......... .......... ........
Euphorbia        .......... .......... .......... .......... .......... .......... .......... ........
Hura             .......... .......... .......... .......... .......... .......... .......... ........
Malpighia        .......... .......... .......... .......... .......... .G........ .......... ........
Polygala         .......... .......... .......... T......... .......... .......... .......... ........
Humulus          .......... .......... .......... T..T...... .......... .......... .T........ ........
Pilea            G.G..A.... .......... .......... T..T...... .........G .......... .GTT...... ........
Hovenia          .......... .......... .......... .......... .......... .......... .......... ........
Cyn.songaricum   .......... ........A. .......... T..T...... .......... .......G.. .T...T.... ....G...
Cyn.coccineum    .......... ........A. .......... T..T...... .......... .......G.. .T...T.... ........
Citrullus        .......... .......... .......... .......... .......... .......... .......... ........
Melothria        .......... .......... .......... .......... .......... .......... .......... ........
Cucurbita        .......... .......... .......... .......... .......... C......... .......... ........
Lepionurus       .C........ .......... .......... .......... .......... .......... .......... ....C...
Andromeda        ---------- ---------- ---------- ---------- ---------- ---------- ---------- --------
Pyrola           .......... .......... .......... T......... .......... .......... .....T.... ....C...
Symplocus        .......... .......... .......... T......... .......... .......... .T........ ....C...
Diospyros        .......... .......... .......... T......... .......... .......... .......... ....C...
Mitrastema       .......... .......... .......... T.......T. .......... .....C.... .......... ....C..T
Barringtonia     .......... .......... .......... T....G.... .......... .......... .......... .G..G...
Daucus           .C........ .......... .......... T......... .......... .......... .......... ....C...
Hydrocotyle      .C........ .......T.. .......... T......... .......... .......... .......A.. ....C...
Helianthus       .C........ .......T.. .......... T......... .......... .......... .......... ....C...
Alstonia         .......... .......... .......... T......... .......... .......... .......... ....G...
Vinca            .......... .......... .......... T......... .......... .......... .......... ....G...
Nerium           .......... .......... .......... T......... .......... .......... .......... ....G...
Alyxia           .......... .......... .......... T......... .......... .......... .......... ....G...
Ochrosia         .......... .......... .......... T......... .......... .......... .......... ....G...
Carissa          ......C... .......... .......... T......... .......... .......... .......... ....G...
Frasera          .......... .C........ .......... T.......T. .......... .......... .......... ....G...
Coffea           .......... .......... .......... T..T...... .......... .......... .......... ....G...
Ixora            .......... .......... .......... T..T...... .......... .......... .......... ....G...
Heliotropium     .......... .......... .......... T..T...... .......... .......... .......... ....G...
Ehretia          .......... .......... .......... T..T...... .......... .......... .......... ....G...
Borago           .......... .......... .......... T..T...... .......... .......... .......... ....G...
Ipomoea          .......... .......... .......... T......... .......... .......... .......... ....G...
Schizanthus      .......... .......... .......... TC........ .......... .......... .......... ....G..G
Goetzea          .......... .......... .......... T......... .......... .......... .......... ....G...
Protoschwenckia  .......... .......... .......... T......... .......... .......... .......... ....G...
Bru.densiflora   .......... .......... .......... T......... .......... .......... .......... ....G...
Bru.jamaicensis  .......... .......... .......... T......... .......... .......... .......... ....G...
Bru.grandiflora  .......... .......... .......... T......... .......... .......... .......... ....G...
Nicotiana        .......... .......... .......... T......... .......... ......A... .......... ....G...
Atropa           .......... .......... .......... T......... .......... .......... .......... ....G...
Anisodus         .......... .......... .......... T......... .......... .......... .......... ....G...
Atropanthe       .......... .......... .......... T......... .......... .......... .......... ....G...
Phys.orientalis  .......... .......... .......... T......... .......... .......... .......... ....G...
Phys.infundib.   ---------- ---------- ---------- ---------- ---------- ---------- ---------- --------
Przewalskia      .......... .......... .......... T......... .......... .......... .......... ....G...
Hyos.pusillus    .......... .......... .......... T......... .......... .......... .......... ....G...
Hyos.aureus      .......... .......... .......... T......... .......... .......... .......... ....G...
Lycium           .......... .......... .......... T......... .......... .......... .......... ....G...
Jaborosa         .......... .......... .......... T......... .......... .......... .......... ....G...
Exodeconus       .......... .......... .......... T......... .......... .......... .......... ....G...
Juanulloa        .......... .......... .......... T......... .......... .......... .......... ....G...
Man.officinarum  .......... .......... .......... T......... .......... .......... .......... ....G...
Man.autumnalis   .......... .......... .......... T......... ---------- ---------- ---------- --------
Man.caulescens   .......... .......... .......... T......... .......... .......... .......... ....G...
Solanum          .......... .......... .......... T......... .......... .......... .......... ....G...
Calceolaria      .......... .......... .......... T......... .......... .......... .......... ....G...
Drymonia         .......... .......... .......... T......... .......... .......... .......... ....G...
Nematanthus      .......... .......... .......... T......... .......... .......... .......... ....G...
Veronica         .......... .......... .......... T......... .......... .......... .......... ....G...
Digitalis        .......... .......... .......... T......... .......... .......... .......... ....G...
Celsia           .......... .......... .......... T..T...... .......... .......... .......... ....G...
Verbascum        ---------- ---------- ---------- ---------- ---------- ---------- ---------- --------
Justicia         .......... .......T.. .......... GC..C..... .......... .......... .......... ....A.--
Barleria         .......... .......... .......... T......... .......... .......... .......... ....G...
Thunbergia       .......... .......... .......... T......... .......... .......... .......... ....G...
Sanchezia        ........AA C......... .......... T......... .......... .......... .......... ....G...
Catalpa          .......... .......... .......... T......... .......... .......... .......... ....G...
Campsis          .......... .......... .......... T......... .......... .......... .......... ....G...
Ajuga            .....A.... .......... ........A. T......... .......... .......... .......... ....G...
Callicarpa       ---------- ---------- ---------- ---------- ---------- ---------- ---------- --------
Sesamum          .......... .......... .......... T......... .......... .......... .......... ....G...
```
