# Supplementary material for: Multiple recent horizontal transfers of the cox1 intron in Solanaceae and extended co-conversion of flanking exons
Source: BMC Evol Biol. 2011 Sep 27;11:277. doi: 10.1186/1471-2148-11-277 (PMC3192709; doi:10.1186/1471-2148-11-277)
Supplement: Additional file 3 — Taxonomic information and GenBank accession numbers. Taxonomic information and GenBank accession numbers of all taxa included in the analyses shown in Figures 3 and 4. [file 1471-2148-11-277-S3.PDF]

Additional File 3. Taxonomic information and GenBank accession numbers of all taxa included in the analyses shown in Figures 3 and 4.

Intron?	Order	Family	Species Name	GenBank Accession Number [a]
Superasterids				
	Apiales	Apiaceae	*Daucus carota*	AY820131
*	Apiales	Araliaceae	*Hydrocotyle rotundifolia*	AJ223424
	Asterales	Asteraceae	*Helianthus annuus*	EU281044
*	Ericales	Ebenaceae	*Diospyros virginiana*	AJ223417
	Ericales	Ericaceae	*Andromeda glaucophylla*	EU156501
*	Ericales	Ericaceae	*Pyrola secunda*	AJ247582
*	Ericales	Lecythidaceae	*Barringtonia asiatica*	AJ247581
*	Ericales	Mitrastemonaceae	*Mitrastema yamamotoi*	EU281021
*	Ericales	Symplocaceae	*Symplocos paniculata*	AJ223435
*	Gentianales	Apocynaceae	*Alstonia plumosa* (JM3105)	EU069541
*	Gentianales	Apocynaceae	*Alyxia loeseneriana* (IND-PK429)	EU069542
	Gentianales	Apocynaceae	*Carissa sp.*	EU156496
*	Gentianales	Apocynaceae	*Nerium oleander*	AJ223431
*	Gentianales	Apocynaceae	*Ochrosia elliptica* (IND-JM3241)	EU069544
*	Gentianales	Apocynaceae	*Vinca rosea*	AJ223423
	Gentianales	Gentianaceae	*Frasera caroliniensis*	EU281038
*	Gentianales	Rubiaceae	*Coffea arabica*	AJ247586
*	Gentianales	Rubiaceae	*Ixora sp. Qiu95051*	AJ247587
*	Lamiales	Acanthaceae	*Barleria prionitis*	AJ247601
*	Lamiales	Acanthaceae	*Justicia americana*	AJ247602
*	Lamiales	Acanthaceae	*Sanchezia nobilis*	AJ223437
*	Lamiales	Acanthaceae	*Thunbergia erecta*	AJ247603
	Lamiales	Bignoniaceae	*Campsis grandiflora*	EU156495
*	Lamiales	Bignoniaceae	*Catalpa fargesii*	AJ223411
*	Lamiales	Calceolariaceae	*Calceolaria sp.*	AJ247585
*	Lamiales	Gesneriaceae	*Drymonia serrulata*	AJ247579
*	Lamiales	Gesneriaceae	*Nematanthus hirsutus*	AJ247578
*	Lamiales	Lamiaceae	*Ajuga reptans*	AJ247595
	Lamiales	Lamiaceae	*Callicarpa sp.*	EU156520
*	Lamiales	Pedaliaceae	*Sesamum indicum*	AJ247598
*	Lamiales	Plantaginaceae	*Digitalis purpurea*	AJ223415
*	Lamiales	Plantaginaceae	*Veronica catenata*	AJ223427
*	Lamiales	Scrophulariaceae	*Celsia arturus*	AJ247590
	Lamiales	Scrophulariaceae	*Verbascum*	EU156513
*	Santalales	Opiliaceae	*Lepionurus sylvestris*	AJ223439
*	Solanales	Convolvulaceae	*Ipomoea sp.*	EU156517
	Solanales	Solanaceae	*Nicotiana tabacum*	BA000042
	Solanales	Solanaceae	*Solanum lycopersicum*	X54738
*	Unplaced	Boraginaceae	*Ehretia anacua*	AJ247606
*	Unplaced	Boraginaceae	*Heliotropium arborescens*	AJ223425
Rosids				
*	Cucurbitales	Cucurbitaceae	*Citrullus lanatus cv. Dixielee*	EU069546
	Cucurbitales	Cucurbitaceae	*Cucurbita pepo*	GU321912
*	Cucurbitales	Cucurbitaceae	*Melothria indica* (IND-PK333)	EU069549
*	Fabales	Polygalaceae	*Polygala sanguinea*	EU281061
*	Malpighiales	Euphorbiaceae	*Acalypha sp.* Qiu95079	AJ247597
*	Malpighiales	Euphorbiaceae	*Croton sp.* Qiu 94027	AJ247608
*	Malpighiales	Euphorbiaceae	*Euphorbia milii*	AJ223418
*	Malpighiales	Euphorbiaceae	*Hevea brasiliensis*	AJ223436
*	Malpighiales	Euphorbiaceae	*Hura crepitans*	AJ247584
*	Malpighiales	Malpighiaceae	*Malpighia glabra*	AJ223433

*	Malpighiales	Phyllanthaceae	*Breynia nivosa*	AJ247605
*	Malpighiales	Phyllanthaceae	*Phyllanthus gneissicus* (IND-JM3235)	EU069552
	Rosales	Cannabaceae	*Humulus lupulus*	EU281047
*	Rosales	Rhamnaceae	*Hovenia dulcis*	AJ247583
*	Rosales	Rhamnaceae	*Rhamnus cathartica*	AJ223422
*	Rosales	Urticaceae	*Pilea fontana*	AJ247580
*	Rosales	unplaced	*Cynomorium coccineum*	EU281023
	**Rosales**	**unplaced**	***Cynomorium songaricum* (5941, 5947)[b]**	**JF966299, JF966300**
*	Sapindales	Anacardiaceae	*Rhus glabra*	EU281065
	Sapindales	Anacardiaceae	*Toxicodendron radicans*	EU156505
*	Sapindales	Burseraceae	*Bursera sp. Qiu 94206*	AJ223412
	Sapindales	Burseraceae	*Bursera simarouba*	EU281030
*	Sapindales	Meliaceae	*Dysoxylum canalense* (IND-10003)	EU069558
*	Sapindales	Meliaceae	*Melia toosendan*	AJ223420
	Sapindales	Simaroubaceae	*Ailanthus altissima*	EU281024
Stem Eudicots				
	Proteales	Proteaceae	*Grevillea robusta*	AY009449
Magnoliids				
*	Magnoliales	Annonaceae	*Asimina triloba*	AY009433
	Magnoliales	Magnoliaceae	*Magnolia stellata*	U77622
*	Magnoliales	Myristicaceae	*Knema latericia*	AJ223430
*	Magnoliales	Myristicaceae	*Myristica fragrans*	AJ223434
*	Piperales	Piperaceae	*Peperomia cubensis*	AF029783
	Piperales	Piperaceae	*Piper bicolor*	AY009448
Monocots				
*	Alismatales	Araceae	*Arisaema triphyllum*	AY009454
	Alismatales	Araceae	*Peltandra virginica*	AJ007550
*	Alismatales	Araceae	*Philodendron oxycardium*	AJ223438
*	Alismatales	Araceae	*Xanthosoma mafaffa*	AJ223807
*	Alismatales	Araceae	*Zamioculcas zamiifolia*	AJ007547
	Poales	Poaceae	*Triticum aestivum*	AP008982
	Zingiberales	Costaceae	*Costus pulverulentus*	AY673038
	Zingiberales	Marantaceae	*Haumania sp. Harris 6672*	AY673021
*	Zingiberales	Marantaceae	*Maranta leuconeura*	AJ223432
*	Zingiberales	Marantaceae	*Monotagma laxum*	AY673026
*	Zingiberales	Musaceae	*Musa acuminata*	AJ247609
*	Zingiberales	Musaceae	*Musella lasiocarpa*	AY673040
	Zingiberales	Strelitziaceae	*Strelitzia reginae*	EU069571
*	Zingiberales	Zingiberaceae	*Globba sessiliflora*	EU069565

a- GenBank accession numbers of new sequences are shown in bold.

b- 5941, Jianhua Li 200852801, Liangucheng, Minqin, Gansu; 5947: Jianhua Li 200852701, border between Gansu and Inner Mongolia; 5955: Jianhua Li200852703, border between Gansu and Inner Mongolia. Specimens are deposited in Lanzhou University Herbarium.

* Asterisks indicate cox1 intron presence.
