# Supplementary material for: Multiple recent horizontal transfers of the cox1 intron in Solanaceae and extended co-conversion of flanking exons
Source: BMC Evol Biol. 2011 Sep 27;11:277. doi: 10.1186/1471-2148-11-277 (PMC3192709; doi:10.1186/1471-2148-11-277)

Additional file 4. Phylogenetic tree of Brunfelsia spp. based on chloroplast data. Maximum likelihood phylogeny of 7 species of Brunfelsia based on analysis of chloroplast ndhF and trnLF. Numbers above branches are bootstrap support values >50%. GenBank numbers for sequences generated here are shown in boldface. Primers used for sequence amplification are from Olmstead et al [46].

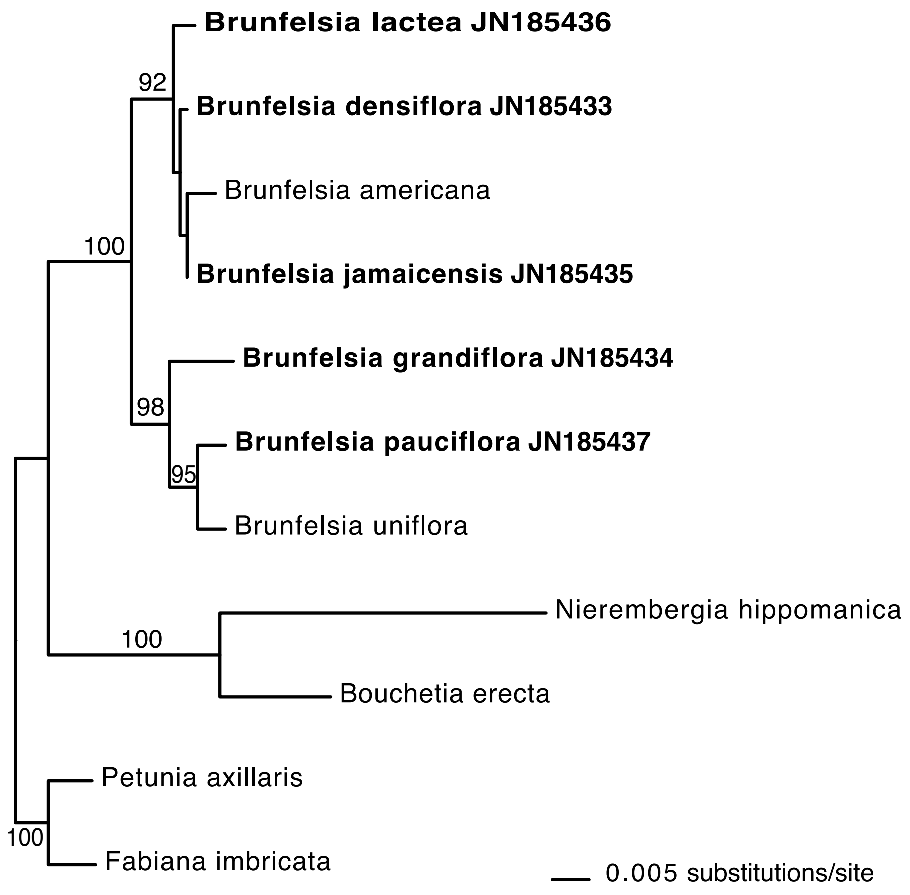

Supplement: Additional file 4 — Phylogenetic tree of Brunfelsia spp. based on chloroplast data. Maximum likelihood phylogeny of 7 species of Brunfelsia based on analysis of chloroplast ndhF and trnLF. Numbers above branches are bootstrap support values > 50%. GenBank numbers for sequences generated here are shown in boldface. Primers used for sequence amplification are from Olmstead et al [46]. [file 1471-2148-11-277-S4.PDF]
